# Statistical learning quantifies transposable element-mediated *cis*-regulation

**DOI:** 10.1186/s13059-023-03085-7

**Published:** 2023-11-10

**Authors:** Cyril Pulver, Delphine Grun, Julien Duc, Shaoline Sheppard, Evarist Planet, Alexandre Coudray, Raphaël de Fondeville, Julien Pontis, Didier Trono

**Affiliations:** 1 School of Life Sciences, Swiss Federal Institute of Technology Lausanne (EPFL), CH-1015 Lausanne, Switzerland; 2 Swiss Data Science Center, Swiss Federal Institute of Technology Lausanne (EPFL), CH-1015 Lausanne, Switzerland; 3 SOPHiA GENETICS SA, La Pièce 12, CH-1180 Rolle, Switzerland

**Keywords:** Transposable elements, Transcription factors, Gene regulation, *Cis*-regulatory elements, Embryogenesis, Gastrulation, Endoderm, Mesendoderm, Germ layer, Gene regulatory networks, Epigenomics, Transcriptomics, Regulatory motif activity, RNA-seq, CRISPRi, CRISPRa, GATA6, EOMES, SOX15, LTR6, LTR5, SVA, PRIMA4-LTR, MER4A, MER4D

## Abstract

**Background:**

Transposable elements (TEs) have colonized the genomes of most metazoans, and many TE-embedded sequences function as *cis*-regulatory elements (CREs) for genes involved in a wide range of biological processes from early embryogenesis to innate immune responses. Because of their repetitive nature, TEs have the potential to form CRE platforms enabling the coordinated and genome-wide regulation of protein-coding genes by only a handful of *trans*-acting transcription factors (TFs).

**Results:**

Here, we directly test this hypothesis through mathematical modeling and demonstrate that differences in expression at protein-coding genes alone are sufficient to estimate the magnitude and significance of TE-contributed *cis*-regulatory activities, even in contexts where TE-derived transcription fails to do so. We leverage hundreds of overexpression experiments and estimate that, overall, gene expression is influenced by TE-embedded CREs situated within approximately 500 kb of promoters. Focusing on the *cis*-regulatory potential of TEs within the gene regulatory network of human embryonic stem cells, we find that pluripotency-specific and evolutionarily young TE subfamilies can be reactivated by TFs involved in post-implantation embryogenesis. Finally, we show that TE subfamilies can be split into truly regulatorily active versus inactive fractions based on additional information such as matched epigenomic data, observing that TF binding may better predict TE *cis*-regulatory activity than differences in histone marks.

**Conclusion:**

Our results suggest that TE-embedded CREs contribute to gene regulation during and beyond gastrulation. On a methodological level, we provide a statistical tool that infers TE-dependent *cis*-regulation from RNA-seq data alone, thus facilitating the study of TEs in the next-generation sequencing era.

**Supplementary information:**

The online version contains supplementary material available at 10.1186/s13059-023-03085-7.

## Introduction

The development and function of complex organisms rely on the tight regulation of gene expression at cellular and tissue levels. *Cis*-regulatory elements (CREs) are non-coding sequences that modulate the transcription of nearby genes in response to signaling cues, thereby contributing to the control of gene expression. Functionally, CREs operate through transcription factor (TF) recruitment and local chromatin remodeling [1]. Importantly, sequence-specific TF-DNA binding allows for the simultaneous regulation of arbitrarily distant genes flanked by CREs carrying analogous TF binding sites (TFBS). Conceptually, the functional interactions implicating CREs, their target genes, and their TF controllers form graph-like representations of the gene expression machinery known as gene regulatory networks (GRNs) [2, 3]. Typically, one may represent CREs as edges connecting two types of nodes: TFs and the protein-coding genes they regulate. According to this view, cell-state and tissue-specific transcriptional programs — defined by specific sets of expressed TFs and accessible CREs — are thereby depicted by distinct GRN topologies. For example, the GRN of so-called primed human embryonic stem cells (hESCs), which resemble epiblast cells of the post-implantation embryo, is characterized by the expression and binding of OCT4, NANOG, and SOX2 to pluripotency-specific CREs [4]. Changes in TF expression can alter GRN topology, thus polarizing cells towards a different state. For example, induced expression of Krüppel-like factor family (KLF) members in primed hESCs alters their GRN towards one resembling that of preimplantation-like “naïve” hESCs notably characterized by increased chromatin accessibility [5, 6].

Whereas the repertoire of expressed TFs and accessible CREs varies across cell states within one organism, the genomic location of CREs with respect to their target genes varies across species. In fact, it has long been recognized that organisms evolve primarily through the emergence, spread, and reorganization of CREs, i.e., modification of GRNs, [2, 7, 8] rather than through mutations affecting protein-coding genes — including TFs — though exceptions to this tenet exist [9]. GRNs may evolve through chromosomal or even genome-wide duplication events followed by divergence and specialization of the henceforth redundant regulatory sub-networks. However, large-scale duplications are too coarse to account for the fine-grained nuances in CRE compositions observed across the genomes of distinct species. Due to their important contribution to the size of most metazoan genomes, their intrinsic ability to recruit TFs and their potential for rapidly spreading ready-to-go regulatory modules throughout the genome of their host, transposable elements (TEs) have gained attention as a potential source of CREs [2, 10, 11].

TEs form a collection of genetic entities that autonomously or collectively code for the factors essential to their own mobility, a process known as transposition. Endogenous retroelements (EREs) propagate through retrotransposition, a copy-and-paste mechanism entailing the reverse transcription of an RNA intermediate encoded within the ERE sequence itself. In agreement with the replicative nature of retrotransposition, EREs constitute the vast majority of the approx. 4.5 million readily recognizable TE-derived sequences that contribute more than half of the human genome DNA content [12, 13]. In contrast, DNA transposons propagate through a non-replicative cut-and-paste process and rely on genome replication to accumulate copies [14]. Both EREs and DNA transposons are further segregated into super/subfamilies [15] forming sets of phylogenetically related integrants that use the same mechanism for transposition [16]. Seminal DNA reassociation studies demonstrated long before the Next Generation Sequencing era that most metazoan genomes were replete with repetitive sequences, some of which emerged in recent evolutionary times. Drawing from this line of work, Britten and Davidson famously reasoned that repetitive DNA may form a pool of potential CREs whose cycles of expansion followed by purifying selection fuels GRN evolution [2]. Consistent with this model, binding sites of conserved TFs and open chromatin regions enrich at evolutionarily young TE subfamilies, in particular in embryonic stem cells (ESCs), and more occasionally in cancer cell lines and lymphoblastoid tissues [17–21]. Moreover, multiple functional studies support the regulatory potential of TEs, including evolutionarily recent integrants. For example, the majority of genes deregulated in humans but not in mouse embryonic stem cells (mESCs) upon knockdown of the master regulator of pluripotency OCT4 are associated with EREs of the ERV1 family, for which an enhancer activity was confirmed by reporter assay [22]. As well, the majority of species-specific enhancers in mouse and rat trophoblast stem cells overlap species-specific TE subfamilies, and a mouse-specific subfamily (RLTR13D5) exhibits trophoblast stem cell-specific enhancer activity in a reporter assay [23]. Finally, the genetic excision of primate-specific MER41B integrants thwarts the functionality of a key innate immunity signaling cascade [24] and hundreds of genes including stemness maintainers are downregulated upon epigenetic repression of the hominoid-specific SVA and LTR5-Hs subfamilies in hESCs [5]. Together, these case studies suggest that evolutionarily recent EREs spread CREs upon which natural selection may act to fine-tune the GRNs of critical physiological processes such as embryogenesis and innate immunity [10, 11, 13]. Despite accumulating evidence that some TE subfamilies form sets of functional CREs, no well-defined and genome-wide statistical framework has been proposed to estimate whether and how much TEs influence the expression of protein-coding genes. In addition, the identification of TE-embedded CREs currently relies on genome-wide epigenomic profiling, typically histone marks, TF binding, enhancer RNA (eRNA) production, and chromatin accessibility [17, 20–22, 25]. While these assays are instrumental to characterize exhaustively the involvement of TEs as CREs under specific biological contexts, performing them in pair with RNA-seq considerably increases experimental costs as well as the biological material required prior to sequencing. Thus, a statistical framework based on RNA-seq alone and capable of estimating which TE subfamilies serve as CREs would benefit the gene regulation research field for hypothesis generation and data interpretation at negligible additional costs.

The hypothesis that TEs influence the expression of protein-coding genes at the subfamily level has a corollary: one should be able to estimate the contribution of TEs to the expression of protein-coding genes by formulating a TE-centric mathematical model of gene regulation from basic principles of gene regulation. Analogous models have been developed to estimate the regulatory activity of TFBS motifs using transcriptomic data [26–29]. These statistical approaches assume that DNA motifs or sequences — typically corresponding to TFBS — may regulate all promoters within which they are present with a quantitatively similar effect on gene expression. By analogy, as TEs evolved to attract the TFs necessary to trigger their own mobility, they can be conceptualized as larger regulatory sequences denoted as TE-embedded regulatory sequences (TEeRS). Thus, we took inspiration from the model of gene regulation championed by Britten and Davidson [2] and hypothesized that phylogenetically related TE integrants may attract similar sets of transcriptional regulators and hence bear a similar regulatory influence on protein-coding genes located in their vicinity. Our system, coined ***craTEs*** (***cis***-*r*egulatory *a*ctivities of *T*ransposable *E*lement **s**ubfamilies), models variations in gene expression as a linear function of the susceptibility of protein-coding genes to the *cis*-regulatory activity of TE subfamilies. Here, we define activity as the variation in gene expression which can be attributed to the presence of integrants belonging to a set of phylogenetically related TEs within *cis*-regulatory distance of the gene promoter. In this study, we assume a priori that TE subfamilies form said sets. *craTEs* thereby enables the identification of *cis*-regulatory TE subfamilies from RNA-seq data alone, rooting it in the expression profile of protein-coding genes. Thus, *craTEs* adheres to a strict definition of *cis*-regulatory activity which requires an associated change in gene expression, in contrast with approaches restricted to profiling biochemical activity at TE loci [18–21, 25].

In this study, we first show that *craTEs* accurately identifies *cis*-regulatory TE subfamilies from RNA-seq data alone. We demonstrate that it achieves this feat agnostically with respect to TE-derived transcription, with increased statistical power compared with standard enrichment-based approaches, and in cases where changes in transcription at the corresponding subfamilies remain undetectable. We then leverage *craTEs* in conjunction with a large-scale TF perturbation RNA-seq dataset to estimate the maximal genomic distance up to which *cis*-regulatory TEs measurably contribute to the regulation of transcription genome-wide. Using the same dataset complemented with TF binding profiles and context-relevant TF knockout (KO) studies, we then identify novel regulatory links between TF expression and *cis*-regulatory TE activities throughout embryogenesis. Finally, we verify that *craTEs* detects biologically relevant regulatory phenomena by performing DNA binding, histone mark, and chromatin accessibility profiling experiments. Overall, we present and validate *craTEs*, a simple mathematical model of TE-dependent gene regulation. *craTEs* recapitulates the findings of landmark case studies of TE-dependent *cis*-regulation and suggests previously unappreciated regulatory ties implicating TFs and TEs, particularly during and beyond gastrulation. These results support a model of GRN evolution whereby the spread of TEs provides an important supply of raw regulatory materials.

## Results

### *craTEs* models variations in gene expression as a linear combination of TE-encoded *cis*-regulatory elements

**Fig. 1:**
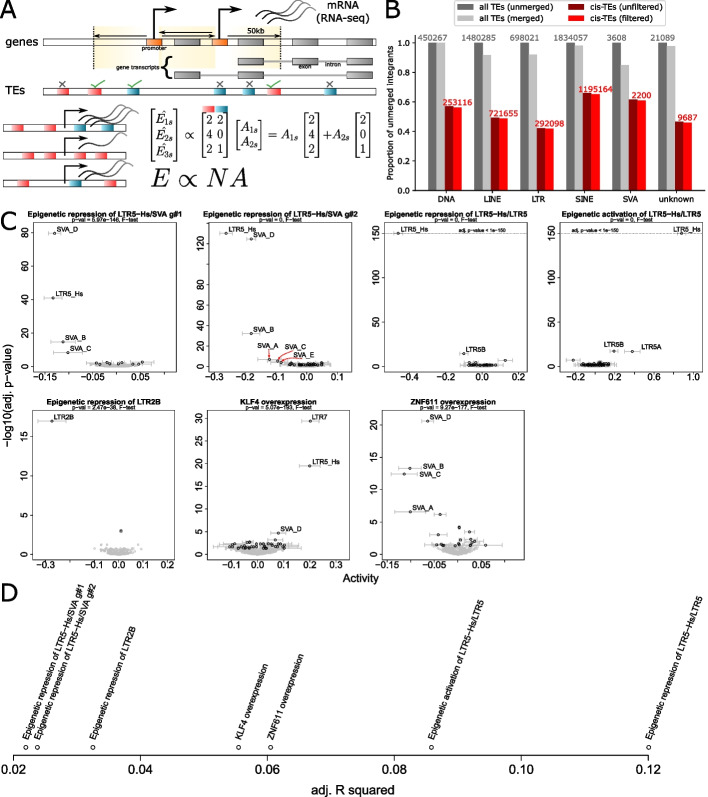
*craTEs* uncovers *cis*-regulatory TE subfamilies from RNA-seq. **A** Overview of the *craTEs* model. Differences in expression [log(TPM)] for protein-coding genes between treatment and control samples (columns of matrix *E*) are modeled as a linear combination of the per-subfamily TE counts found in the *cis*-regulatory region (shaded beige) of each gene (columns of *N*). Differences in *cis*-regulatory activities for each treatment vs. control experiment (columns of *A*) are estimated by least squares. The *cis*-regulatory regions of each gene are defined as 50-kb long stretches of DNA 5′ and 3′ from promoter regions. *Cis*-regulatory regions exclude the exons (gray boxes) and promoters (orange boxes) of the genes they are assigned to. Gray bold lines: gene introns. Sequences of introns and exons: transcripts. **B** Proportion of integrants remaining at each step of the construction of N with respect to the original number of TEs present in the annotation (indicated in gray). “All TEs” refers to all integrants found in the TE database “Repeatmasker RELEASE 20170127” (number of unmerged TEs are indicated in gray). “cis-TEs” refers to integrants found in *cis*-regulatory regions before (“unfiltered”) and after (“filtered”, numbers indicated in red) removing those overlapping exons and promoters of the corresponding gene. **C** Seven case studies exemplifying the estimation of the *cis*-regulatory activities of TE subfamilies from RNA-seq data. Black dots are TE subfamilies with statistically significant (BH-adj. *p*-value <0.05, *t*-test) differences in activities between the treatment and control groups. 95% confidence intervals for the estimated *cis*-regulatory activities are shown as gray bars. Gray dots are TE subfamilies with non-significant differences in activities. Subtitle: *p*-value from the F-test of overall significance in regression. From left to right: CRISPRi-mediated repression of LTR5-Hs and SVA integrants in naïve hESCs, gRNA #1 (g#1) $n=3$ (3 treatment samples vs. 3 control samples) [33]; CRISPRi-mediated repression of LTR5-Hs and SVA integrants in naïve hESCs, gRNA #1 (g#1) $n=3$; CRISPRi-mediated repression of LTR5-Hs/A/B integrants in an embryonal carcinoma cell line (NCCIT), $n=2$ [35]; CRISPRa-mediated activation of LTR5-Hs/A/B integrants in NCCIT, $n=2$; CRISPRi-mediated repression of LTR2B integrants in K562, $n=2$ [34]; overexpression of the pluripotency TF KLF4 in primed hESCs, $n=4$ [33]; overexpression of the SVA-targeting KZFP ZNF611 in naïve hESCs, $n=2$ [33]. **D** Proportion of variance of *E* explained by *craTEs* for each experiment in **C**

Using RNA-Seq data, we aimed to systematically uncover TE subfamilies that regulate the expression of protein-coding genes in *cis*. Integrants of the same TE subfamily share a high level of sequence similarity. Thus, they are predicted to exert a similar *cis*-regulatory influence on protein-coding genes located in their vicinity, for example through the simultaneous recruitment of a specific set of transcriptional regulators at multiple genomic loci. We thus assumed that the subfamily composition of TE integrants located within *cis*-regulatory distance of protein-coding genes contributes to a discernible fraction of the variation in gene expression (Fig. 1A) [27, 29]. As a first approach, we set this distance to 50 kb since differentially expressed (DE) genes were found to be enriched within this range of epigenetically perturbed *cis*-regulatory LTR5-Hs and SVA TE subfamilies [5].

Considering two experimental conditions denoted as 1 and 2, for example, “control cells” and “cells with transgene overexpression”, we modeled the variation in gene expression $\Delta E$ of each of the *p* protein-coding genes as a linear combination of the per-subfamily TE integrant counts $N_{pm}$ located within *cis*-regulatory distance of its promoters (see Methods). $N_{pm}$ represents the regulatory susceptibility [27] of gene *p* to TE subfamily *m*. We trade biological complexity for statistical simplicity by treating members of the same TE subfamily as “regulatory black boxes” of equal *cis*-regulatory potential. A well-known caveat of currently available ERE annotations is that integrants are often fragmented into multiple sequences [13, 30], causing an artificial inflation of $N_{pm}$ and potentially deteriorating model performances. We therefore merged closely located (< 100 bp) ERE fragments of the same subfamily into single ERE integrants. LINEs, LTRs, and SVAs were particularly prone to spurious fragmentation (Fig. 1B), with numbers of integrants dropping by 8.3%, 7,9%, and 15%, respectively, after merging. To define the regulatory susceptibility $N_{pm}$ of each gene *p* to each TE subfamily *m*, we counted the number of integrants of subfamily *m* falling within *cis*-regulatory distance of the promoters of *p*. We found that between 45.9% (LTRs) and 72.5% (SVAs) of all integrants were located within *cis*-regulatory distance, i.e., 50 kb up/downstream, of at least one protein-coding gene promoter (Fig. 1B). In rare instances, TEs overlap gene exons. Since these are used to quantify RNA-seq reads, this may introduce a spurious association between the presence of an annotated TE integrant and gene expression. We addressed this by excluding TEs overlapping exons from the set of putatively *cis*-regulatory TEs susceptible to regulate the corresponding gene. Finally, we chose to emphasize TE-driven *cis*-regulation dependent on distal sequences, i.e., located more than 1.5kB up/downstream of a transcription start site, as the role of TEs as alternative promoters has been extensively studied elsewhere [31, 32]. Thus, we prevented TEs overlapping with promoters of gene *p* from contributing to the set of regulatory susceptibilities $N_{pm}$ (Fig. 1A–B). The combination of the last two filtering steps excluded 1.2% of TEs found within *cis*-regulatory distance of protein-coding genes from *N* (Fig. 1B).

The main purpose of *craTEs* is the estimation of $\Delta A_{m\;2-1}$ which we define as the difference in *cis*-regulatory activity exerted by subfamily *m* between conditions 1 and 2 (see Eq. 1). For the purpose of this study, we chose the convention that a positive *cis*-regulatory activity refers to an “enhancer-like” effect in condition 2 with respect to condition 1. Conversely, a negative *cis*-regulatory activity may reflect either the gain of a “silencer-like” effect or the loss of an “enhancer-like” effect in condition 2 versus condition 1. The *cis*-regulatory activity $\Delta A_{m\;2-1}$ has an intuitive interpretation: it is the quantity in expression that would be gained by any gene in condition 2 with respect to condition 1 upon insertion of an integrant of subfamily *m* within *cis*-regulatory distance of one of its promoters. An independently and identically distributed Gaussian noise term centered around zero $\epsilon _{2 + 1}$ captures the variation in gene expression that is not accounted for by the linear model, and represents the sum of the noise terms corresponding to gene expression in each condition.

1 $$\begin{aligned} \Delta E_{p,2-1} = \Delta A_{0, 2-1} + \sum _{m} N_{pm} \Delta A_{m,2-1} + \epsilon _{2+1} \end{aligned}$$

*craTEs* estimates the vector of *cis*-regulatory TE subfamily activities $\hat{\Delta A_{2-1}}$ by minimizing the squared difference between the observed logged expression values $\Delta E_{2-1}$ and those modeled as linear combinations of the columns of the susceptibility matrix *N*, containing the regulatory susceptibilities $N_{pm}$. Furthermore, *craTEs* assesses whether there is statistical evidence that $\hat{\Delta A_{m\;2-1}}$ differs from zero: each component of $\hat{\Delta A_{2-1}}$ is tested against the null hypothesis $H_0: \Delta A_{m\;2-1} = 0$, i.e., that there is no difference in activity between conditions 1 and 2 for subfamily *m*, by means of a *t*-test (see the “Methods” section). This provides a measure of statistical significance for the estimated differences in TE-dependent *cis*-regulatory activities between conditions 1 and 2.

### *craTEs* uncovers *cis*-regulatory TE subfamilies from RNA-seq data

We then assessed the ability of *craTEs* to detect *cis*-regulatory TE subfamilies under controlled experimental settings. In particular, we leveraged three RNA-seq datasets derived from experiments in which specific TE subfamilies were epigenetically silenced or activated, thus ablating their *cis*-regulatory effect on neighboring protein-coding genes [33–35]. These datasets provide a biological “ground truth” against which we evaluated the output of *craTEs*. The targeted epigenetic modulation of specific genomic loci was achieved by means of the CRISPR interference or activation systems [36]. CRISPRa/i relies upon a catalytically dead Cas9 domain (dCas9) that binds to DNA sequences complementary to user-defined guide RNAs (gRNAs). Once bound to the DNA, the dCas9-fused KRAB domain elicits the local deposition of repressive histone marks, thereby suppressing any enhancer activity exerted by the target site (CRISPRi). Conversely, the dCas9-fused VPR transactivator domain recruits a diverse set of transcriptional activators encompassing histone acetyltransferases upon DNA binding, thereby stimulating enhancer activity at the target site (CRISPRa) [37, 38]. As TEs of the same subfamily exhibit high levels of sequence similarity, hundreds of related integrants can be targeted for activation/silencing by only a handful of carefully designed gRNAs [5, 39, 40]. We have previously shown that the hominoid-specific LTR5-Hs and SVA TE subfamilies serve as enhancers in naïve hESCs and that this *cis*-regulatory activity can be ablated by CRISPRi [5]. We reanalyzed RNA-seq data from naïve hESCs where large fractions of the LTR5-Hs and SVA subfamilies were epigenetically silenced via CRISPRi across two independent experiments, each through a distinct guide RNA (g#1 and g#2). We applied *craTEs* to the vector $\Delta E_{CRISPRi -control}$ containing the differences in gene expression between each CRISPRi experiment and control naïve hESCs. The association between the differences in gene expression $\Delta E_{CRISPRi -control}$ and the *cis*-regulatory susceptibilities *N* of promoters to TE subfamilies was statistically significant (g#1: *p*-val = $5.47\cdot 10^{-146}$, *F*-test, g#2: *p*-val = 0, *F*-test), strongly suggesting an interrelation between changes in expression observed at protein-coding genes and the genomic distribution of integrants for at least a subset of all TE subfamilies. After correcting for multiple testing using the Benjamini-Hochberg procedure [41], we uncovered 13 (g#1), resp. 39 (g#2) TE subfamilies with statistically significant differences in *cis*-regulatory activity (Fig. 1C, Table S1), i.e., non-zero $\Delta A_{m, 2-1}$ activity coefficients. Among these, LTR5-Hs, SVA B, C, and D subfamilies displayed the largest and most statistically significant absolute estimated *cis*-regulatory activities. The negative activity values reflect the abrogation of the enhancer effect exerted by LTR5-Hs and SVAs in naïve hESCs by the CRISPRi system and are best interpreted as the *log*2 fold-change in protein-coding gene expression attributable to the presence of a single integrant from the corresponding subfamily near the promoter of the given gene. Specifically, the expression of any given gene bearing an LTR5-Hs integrant in its *cis*-regulatory window decreases by an estimated fold-change contained within the 95% confidence interval $2^{-0.133\pm (1.96\cdot 0.0095)} = [0.90;0.92]$, i.e., by approximately 10% upon CRISPRi using g#1 (Table S1). We then applied *craTEs* to RNA-seq data generated from the CRISPRi-mediated repression of LTR5-Hs, LTR5A and LTR5B in the NCCIT human embryonal carcinoma cell line [35] and found that LTR5-Hs and LTR5B showed the largest and most statistically significant absolute differences in *cis*-regulatory activity (Fig. 1C), with the related LTR5A subfamily also displaying a weaker yet also statistically significant difference (Table S1). Conversely, *craTEs* uncovered LTR5-Hs, LTR5A, and LTR5B as the TE subfamilies with the largest and most statistically significant absolute difference in *cis*-regulatory activity when applied to RNA-seq derived from NCCIT cells subjected to LTR5-Hs/LTR5A/LTR5B CRISPRa (Fig. 1C), this time with positive activities mirroring the increased enhancer effect exerted by CRISPRa-targeted LTR5-Hs/LTR5A/LTR5B on neighboring genes. Thus, *craTEs* correctly inferred gains and losses of enhancer effect at the subfamilies targeted by CRISPRa/i and did so from the expression of protein-coding genes alone.

As it is well established that TEs are particularly active in hESCs [6, 42], we wondered whether *craTEs* would be able to recover TE-dependent *cis*-regulatory changes in other cellular contexts. A subset of LTR2B elements are marked by the enhancer histone mark H2K27ac in various leukemia cell lines, including in chronic myelogenous leukemia-derived K562 cells [43]. We used *craTEs* to estimate the differences in TE-driven *cis*-regulatory activities between K562 cells where LTR2B were repressed via CRISPRi and their control counterparts. *craTEs* correctly identified LTR2B as significantly less active in LTR2B-CRISPRi K562 cells compared to control K562 cells (Fig. 1C). Thus, *craTEs* recovers TE-dependent *cis*-regulatory mechanisms beyond the context of hESCs.

Next, we empirically verified whether the ability of *craTEs* to detect changes in *cis*-regulatory TE activity is generalized beyond experiments of targeted TE repression via CRISPRi. TEs often exert *cis*-regulatory effects by serving as docking platforms for TFs. For example, the core pluripotency TF KLF4 is highly expressed in naïve hESCs, where it binds to LTR7, LTR5-Hs, and SVA integrants [5]. Interestingly, these subfamilies also display elevated levels of the enhancer histone mark H3K27ac in naïve hESCs. In contrast, primed hESCs generally express lower levels of KLF4 and TEs than their naïve counterparts [5, 6]. Using *craTEs*, we assessed the impact of KLF4 overexpression on TE-dependent *cis*-regulation in primed hESCs. *craTEs* identified LTR7, LTR5-Hs, and SVA D as the most statistically significant and highly activated TE subfamilies upon KLF4 overexpression, thereby recapitulating our previous findings [5] agnostically with respect to epigenomics data and TE-derived transcripts (Fig. 1C). Interestingly, we previously observed that the KLF4-dependent enhancer activity of SVAs in primed hESCs did not correlate with increased SVA transcription (Fig. S1A) but instead with an accumulation of H3K27ac enhancer histone marks at SVA integrants [5]. This suggests that *craTEs* detects TE-dependent *cis*-regulatory effects that would not be inferred from studying the variation in the expression of TE integrants. Furthermore, overexpression of the repressive SVA-binder KRAB-zinc finger protein ZNF611 [9] in naïve hESCs abrogates the enhancer activity of SVAs [5]. We used *craTEs* to estimate the differences in TE-dependent *cis*-regulation between ZNF611-overexpressing and control naïve hESCs. As expected, *craTEs* identified SVAs as the TE subfamilies with the most statistically significant and largest absolute differences in *cis*-regulatory activity between the two settings (Fig. 1C), with negative activity values reflecting the loss of enhancer effect at SVAs upon ZNF611 overexpression. Of note, the proportion of the variance in gene expression explained by the distribution of TEs across *cis*-regulatory windows [2–12%, adj. $R^2$] (Fig. 1D) overlapped with the typical fraction of explained variance reported upon modeling gene expression as a function of the distribution of TF binding motifs at gene promoters [29]. Together, these results show that *craTEs* correctly identifies TE-dependent *cis*-regulatory activity changes beyond the context of targeted TE epigenetic perturbations and demonstrate its utility for identifying TE-dependent regulatory mechanisms under biological perturbations that affect TEs indirectly. In addition, *craTEs* identifies *cis*-regulatory TE subfamilies without resorting to mapping RNA-seq reads emanating from transcriptionally active TEs or performing epigenomics assays.

### *craTEs* outperforms enrichment approaches based on differential expression analyses

The notion that differences in gene expression may reveal candidate *cis*-regulatory TEs has already been exploited in previous studies [5, 44] though the statistical methodologies differ from *craTEs* in key aspects. More specifically, these methods identify *cis*-regulatory TEs through a two-step process. First, differentially expressed (DE) genes are identified through ad hoc statistical methods [45, 46]. Then, per-subfamily scores for the enrichment of differentially expressed genes in the vicinity of TE integrants are computed. A high enrichment is reflected by a small probability (*p*-value) of finding more DE genes in the vicinity of a specific subfamily than the observed number of DE genes. We empirically compared the output of *craTEs* with that of the enrichment approach on the RNA-seq dataset whereby LTR5-Hs/SVA were silenced via CRISPRi [33]. Using the enrichment approach, we found that DE genes whose expression fell under LTR5-Hs/SVA epigenetic repression (Fig. S1B, *p*-val <0.05, Fisher’s exact test, lenient DE calling) were statistically significantly enriched in the vicinity of LTR5-Hs, SVA B and SVA D integrants (Table S2, BH-adj. *p*-val <0.05, hypergeometric test). Note that the DE enrichment approach failed to detect the regulatory link between gene downregulation and the TE subfamily SVA C (adj. *p*-val = 1, hypergeometric test), whereas these were identified by *craTEs* (Fig. 1C, Table S1). Moreover, when correcting for multiple testing during differential expression analysis (Fig. S1B, BH-adj. *p*-val <0.05, Fisher’s exact test, stringent DE calling), DE genes were enriched near SVA D integrants, but not LTR5-Hs or other SVA subfamilies (Table S3). These results indicate that *craTEs* is more sensitive than DE enrichment approaches in the task of detecting *cis*-regulatory TE subfamilies from the expression of protein-coding genes. To assess whether this came at the cost of decreased specificity, we quantified the ability of *craTEs* — resp. the DE enrichment approach — to recover a ground truth set of *cis*-regulatory TE subfamilies upon CRISPRi-mediated repression of LTR5-Hs and SVAs either using g#1 or g#2 (Figs. 1C, S1D). Since no epigenomic data was available for g#1 [33], we leveraged ATAC-seq to generate chromatin accessibility profiles in naïve hESCs subjected to g#1-mediated CRISPRi against LTR5-Hs/SVAs. We then defined ground truth *cis*-regulatory TE subfamilies for each gRNA as those (1) with integrants directly targeted by the gRNA (LTR5-Hs, SVA A-F) (2) with enrichment for decreased ATAC-seq (g#1: {LTR5-Hs, SVA A-D}, Table S4; g#2: {LTR5-Hs, SVA A-F}, Table S5) and/or increased H3K9me3 upon CRISPRi (g#2: {LTR5-Hs, SVA A-F}, Table S6). We used the subfamily-specific BH-adjusted *p*-values computed according to *craTEs*, the lenient and the stringent DE enrichment approaches to classify subfamilies into two classes — *cis*-regulatory versus not *cis*-regulatory and subsequently computed the area under the receiver operating characteristic curves (AUCs) (Fig. S1C). *craTEs* displayed higher AUCs than DE enrichment approaches for both gRNAs (g#1: 0.996 vs. {0.800, 0.600}, g#2: 1.0 vs. {0.85, 0.88}), noting that the low rates of true positives versus true negatives may partially explain the elevated AUCs. Overall, this suggests that by pooling information across all genes, and not just DE genes, *craTEs* offers increased statistical power over classical DE enrichment approaches in the task of identifying *cis*-regulatory TE subfamilies. Moreover, this emphasizes that *cis*-regulatory subfamilies as identified by *craTEs* agree with those displaying an enrichment based on differential context-matched epigenomic data.

*craTEs* estimates *cis*-regulatory TE activities by considering expression variations across hundreds of protein-coding genes. Consequently, *craTEs* does not require replicates to estimate TE subfamily *cis*-regulatory activities. To illustrate this, we reanalyzed the RNA-seq data derived from LTR5-Hs/SVA CRISPRi experiments [33]. We treated each pair of LTR5-HS/SVA CRISPRi and control samples as a single experiment, in effect ignoring the information provided by the replicate structure. We applied *craTEs* to each of the four replicates for both gRNAs (Fig. S1D). Consistent with our findings while accounting for replicates (Fig. 1C), LTR5-Hs and SVA subfamilies collectively exhibited a statistically significant decrease in *cis*-regulatory activity upon CRISPRi across replicates, though not all of them passed the significance threshold. In addition, classifying subfamilies as *cis*-regulatory based on the measure of statistical significance reported by *craTEs* (BH-adj. *p*-val, *t*-test) yielded AUCs ranging from 0.87 to 0.99. Thus, whereas the discovery power of *craTEs* grows together with the number of replicates, the method can still uncover statistically significant changes in the *cis*-regulatory activities of TEs even in the absence of replicates. In contrast, any DE enrichment approach requires at least three samples due to the prerequisites of the DE analysis methods [45, 46], and therefore cannot perform better than a random classifier in the absence of replicates (AUC = 0.5). In addition, *craTEs* not only quantifies the statistical significance of TE subfamily *cis*-regulatory activities but also provides a measure of the effect size through the estimated coefficient $\hat{\Delta A_{m,2-1}}$ which can be interpreted as the *log*2 fold-change in gene expression that would affect any gene upon insertion of an integrant from subfamily *m* within its *cis*-regulatory window (Fig. 1A). Overall, this case study suggests that *craTEs* is more powerful and more informative than DE-based enrichment approaches to discover *cis*-regulatory TE subfamilies from RNA-seq data, supporting the notion that TEs act as *cis*-regulatory fine-tuners, the dynamics of which may be overlooked when restricting the analysis to DE genes only.

### Influential TE-embedded *cis*-regulatory information resides up to 500 kb from gene promoters

**Fig. 2:**
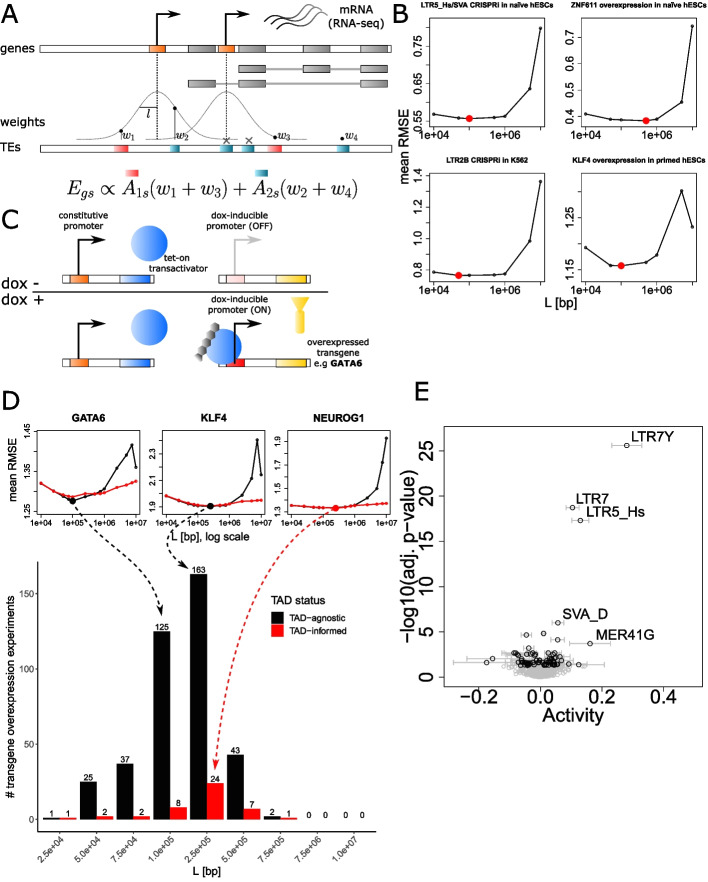
Influential TE-embedded *cis*-regulatory information resides up to 500kb from gene promoters. **A** Overview of the weighting process whereby the *cis*-regulatory influence of TEs decreases as a function of the distance to the closest promoter. The scheme depicts a protein-coding gene with two alternative promoters (in orange), coding for two alternative isoforms (in gray). Gaussian kernels with a maximum value of 1 and of varying bandwidth *L* are centered on each promoter. Before being added to the corresponding element in the matrix *N*, each TE is weighted as a function of its distance to the closest gene promoter. TEs overlapping exons (gray boxes) and promoters (orange boxes) of the gene are excluded. **B** To find the bandwidth *L* leading to the smallest prediction error, the root-mean-squared error (RMSE) was computed for each validation fold and averaged across the five folds over different values of *L*. **C** Overview of the experimental design of the hESC “perturbome” [50]. hESC cell lines carrying a stably integrated dox-inducible transgene overexpression construct were established from individual cells. In each of the 441 transgene overexpression experiments, dox-treated samples (dox+) are compared to the same cell line in the absence of dox (dox−). Note that the number of replicates per experiment varies. **D** Histogram depicting the number of times each Gaussian kernel bandwidth *L* — either TAD-informed or agnostic — led to the smallest mean validation RMSE in a 5-fold cross-validation scheme for the 441 transgene overexpression experiments. TAD-informed (red): the *cis*-regulatory weights linking integrants to genes were restricted by topologically associating domain (TAD) boundaries. TAD-agnostic (black): TAD boundaries were not considered. Individual mean RMSE estimations for GATA6, KLF4, and NEUROG1 are shown as illustrative examples. **E** Estimation of the *cis*-regulatory activity of TE subfamilies upon KLF4 overexpression [33] using the matrix *N* computed with $L=250$ kb

In a first implementation of *craTEs*, we defined *cis*-regulatory regions as 50-kb-long stretches of DNA directly adjacent to the 5′ and 3′ sides of protein-coding gene promoters. Though informed by previous work [5], this choice of genomic distance was based upon data corresponding to LTR5-Hs and SVA subfamilies in hESCs only and may not reflect the general range of action of *cis*-regulatory TEs across all subfamilies and cellular contexts. We therefore modified the *craTEs* model to weight the regulatory influence of each integrant *i* on each gene *p* as a continuous and decreasing function of its distance $d_{p,i}$ to the closest promoter of *p* (Fig. 2A). We defined the regulatory susceptibility $N_{pm}$ as:

2 $$\begin{aligned} N_{pm} = \sum _{i\in \{m,c\}}e^{-\frac{-d_{p,i}^2}{2L^2}} \end{aligned}$$

where each weight was computed using a Gaussian kernel applied to the integrant-promoter distances $d_{p,i}$. We considered all combinations of genes and integrants located on the same chromosome *c*. Note that integrants falling within exons or promoters of *p* were excluded from $N_{pm}$. We computed 11 susceptibility matrices *N* by varying the bandwidth of the Gaussian kernel *L* between 1 kilobase (kb) and 10 Gigabases (Gb) thus spanning the entire range of possible *cis*-regulatory distances. Setting *L* to 1kb restricts *cis*-regulatory regions to the direct vicinity of gene promoters. In contrast, at 10 Gb, *L* exceeds the length of human chromosomes by two orders of magnitude, thus yielding nearly equal regulatory susceptibility scores across genes located on the same chromosome (Fig. S2A). We then tested which of these 11 matrices led to the smallest prediction error using 5-fold cross-validation. For the LTR5-Hs/SVA epigenetic repression, ZNF611 overexpression, KLF4 overexpression [33], and LTR2B epigenetic repression experiments [34], the validation error was minimized for $L = 100$ kb or $L = 500$ kb (Fig. 2B). As 95% of the area under a Gaussian curve is contained within two standard deviations from its mean (Fig. S2B), this suggests that TEs encode discernible *cis*-regulatory information up to distances of approximately 200 kb to 1 million bases (Mb) from gene promoters. We note that errors estimated for small (1 kb) and very large ($>= 100$ Mb) values of *L* were unstable due to the high degree of collinearity between predictors. Indeed, a small *L* results in high numbers of zero-inflated columns in the *N* matrix. Conversely, very large values of *L* yield nearly equal weights for TE-gene pairs located on the same chromosome (Fig. S2A). Both cases make the least squares problem ill-posed by making the matrix *N* singular.

We wondered whether the optimal *cis*-regulatory bandwidths estimated from the four datasets treated thus far (Fig. 2B) generalized to other TE subfamilies as well. We took advantage of a recently published RNA-seq dataset where hundreds of transgenes, mostly TFs, were overexpressed in primed hESCs through a dox-inducible system [47] (Fig. 2C). We considered this dataset as a “perturbome” where each overexpressed transgene polarizes the primed hESC transcriptome towards a specific direction, e.g., towards the naïve hESC GRN or down a differentiation path. We used the same 5-fold cross-validation scheme to find the optimal value of *L* for each transgene overexpression experiment (Fig. 2D), this time comparing the prediction error for gene expression between *cis*-regulatory weight assignment informed by versus agnostic to hESC-specific topologically associating domains (TADs) [48]. In 436/441 transgene overexpression experiments, the optimal bandwidth *L* took values between 50 kb and 500 kb. As most of the area below a Gaussian curve is contained within two bandwidths from its mean (Fig. S2B), TE subfamilies encode *cis*-regulatory information up to distances comprised between 100 kb and 1 Mb from the promoters of protein-coding genes in hESCs. In 187/441 transgene overexpression experiments, $L = 250$ kb led to the smallest cross-validation error, the majority of which (163/187) did not benefit from TAD-informed *cis*-regulatory weight assignment (Fig. 2D), although the performance gap separating TAD-agnostic versus TAD-informed TE subfamily activity estimation was modest, as illustrated for GATA6, KLF4, and NEUROG1. Thus, TAD-agnostic *cis*-regulatory weight assignment according to a bandwidth of $L = 250$kb can be chosen to weight *cis*-regulatory TE integrants such as to maximize predictability. As an illustration, with $L=250$ kb, a TE located 250 kb away from a gene promoter receives a weight of 0.61 (Fig. S2B). The weight drops to 0.01 for a TE-promoter distance of 750 kb resulting in a virtually negligible contribution to the *craTEs* model.

A predictor matrix *N* based on TE contributions weighted by their distance to protein-coding genes (Fig. 2A) has two potential advantages over a predictor matrix *N* computed from hard distance thresholds as we did when first validating the discovery power of *craTEs* (Fig. 1A). First, the quality of the predictors is likely to improve, as the optimal distance until which *cis*-regulation affects gene expression is estimated directly from the expression data. In other words, a continuous and decreasing weighting function may better represent the regulatory potential of TEs on protein-coding genes than a hard threshold approach. Second, as we require that each TE subfamily included in *N* sums up to a total regulatory potential greater than 150 (see the “Methods” section), the continuously decreasing weighting approach may allow for the inclusion of more TE subfamilies in the columns of *N*, leading to the discovery of previously overlooked statistically significant *cis*-regulatory TE subfamilies. We used the KLF4 overexpression RNA-seq dataset we previously generated [33] to illustrate these points. We replaced the regulatory susceptibilities $N_{pm}$ of matrix *N* computed according to a hard distance threshold (Fig. 1A) with those corresponding to the same subfamilies, this time computed either through TE-promoter distance weighting ($L=250$ kB) or according to an approximately equivalent hard-thresholded *cis*-regulatory window width of 500 kB (Fig. 2A, Eq. 2). All three models thus use the exact same number of predictors, i.e., cover the same TE subfamilies. Running *craTEs* with the weighted matrix *N* computed with $L=250$ kb increased the fraction of gene expression variation explained from 4.5 to 5.4% compared to using the matrix *N* derived from 100-kB-wide hard-thresholded *cis*-regulatory windows. As the number of predictors in *N* remained unchanged, this suggests that the distance weighting approach better approximates the *cis*-regulatory potential of TE subfamilies than the hard distance threshold approach. Notably, as 500-kB-wide hard-thresholded *cis*-regulatory windows explained 5.2% of the variance in gene expression, most of the increase in explained variance observed under the weighted ($L=250$ kB) versus the unweighted model (Fig. 1) likely stems from considering more distant TEs as putatively *cis*-regulatory. Next, we empirically evaluated whether allowing for the inclusion of TE subfamilies that passed the minimum per-subfamily regulatory potential with distance weighting (Eq. 2) — but not with hard distance thresholding — would uncover additional biologically validated TE-dependent *cis*-regulatory changes. LTR7Y was identified as the most statistically significantly activated subfamily upon KLF4 overexpression in hESCs (Fig. 2E), in agreement with previously published results [5] while it was absent from the model specified through hard distance thresholding (Fig. 1C, Table S1). In addition, though also absent from the hard distance thresholding model, the primate-specific MER41G subfamily was found as statistically significantly and strongly activated in the distance-weighted model. Regarding the LTR5-Hs/SVA CRISPRi, ZNF611 overexpression, and LTR2B CRISPRi experiments, using the distance-weighted matrix *N* still uncovered LTR5-Hs/SVAs, LTR2B, resp. SVAs as differentially *cis*-regulatory (Fig. S2E). To sum up, TE subfamilies typically encode *cis*-regulatory potential up to distances of approx. 500 kb from the promoters of protein-coding genes, at least in the context of hESCs. This reinforces the notion that TEs form a layer of regulatory fine-tuners exerting a measurable impact on the expression of protein-coding genes.

### TFs controlling gastrulation and organogenesis promote the *cis*-regulatory activity of evolutionarily young TE subfamilies activated during pluripotency

**Fig. 3:**
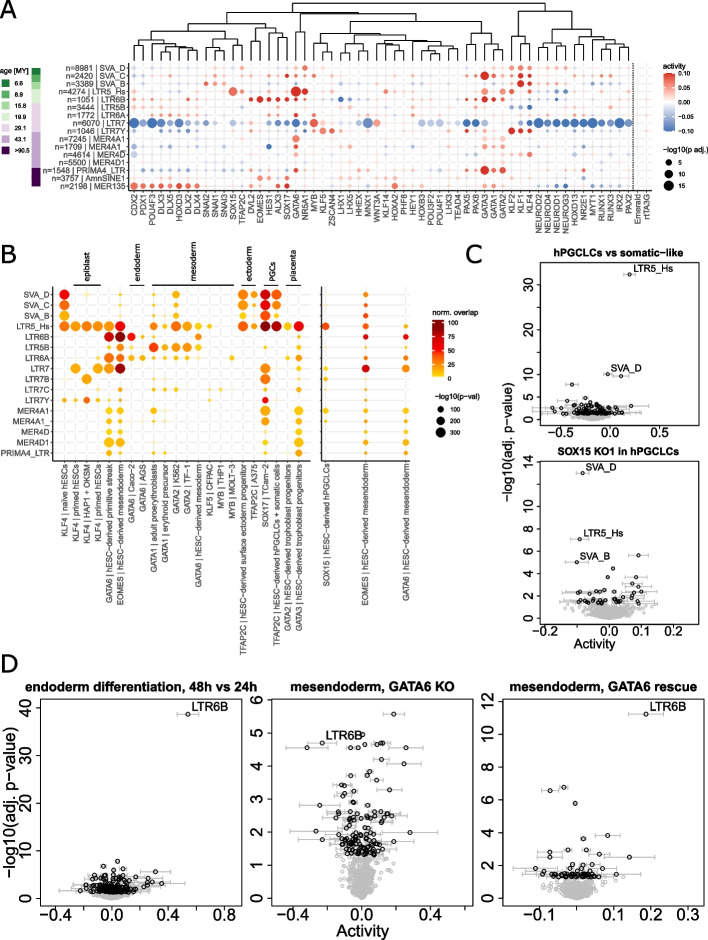
TFs controlling gastrulation and organogenesis promote the *cis*-regulatory activity of evolutionarily young TE subfamilies activated during pluripotency. **A** TE subfamily *cis*-regulatory activities (color: activity coefficients; area: statistical significance) estimated from dox-induced transgene overexpression experiments at 48 h in primed hESCs [47] using *N* computed with *L* = 250 kb. The number of replicates for each condition varies. Experiments were clustered using complete linkage hierarchical clustering on Euclidean distances computed from activity coefficients. Selected TE subfamilies were ordered by evolutionary age in millions of years, as previously estimated [5]. The number of protein-coding genes with total *cis*-regulatory weights > 0.13 (weight obtained at a distance of 2L, see Fig. S2) is shown for each subfamily. The color labeling of the estimated activities was saturated at $|\Delta A| < 0.1$. **B** Top binding enrichment at selected evolutionarily young TEs by selected TFs controlling germ layer development. TEs (rows) were ordered as in **A**. ChIP-seq experiments (columns) were ordered by developmental stage or germ layer lineage. Color: number of peaks overlapping with subfamily-specific integrants, normalized for subfamily size. Area: statistical significance. Left: ChIP-seq peaks obtained from the ChIP-Atlas [62]. Right: ChIP-seq peaks obtained from [86] and [70]. **C** Top: Estimated differences in TE-dependent *cis*-regulatory activities between hESC-derived EpCAM$^+$/INTEGRIN$\alpha$6$^+$ double positive (DP) hPGCLCs and double negative (DN) somatic cells at day 6 of differentiation, replicate #1 $n=2$ [86]. Bottom: SOX15 KO DP hPGCLCs vs. DP hPGCLCs, day 6, $n=2$. **D** Left: hESC-derived differentiating endoderm, 48 h vs 24 h, $n=3$ [70]. Middle: iPSC-derived GATA6 KO mesendoderm vs. iPSC-derived mesendoderm, $n=2$ [69]. Right: GATA6 rescue in iPSC-derived GATA6 KO mesendoderm vs. iPSC-derived GATA6 KO mesendoderm, $n=2$

Having validated the ability of *craTEs* to agnostically recover well-established cases of TE-dependent *cis*-regulatory activities [5, 39, 43], we next aimed at characterizing the landscape of TF-induced TE-dependent *cis*-regulation in primed hESCs. As the epigenome of hESCs is markedly more open than that of differentiated cells [49], the number and strengths of the TF-TE regulatory interactions constituting the GRN of hESCs can be understood as upper bounds on those constituting the GRNs of differentiated tissues. We therefore applied *craTEs* to the “perturbome” dataset, where 441 transgenes, most of them TFs, were individually overexpressed in primed hESCs for 48 h through a dox-inducible system (Fig. 2C) [47]. Using the regulatory susceptibility matrix *N* computed according to the best-performing *cis*-regulatory bandwidth ($L= 250$ kb, Fig. 2D), we estimated the changes in *cis*-regulatory TE activities associated with each dox-induced transgene overexpression experiment (Additional file 8, Table S7). Dox-treatment alone and dox-induced GFP overexpression were not associated with any robust statistically significant change in *cis*-regulatory TE activity (Figs. 3A, S3A–B, Table S7) suggesting that neither the addition of doxycycline nor the metabolic cost entailed by strong transgene overexpression measurably altered the *cis*-regulatory activity of TE subfamilies. Interestingly, overexpression of the core pluripotency TF POU5F1 (also known as OCT4) was not associated with differential TE *cis*-regulatory activity (Fig. S3A–B), suggesting that overexpressing an already highly expressed gene, namely POU5F1, in a cellular context that largely relies on it, i.e., primed hESCs, may not necessarily alter TE-dependent *cis*-regulation. Together, these results suggest that TE-dependent *cis*-regulatory activities inferred from the remaining transgene overexpression experiments are not driven by technical factors inherent to the system used but induced by the overexpressed transgene itself.

To reveal how TE-dependent *cis*-regulation relies on TF/transgene overexpression in hESCs, we performed hierarchical clustering on the matrix containing the statistical strengths of the estimated *cis*-regulatory TE activities (Additional file 9, Fig. S3C). Stratifying subfamilies according to size and evolutionary age [5] did not reveal any discernible bias regarding the distribution of statistically significant *cis*-regulatory activities (Fig. S2C–D). Additionally, the directionality and statistical significance of TE-dependent *cis*-regulatory activities were robust to varying the *cis*-regulatory bandwidth *L* (Fig. S4, Table S7) and consistent across replicates when analyzed individually (Fig. S5). Overexpressed TFs of the same family tended to cluster together — e.g., NEUROD1, NEUROD2, NEUROG3, NEUROD4; PAX2, PAX5, PAX8; SNAI1, SNAI2, SNAI3; RUNX1, RUNX3; HES1, HEY1; LHX1, LHX5; GATA1, GATA2, GATA3 — whether considering effect size (Figs. 3A, S4) or statistical significance (Fig. S3C) of the estimated differences in TE-dependent *cis*-regulatory activity. This suggests that commonalities in gene expression [50] likely driven by shared DNA binding motifs [51] were partially mirrored by similar *cis*-regulatory TE activity patterns. Experiments where the core trophoblast TF CDX2 [52, 53] was overexpressed clustered away from all other experiments according to statistical significance (Additional file 9, Fig. S3C) but less so according to *cis*-regulatory activity estimates (Fig. 3A). This may reflect both the widespread rewiring of TE-dependent *cis*-regulation as primed hESCs differentiate towards trophectodermal cells [23] and a bias towards the detection of more differentially active *cis*-regulatory TE subfamilies in the CDX2 overexpression experiments due to a larger sample size compared to the other experiments (Fig. S5) [47].

The LTR7 subfamily clustered away from all other TE subfamilies (Additional file 9) and was statistically significantly less active in 186 out of 441 transgene overexpression experiments, making it the most frequently differentially active TE subfamily in this dataset (Fig. S2D). Among overexpressed transgenes tied to a decrease of LTR7-dependent *cis*-regulatory activity, we found multiple TFs involved in post-implantation developmental stages (Fig. 3A, Table S7), e.g., the meso/endodermal master TF GATA6 [54] and several homeobox-domain-containing TFs including PDX1 and RUNX1. LTR7 *cis*-regulatory activity also decreased upon overexpression of organ and tissue-specific TFs, e.g., NEUROD1, NEUROD2, NEUROD3, NEUROD4, MYT1, NR2E1, POU4F1, and POU4F3, all involved in the formation of the nervous lineage [55–59]. Overexpression of TBX5, a key TF in the developing heart [60] also decreased the *cis*-regulatory activity of LTR7. Lastly, overexpression of TFs involved in the development and maintenance of the placenta, e.g., CDX2, TEAD4 [61] also led to a decrease of LTR7 *cis*-regulatory activity. Overall, inducing TFs tied to development and differentiation dampened the pluripotency-specific activity of LTR7 elements.

In contrast, we found rare transgenes (9/441) whose overexpression in primed hESCs led to an increase in LTR7 *cis*-regulatory activity (Additional file 8, Fig. 3A, Table S7). Overexpressing KLFs collectively increased the *cis*-regulatory activity of LTR7Y elements in agreement with previous studies characterizing KLFs as inducers of LTR7Y enhancer activity in naïve hESCs [5]. Interestingly, induction of KLF5 — but not of KLF1, KLF2, and KLF4 — increased the *cis*-regulatory activity of LTR7, matching previously reported visual inspections which revealed that among these, only KLF5-overexpressing cells retained an ESC-like phenotype 72 h post-induction [50]. By leveraging a large compendium of homogeneously reprocessed ChIP-seq data [62], we confirmed that KLF4 binding was enriched at LTR7 and LTR7Y in various contexts related to primed hESCs [5, 63] (Fig. 3B). MYB (also known as c-MYB), a TF involved in the maintenance of self-renewal in stem cells of the intestinal crypt, the bone marrow, and the nervous system [64] as well as the formation of stem-like memory CD8 T cells [65], led to a marked increase in LTR7 *cis*-regulatory activity upon induction and displayed a modest enrichment in binding at related LTR7C integrants in monocytic-derived THP1 cells [66] (Fig. 3B). Thus, MYB overexpression may reinforce self-renewal in the GRN of hESCs, a process tied to an increase in LTR7 *cis*-regulatory activity. More provocatively, this hints at the possible involvement of a MYB-LTR7 axis in the maintenance of self-renewal and stemness in the adult hematopoietic system. Our analysis thus suggests that a limited set of TFs linked to development and stemness may rely upon the enhancer potential of the LTR7 subfamily to establish, regulate, and maintain these processes throughout development and adult life.

Other primate-specific TE subfamilies displayed partially overlapping patterns of *cis*-regulatory activity upon transgene overexpression. SVAs and LTR5-Hs were collectively activated by KLF4 and other KLFs (Fig. 3A, Table S7) and enriched for KLF4 binding in hESCs (Fig. 3B), consistent with previous work establishing the KLF4-dependent enhancer activity of these subfamilies in naïve hESCs [5]. Interestingly, the *cis*-regulatory activity of LTR5-Hs and SVAs also increased upon overexpression of TFAP2C and NR5A1, both of which polarize hESCs towards the naïve state [67, 68]. Overall, these results suggest that recently emerged TE subfamilies form functional collections of enhancer-like CREs during pre-gastrulation embryogenesis.

We then wondered whether the overexpression of transgenes necessary for embryonic development during and after gastrulation was associated with an increase in *cis*-regulatory activity in recently emerged TE subfamilies. Overexpression of the core meso/endodermal TF GATA6 as well as other GATA family members increased the *cis*-regulatory activity of SVAs and LTR5-Hs (Fig. 3A, Table S7), thereby resulting in the activation of a TE-dependent *cis*-regulatory network partially reminiscing that of naïve hESCs [5, 6]. This is surprising given that naïve hESCs resemble cells of the early blastocyst while GATA6 controls post-implantation developmental stages such as the formation of the mesoderm and the endoderm during gastrulation. Furthermore, overexpressing GATA family members increased the *cis*-regulatory activity of additional primate-specific TE subfamilies including the LTR5-Hs-related HERV-K subfamily LTR5B and the ERV1 subfamilies LTR6A, LTR6B, PRIMA4-LTR, MER4A1, MER4D, and MER4D1. Importantly, LTR6B displayed the largest and most statistically significant increase in TE-dependent *cis*-regulatory activity along stem cell to endoderm differentiation across two independent datasets (Fig. 3D, S3F) [69–71]. Moreover, GATA6 KO [69] reduced LTR6B *cis*-regulatory activity in differentiating endodermal cells, whereas GATA6 re-expression rescued it (Fig. 3D). Along the same lines, GATA2 deletion in hematopoietic progenitor cells [72] decreased the *cis*-regulatory activity of LTR5-Hs and LTR5B (Fig. S3D). Of note, GATA ChIP-seq peaks [62, 70] were strongly enriched at SVAs, LTR5-Hs, LTR5B, LTR6A, LTR6B, PRIMA4-LTR, MER4A1, MER4D, and MER4D1 integrants across primitive streak-derived [73] as well as mesendodermal [70], mesodermal [74] — including blood-derived [75–78] — and placental lineage [79] cells, with enriched binding at SVAs, LTR5-Hs, LTR5B, LTR6A, and LTR6B extending to the endodermal lineage [80, 81] (Fig. 3B). Together, these patterns of binding suggest that the changes in *cis*-regulatory activity observed upon GATA overexpression in hESCs and meso/endodermal differentiation result from the direct binding of GATA family members to primate-specific TE subfamilies. Interestingly, overexpressing EOMES, another regulator of germ layer formation and mesoendodermal differentiation [82], markedly increased the *cis*-regulatory activity of LTR6B elements (Fig. 3A), at which it also displayed enriched binding in hESC-derived mesendodermal cells [70] (Fig. 3B). Moreover, overexpression of SOX17, an additional regulator of endodermal differentiation [83], increased the *cis*-regulatory activity of LTR5-Hs, SVA-C, and LTR6B (Fig. 3A), while SOX17 binding was strongly enriched at SVAs, LTR7, and MER4A1 in germ cell-derived Tcam-2 cancer cells [84] (Fig. 3B), which share some phenotypic features with primordial germ cells (PGCs).

Evidence linking transcription during post-gastrulation embryogenesis with primate-specific TE-mediated *cis*-regulation extended beyond SOX17 to TFAP2C and SOX15, which both display elevated expression in the PGC lineage [85]. Specifically, overexpression of SOX15 in hESCs markedly increased the *cis*-regulatory activity of LTR5-Hs (Fig. 3A, Table S7), the latter exhibiting the largest statistically significant increase in *cis*-regulatory activity in human PGC-like cells (hPGCLCs) compared with cognate hESC-derived somatic cells (Fig. 3C) [86]. Knocking out SOX15 in hPGCLCs led to a drop in LTR5-Hs *cis*-regulatory activity across two biological replicates (Figs. 3C, S3E), while SOX15 binding was strongly enriched at LTR5-Hs in hPGCLCs (Fig. 3B). Additionally, inducing TFAP2C in hESCs increased the *cis*-regulatory activity of LTR5-Hs and SVAs (Fig. 3A), both of which displayed a considerable enrichment in TFAP2C binding in hPGCLCs [87] and, interestingly, in cells of the ectoderm lineage [88, 89] (Fig. 3B). In summary, evolutionarily recent and pre-implantation specific TE subfamilies form sets of CREs that regulate the expression of protein-coding genes in *cis* well past the epiblast stage, including during and after gastrulation, as evidenced firstly by increased *cis*-regulatory activity following germ layer-specific TF overexpression in hESCs, secondly by enriched TF binding in cells derived from the corresponding germ layers and thirdly by substantial stage-specific increases in *cis*-regulatory activity which were reverted upon germ layer-specific TF KO.

Older TE subfamilies that emerged prior to the speciation of primates also contribute to GRNs by donating CREs. Despite having spread before the speciation of amniotes hundreds of millions of years ago, AmnSINE1 elements are retained in the genomes of extant amniotes including humans and mice [90], and some AmnSINE1 elements were found to exert long-range enhancer effects on genes controlling brain development [91]. We observed that in primed hESCs, overexpression of several homeobox domain-containing TFs, e.g., RUNX1, a regulator of hematopoietic ontogeny [92], and PDX1, involved in pancreatic development [93], was associated with an increased AmnSINE1 *cis*-regulatory activity (Fig. 3A, Table S7). Interestingly, AmnSINE1 elements are enriched within active enhancers in epigenomes derived from fetal human cell lines [21]. Lastly, MER135, an ancient subfamily of currently unidentified origin [94] showed increased *cis*-regulatory activity upon overexpression of homeobox domain-containing TFs in primed hESCs (Fig. 3A). More generally, these results hint that ancient TE subfamilies may retain their *cis*-regulatory potential at the subfamily level in extant species despite having colonized the genome of an evolutionarily distant common ancestor.

### *Cis*-regulatory activities are more pronounced at epigenetically active TEs

**Fig. 4:**
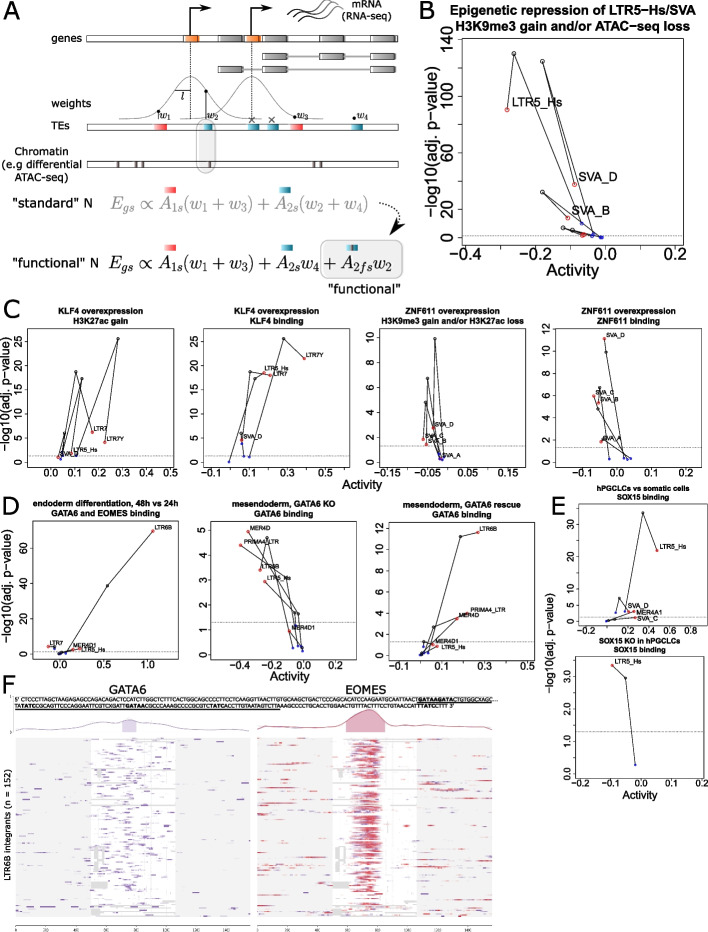
*Cis*-regulatory activities are more pronounced at epigenetically active TEs. **A** Overview of the procedure whereby TE subfamilies are split between so-called “functional” and “non-functional” fractions based on additional evidence, e.g., differential chromatin accessibility. The regulatory susceptibility scores tying TE subfamilies to protein-coding genes are distributed between the functional and non-functional fractions of each TE subfamily, leading to an experiment-specific column-wise expansion of *N*. Concretely, functional and non-functional fractions of TE subfamilies are treated as independent TE subfamilies in the subsequent *cis*-regulatory activity estimation process. **B** Estimated differences in *cis*-regulatory activity for the functional (in red) and non-functional fractions (in blue) of LTR5-Hs and SVA subfamilies under CRISPRi-mediated epigenetic repression in naïve hESCs [33]. The *cis*-regulatory activities for the unsplit subfamilies were estimated in a separated iteration of *craTEs*, using the standard distance-weighted *N* matrix ($L=250$ kb), and are shown in black. The dotted line represents the significance threshold of BH-adjusted $p.val = 0.05$. Note that even though only selected subfamilies are plotted for clarity, all TE subfamilies were included in the fitting process. **C** Estimated differences *cis*-regulatory activity for the functional and non-functional fractions of selected TE subfamilies according to definitions of the functional state that are either based on differential chromatin states (1st and 3rd panels from the left) or differential TF binding (2nd and 4th panels from the left) at integrants [33]. **D** Estimated differences in *cis*-regulatory activities for the functional (bound by both GATA6 and EOMES [70]) vs. non-functional fractions of selected TE subfamilies during hESC-derived endoderm differentiation, 48 h vs. 24 h, $n=3$ [70] (left), functional (GATA6-bound [70]) vs. non-functional fractions of selected TE subfamilies upon GATA6 KO in iPSC-derived mesendoderm, $n=2$ [69] (center) and GATA6 rescue in GATA6 KO iPSC-derived mesendoderm, $n=2$ (right). **E** Estimated differences in *cis*-regulatory activity for the functional (SOX15-bound) vs. non-functional fractions of selected TE subfamilies between DP hESC-derived hPGCLCs and DN somatic cells, day 6, $n=2$ [86] (top) and SOX15 KO in DP hESC-derived hPGCLCs (bottom). **F** Multiple sequence alignment (MSA) of all 152 LTR6B integrants considered by *craTEs* (central white rectangle). Gray patches within the central white rectangle indicate gaps. Sequences at loci found in gray rectangles flanking the MSA region are shown for convenience and were not aligned. The intensity of GATA6 (left, $n=1$) and EOMES (right, $n=2$) ChIP-seq signal is indicated at the corresponding genomic loci. The fraction of sequences adorned with ChIP-seq signal for each position is shown on top. Consensus sequences found underneath high-density ChIP-seq signal regions ($>\frac{1}{3}$ of sequences overlapping ChIP-seq reads) with the highest density of GATA6 (underlined), resp. EOMES (entire consensus) signals are reported, with GATA consensus DNA-binding sites in bold

We showed that *craTEs* agnostically uncovers SVAs and LTR5-Hs as the subfamilies with the most statistically significant and strongest loss of *cis*-regulatory activity upon CRISPRi-mediated epigenetic repression in naïve hESCs (Fig. 1). However, it is highly likely that only a fraction of all integrants constituting a subfamily truly exert *cis*-regulatory effects. For example, integrants found within dynamic chromatin regions may be more differentially active than integrants located in stable chromatin regions. To empirically verify this hypothesis, we leveraged the epigenomic profiles matched to the LTR5-Hs/SVA CRISPRi RNA-seq dataset [33] and labeled the following integrants as “functional”: those overlapping with genomic coordinates where loss of chromatin accessibility (ATAC-seq) or gain of the repressive histone mark H3K9me3 (ChIP-seq) were detected upon epigenetic repression of SVAs and LTR5-Hs. Conversely and by complementarity, we considered all other integrants from these subfamilies as “non-functional.” We then expanded the weighted susceptibility matrix *N* ($L = 250$ kb) column-wise by splitting TE subfamilies into complementary functional and non-functional integrant subsets (Fig. 4A). Finally, we used *craTEs* to jointly estimate the differences in *cis*-regulatory activity for functional and non-functional subsets of TE subfamilies upon epigenetic repression of LTR5-Hs and SVAs in naïve hESCs (Fig. 4B). Functional subsets of SVAs and LTR5-Hs subfamilies displayed greater decreases in *cis*-regulatory activity upon epigenetic repression than complementary non-functional subsets. In addition, the estimated decrease in *cis*-regulatory activity was more pronounced for the subset of functional LTR5-Hs than that estimated for the corresponding unsplit subfamily. Of note, functional LTR5-Hs and SVA integrants tended to show slightly lower mappability scores [95] than non-functional integrants (Fig. S7A), raising the concern that difficulties in ChIP-seq and/or ATAC-seq read assignment at repeats [96] may drive functional versus non-functional integrant calling, thereby biasing TE subfamily *cis*-regulatory activity estimates. However, low mappability functional LTR5-Hs/SVA integrants still exhibited larger and more statistically significant decreases in estimated *cis*-regulatory activity upon CRISPRi than their low mappability non-functional counterparts (Fig. S7A).

Next, we leveraged the epigenomics-informed adaptation of *craTEs* to test whether differences in TF binding or histone marks could single out integrants with detectable changes in *cis*-regulatory activity in the context of TF overexpression experiments. To this end, we completed the matched transcriptomics and histone profiles available for KLF4 and ZNF611 overexpression in hESCs [33] by generating ChIP-seq profiles against KLF4 and ZNF611. We used *craTEs* to estimate differences in *cis*-regulatory activity for functional integrant subsets defined according to differences in histone marks or TF binding and focused on the main *cis*-regulatory TE subfamilies identified under KLF4 and ZN611 overexpression in hESCs, namely LTR7, LTR5-Hs, and SVAs (Fig. 4C). For both KLF4 and ZN611 overexpression experiments, histone mark-defined functional subsets had greater *cis*-regulatory activity than non-functional subsets, except for SVA-D under KLF4 overexpression, as well as SVA-A under ZNF611 overexpression. However, histone mark-defined functional subsets generally displayed only modest increases in *cis*-regulatory activity over unsplit subfamilies, at the cost of a marked decrease in statistical significance. In contrast, TF-bound-defined functional integrants displayed increased *cis*-regulatory activity compared with the unsplit subfamily for all subfamilies except SVA-D under KLF4 overexpression, with increased significance in most cases, including when matching integrants for mappability prior to functional versus non-functional subfamily splitting (Fig. S7B). Turning to cellular contexts featuring endogenous TF expression levels, we compared the usefulness of mesendodermal GATA6, EOMES, and H3K27ac ChIP-seq peaks [70] for delineating the most active fraction of TE subfamilies in terms of *cis*-regulation. GATA6 and EOMES binding, whether considered individually or in combination, proved remarkably informative for discriminating *cis*-regulatory LTR6B integrants from their inactive counterparts during endoderm differentiation (Figs. 4D, S6A), and conversely aptly accounted for the decrease, resp. increase, in LTR6B, PRIMA4-LTR, MER4D, MER4D1, and LTR5-Hs observed upon GATA6 KO, resp. rescue, in iPSC-derived endodermal cells [69] (Fig. 4D). In contrast, H3K27ac peaks failed to single out *cis*-regulatory LTR6B integrants in this context. Indeed LTR6B integrants devoid of the canonical enhancer histone mark exerted a more statistically significant *cis*-regulatory activity than LTR6B integrants overlapping H3K27ac peaks (Fig. S6A). Similarly, SOX15 CUT &TAG peaks [86] pinpointed LTR5-Hs integrants displaying increased *cis*-regulatory activity in hPGCLCs versus cognate somatic cells, a trend that was inverted upon SOX15 KO in hPGCLCs (Fig. 4E). As implied by differing patterns in statistical significance at functional versus non-functional LTR5-Hs integrants, SOX15 binding better isolated *cis*-regulatory active LTR5-Hs than chromatin accessibility as assessed by ATAC-seq, although both epigenomic signals were in general agreement (Fig. S6B). Importantly, we did not observe any noticeable difference in mappability between functional and non-functional integrants in ZNF611 overexpressing cell, endodermal cell [70], or hPGCLC-derived [86] epigenomic data (Fig. S7C–E). Thus, TF binding appears to better single out bona fide *cis*-regulatory integrants than changes in histone marks.

As both EOMES and GATA6 binding were associated with increased LTR6B, LTR6A, LTR5-Hs, MER4D, MER4D1, and PRIMA4-LTR *cis*-regulatory activity in the differentiating endoderm (Fig. 3B), we performed multiple sequence alignment (MSA) of subfamily-restricted ChIP-seq peaks in search of EOMES and GATA6 TF binding motifs. MSA of GATA6 and EOMES peaks at LTR6A and LTR6B revealed consensus sequences containing several canonical GATA binding sites (Fig. 4F, Additional file 10). Fittingly, the DNA sequence of most LTR6B integrants adorned with GATA6/EOMES peaks contained recognizable GATA6 DNA binding motifs (Fig. S6C), as assessed through motif search [97]. Of note, MSA of EOMES peaks revealed additional GATA6 motifs in per-subfamily consensus sequences at LTR5-Hs, MER4D, MER4D1, and PRIMA4-LTR (Additional file 10), while we failed to call consensus sequences from GATA6 ChIP-seq peaks at these subfamilies. This may stem from differences in ChIP-seq protocols and/or quality. Intriguingly, MSA of EOMES-bound TE regions did not reveal any canonical EOMES DNA binding site, a finding supported by the absence of EOMES motifs at LTR6B integrants subjected to motif search (Fig. S6C). This suggests that GATA6 may first bind at primate-specific TE subfamilies in the differentiating endoderm, to then promote EOMES recruitment at these loci. Accordingly, LTR6B and MER4D1 integrants carrying GATA6 DNA-binding motifs were more *cis*-regulatory than those devoid of the motif in the differentiating endoderm, whether considering statistical significance or effect size (Fig. S6D). The presence of a GATA6 motif also separated truly *cis*-regulatory from non-*cis*-regulatory LTR6B integrants upon GATA6 KO/rescue in the differentiating endoderm but proved less discriminant than ChIP-seq-derived GATA6/EOMES binding at LTR6A, LTR5-Hs, MER4D, and MER4D1 (Fig. S6D). Overall, the agreement between GATA6/EOMES, resp. SOX15 binding, and primate-specific TE-mediated *cis*-regulation in endodermal fetal cells, resp. hPGCLCs, suggests that recently evolved TEs spread functional *cis*-regulatory platforms at which core TFs controlling post-gastrulation embryogenesis directly bind, in turn affecting protein-coding gene expression.

## Discussion

The notion that some TE-derived sequences behave as bona fide CREs is supported by an ever-growing number of reports mostly relying on genome-wide profiles of promoter- or enhancer-specific histone marks. However, whether that biochemical activity should be interpreted as evidence for an evolutionary process fostering the emergence of collections of CREs to the benefit of the host, or instead as a byproduct of the so-called “selfish” tendency of TEs for genome invasion is subject to debate [10, 11, 15]. Still, if TEs truly spread functional CREs that become co-opted by the host through natural selection, one should at least be able to capture their effect on gene expression by modeling TE-dependent *cis*-regulation from basic gene regulation principles. Thus, we formulated *craTEs*, a system where differences in TE subfamily *cis*-regulatory activities are estimated in a single step from protein-coding gene expression. Using RNA-seq data derived from thoroughly characterized cases of TE-dependent *cis*-regulation, we showed that *craTEs* correctly identifies differentially active *cis*-regulatory TE subfamilies. Moreover, we could refine activity estimations by incorporating context-matched epigenomics data — e.g., TF binding or chromatin marks — into *craTEs*, highlighting that protein-coding gene expression and chromatin states at TEs fruitfully complement each other for uncovering TE-dependent *cis*-regulation. Crucially, *craTEs* does not rely on TE-derived reads and is thus well-suited for the post-hoc analysis of standard RNA-seq count tables that did not take TE transcripts into account during feature quantification. In addition, *craTEs* was able to identify *cis*-regulatory TE subfamilies (SVAs) in RNA-seq datasets where no difference in transcriptional activity for these TE subfamilies was previously detected [33]. These results suggest that TE subfamilies form at least partially consistent sets of CREs modulating gene expression in a coordinated fashion genome-wide and more generally that TEs spread highly resembling and functional *cis*-regulatory sites thereby supplying the raw materials critical to the evolution of coordinated gene regulation. Note that *craTEs* cannot discriminate between waves of transposition whereby TE subfamilies spread sequences poised to acquire TFBS by gradual mutations from those whereby extant TFBS were intrinsic constituents of *de novo *transposed integrants. The former may be most relevant for older TE subfamilies, e.g., AmnSINE1, MER121, and MER135 [98]. Whereas *craTEs* relies on sequence similarity as encoded in the TE models used by Repeatmasker, relatedness across subfamilies is currently not modeled: each subfamily is considered as phylogenetically equidistant to all others which may hamper sensitivity. One possible extension to *craTEs* entails encoding sequence similarity across subfamilies as a nearest-neighbor graph to constrain closely related TE subfamilies to receive similar *cis*-regulatory activities via penalized regression, e.g., the fused lasso [99]. We noticed that *craTEs* explains a fraction of the variation in gene expression ranging from approx. 2% to 12%, which is comparable to the proportion of variance in gene expression explained by previously published linear models of gene regulation based on putative TF binding sites at core promoters [29]. In both cases, the low proportion of variance captured by the linear models is still sufficient for identifying statistically significant and relevant regulatory mechanisms from transcriptomic data alone. However, while the true mathematical function underlying the regulatory mechanism at play is most likely non-linear [29], the linear model proposed in this work is still useful. Inferred activity coefficients are interpretable and well-established statistical tests exist to determine whether differences in *cis*-regulatory activities statistically significantly deviate from zero. What is perhaps more impressive is that, in the context of this study at least, TE-dependent *cis*-regulation accounts for a fraction of the variation in gene expression comparable to that inferred from models of gene regulation based on the TFBS repertoire of core promoters. This observation underlines that TEs should not be ignored when attempting to delineate the regulatory programs orchestrating biological processes, in particular during embryogenesis.

We have also shown that *craTEs* identifies relevant *cis*-regulatory TE subfamilies with superior power compared to enrichment approaches based on differential expression analysis. In addition, *craTEs* readily identifies TE-dependent *cis*-regulatory changes in experiments limited to a single pair of samples, e.g., treatment versus control, whereas performing differential expression analysis requires at least a replicate in one of the conditions, i.e., three samples. This difference likely stems from how gene expression values are modeled in either method. DE methods model the distribution of gene expression values across conditions independently for each gene, although current methods now leverage information borrowing techniques to share information across genes within samples [46]. In effect, DE methods perform one statistical test for each gene, resulting in tens of thousands of tests where the false discovery rate has to be controlled. Thus, any coordinated but mild difference in expression between co-regulated sets of genes is lost and cannot be used in the subsequent enrichment test. In contrast, *craTEs* leverages information across hundreds to thousands of genes to estimate the *cis*-regulatory activity of each TE subfamily in a single step. We leveraged epigenomic data — namely ATAC-seq and H3K9me3 ChIP-seq — to supplement gRNA complementarity in defining a ground truth set of differentially *cis*-regulatory TE subfamilies under LTR5-Hs/SVA CRISPRi in naïve hESCs. *craTEs* performed remarkably similarly to enrichment approaches based on epigenomic data, picking up subtle differences between CRISPRi gRNAs. Indeed, while LTR5-Hs and SVA A-D were enriched for epigenomic signals indicative of heterochromatin under both g#1 and g#2, SVA E/F only did so under g#2. One putative limitation of *craTEs* compared with enrichment approaches stems from the fact that it estimates exactly one activity coefficient per TE subfamily. Consequently, *craTEs* may fail to recover *cis*-regulatory TE subfamilies encompassing integrants exerting antagonistic *cis*-regulatory effects. In that case, enrichment approaches may benefit from treating increased and decreased expression and/or epigenomic signal separately, though this would likely require large sample sizes.

Next, we empirically determined that the typical range until which *cis*-regulatory TEs regulate their target promoters is 500 kb. To this end, we applied a cross-validation procedure to a large-scale primed hESCs perturbation dataset to select the *cis*-regulatory distance that minimized the error between true and predicted gene expression values as estimated by *craTEs*. To our knowledge, this is the first attempt aimed at quantitatively estimating such distance by aggregating transcriptomic data derived from hundreds of experimental perturbations. Thus, TE subfamilies exert *cis*-regulatory influences up to distances compatible with those typically separating enhancers from their target promoters, consistent with the notion that many TEeRS are, in fact, bona fide enhancers. Additionally, constraining TE-promoter *cis*-regulatory weights to TAD boundaries yielded similar predictions to TAD-agnostic distance weighting, though generally not ameliorating gene expression prediction.

We further characterized the landscape of TF overexpression-induced TE-dependent *cis*-regulatory changes. TFs poised towards the GRN of the naïve hESC state, namely TFAP2C, KLFs, and NR5A1, collectively bound to and increased the *cis*-regulatory activity of LTR5-Hs, SVA, and LTR7Y subfamilies, which function as KLF4-responsive enhancers in naïve hESCs. Whether these newly identified inducers of evolutionarily young and *cis*-regulatory TE subfamilies mediate their effect via direct binding or secondary transcriptional changes will require further work. Still, these results underline the importance of LTR5-Hs, SVA, and LTR7Y subfamilies in the GRN of naïve pluripotency. Of note, whereas KLF5 overexpression was accompanied by increased LTR7 *cis*-regulatory activity, overexpression of KLF1, KLF2, and KLF4 was associated with a decrease in LTR7 *cis*-regulatory activity. This apparent discrepancy with the established role of LTR7 as KLF4-responsive enhancers [5, 100, 101] may stem from differences in overexpression levels or hESC backgrounds and may be explained by the dual involvement of KLF4 in both naïve pluripotency [5] and terminal differentiation in mesodermal [102] as well as endodermal [103] lineages. While the *cis*-regulatory activity of young TE subfamilies in pre-implantation embryogenesis is increasingly being recognized [98], the landscape of TE-dependent *cis*-regulation at later stages of human embryogenesis is still ill-defined. In the present study, we observed that inducing key regulators of gastrulation, germ layer/placental commitment and PGC differentiation — including GATA2, GATA6, EOMES, and SOX15 — in hESCs increased the *cis*-regulatory activity of LTR5-Hs and SVA subfamilies together with other primate-specific TE subfamilies such as LTR5B, LTR6B, MER4A1, MER4D/D1, and PRIMA4-LTR. Importantly, the binding of these TFs was enriched at the TE subfamilies they activated across various models of embryogenesis and differentiated tissues. TF binding as assessed by context-matched ChIP-seq/CUT &TAG profiles aptly discriminated truly *cis*-regulatory from inactive integrants during endoderm and hPGCLC differentiation, as well as upon KO/rescue of the corresponding TFs. Of note, we reported in a related manuscript [40] that LTR5B, LTR5-Hs, LTR6A, and LTR6B integrants are highly accessible in endodermal and mesodermal human fetal cells, though more rarely in ectodermal cells and that selected LTR6B integrants serve as enhancers for genes encoding key mesendodermal regulators. Finally, MSA of GATA6 and EOMES-bound LTR6B regions in the differentiating endoderm revealed a GATA-rich consensus sequence, and GATA6 DNA-binding motifs uncovered through motif search recapitulated the functional versus non-functional dichotomy defined using ChIP-seq data for predicting gene expression. Thus, the *cis*-regulatory role played by primate-restricted TEs during pre-implantation embryogenesis appears maintained — if not reactivated — by developmental stage-specific TFs during subsequent steps of embryogenesis.

Lastly, we leveraged epigenomics data to test whether changes in chromatin states and evidence for direct TF binding could single out *cis*-regulatory integrants from non-*cis*-regulatory integrants within TE subfamilies. Surprisingly, we found that across various experimental systems entailing TF overexpression, TF KO, and endogenous TF expression, TF binding was better able to enrich for *cis*-regulatory integrants than changes in histone marks and/or chromatin accessibility, though how TF binding compares with context-matched chromatin states as defined using combinations of histone marks [104] remains to be seen. In the case of KLF4 overexpression, it is possible that the partial activation of compensatory TE-silencing mechanisms caused a divergence between chromatin and TF binding-derived *cis*-regulatory signals. Lastly, *craTEs* may benefit from the incorporation of STARR-ChIP-seq data, though whether fragment length and genome coverage shall prove appropriate for studying TE-mediated *cis*-regulation will have to be assessed.

## Conclusion

That a simple mathematical model based on TE-promoter distances and the expression of protein-coding genes can infer TE-mediated *cis*-regulatory activities illustrates that as TEs spread, they rewire nearby protein-coding genes into a web of regulatory dependencies which can be simultaneously fine-tuned by only a handful of transcriptional regulators. Furthermore, these recently emerged GRN components appear to regulate not only early embryogenesis, but also more advanced stages of development. For such vital and highly conserved events, the resulting speciation is only mechanistic owing to selective pressures. In those cases, the TE-dependent and species-specific CRE turnover is likely to result in equivalent phenotypic adaptations across species, as reproductive/survival stakes leave little room for organismal novelty. However, in situations allowing for more phenotypic diversification, for instance in the brain, the rapidly evolving TE-based *cis*-acting regulome likely contributes to the emergence of new traits.

## Methods

### Cell culture

H1 male and WIBR3 female human embryonic stem cells were provided by the Krause and Jaenisch lab, respectively, and both tested negative in the Mycoplasmacheck from eurofins Genomics upon receipt and throughout the study.

#### Treatment protocol 

Primed H1 were transduced with GFP or KLF4-containing lentiviral vectors and split after 48 h then selected using blasticydin for the 3 following days. Naïve WIBR3dPE hESC cells in KN/2iL media were transduced with GFP or ZNF611-containing lentiviral vectors, split after 96h, then selected for a couple of passages with blasticydin on irradiated Mouse Embryonic Blasticidin-resistant (MMMbz).

#### Growth protocol

Conventional (primed) human ESC lines were maintained in mTSER for H1 (male) on Matrigel, for WIBR3 (female) on irradiated inactivated mouse embryonic fibroblast (MEF) feeders in human ESC medium (hESM) and passaged with collagenase and dispase, followed by sequential sedimentation steps in hESM to remove single cells while naïve ES cells and primed H1 were passaged by Accutase in single cells. hES media composition: DMEM/F12 supplemented with 15% fetal bovine serum, 5% KnockOut Serum Replacement, 2 mM L-glutamine, 1% nonessential amino acids, 1% penicillin-streptomycin (Lonza), 0.1 mM $\upbeta$-mercaptoethanol and 4 ng/ml FGF2. Naïve media composition: 500 mL of medium was generated by including 240 mL DMEM/F12, 240 mL neurobasal, 5 mL N2 supplement, 10 mL B27 supplement, 2 mM L-glutamine, 1% nonessential amino acids, 0.1 mM $\upbeta$-mercaptoethanol, 1% penicillin-streptomycin, 50 µg/ml BSA. In addition for KN/2i media: PD0325901 (1 µM), CHIR99021 (1 µM), 20 ng/ml hLIF, and doxycycline (2 µg/ml).

### ChIP-seq

Cells were cross-linked for 10 min at room temperature by the addition of one-tenth of the volume of 11% formaldehyde solution to the PBS followed by quenching with glycine. Cells were washed twice with PBS, then the supernatant was aspirated and the cell pellet was conserved in − $80^{\circ }$C. Pellets were lysed, resuspended in 1mL of LB1 on ice for 10 min (50 mM HEPES-KOH pH 7.4, 140 mM NaCl, 1 mM EDTA, 0.5 mM EGTA, 10% glycerol, 0.5% NP40, 0.25% Tx100, protease inhibitors), then after centrifugation resuspend in LB2 on ice for 10 min (10 mM Tris pH 8.0, 200 mM NaCl, 1 mM EDTA, 0.5 mM EGTA and protease inhibitors). After centrifugation, resuspend in LB3 (10 mM Tris pH 8.0, 200 mM NaCl, 1 mM EDTA, 0.5 mM EGTA, 0.1% NaDOC, 0.1% SDS and protease inhibitors) for histone marks and SDS shearing buffer (10 mM Tris pH8, EDTA 1mM, SDS 0.15% and protease inhibitors) for transcription factor and sonicated (Covaris settings: 5% duty, 200 cycle, 140 PIP, 20 min), yielding genomic DNA fragments with a bulk size of 100–300 bp. Coating of the beads with the specific antibody and carried out during the day at $4^{\circ }$C, then chromatin was added overnight at $4^{\circ }$C for histone marks while antibody for transcription factor is incubated with chromatin first with 1% Triton and 150 mM NaCl. Subsequently, washes were performed with 2× Low Salt Wash Buffer (10 mM Tris pH 8, 1 mM EDTA, 150 mM NaCl, 0.15% SDS), 1× High Salt Wash Buffer (10 mM Tris pH 8, 1 mM EDTA, 500 mM NaCl, 0.15% SDS), 1× LiCl buffer (10 mM Tris pH 8, 1 mM EDTA, 0.5 mM EGTA, 250 mM LiCl, 1% NP40, 1% NaDOC) and 1 with TE buffer. The final DNA was purified with the Qiagen Elute Column. Up to 10 ng of ChIPed DNA or input DNA (Input) were prepared for sequencing. Library was quality-checked by a DNA high-sensitivity chip (Agilent). Quality-controlled samples were then quantified by picogreen (Qubit 2.0 Fluorometer, Invitrogen). Cluster amplification and following sequencing steps strictly followed the Illumina standard protocol. Libraries were ligated with Illumina adaptors. Sequenced reads were demultiplexed to attribute each read to a DNA sample and then aligned to reference human genome hg19 with bowtie2 [105]. Peaks were called on mapped data using MACS2 [106]. Differential analysis between conditions has been performed with VOOM [107] using unique reads (filter for MAPQ <10), counted on the union of all peaks of the same experiment. Samples were normalized for sequencing depth using the counts on the union peaks as library size and using the TMM method [45] as it is implemented in the limma package of Bioconductor.

### ATAC-seq

ATAC-seq was performed as previously described [108] on primed WIRB3 and WIBR3dPE; naive WIBR3 and WIBR3dPE in 4iLA and KN/2iL media respectively; and in WIBR3dPE in KN/2iL media upon dCAS9-KRAB overexpression containing or not a guide RNA targeting SVA/LTR5Hs. Libraries were made using Nextera DNA Library Prep Kit (Illumina #FC-121-1030). ATAC-seq and DNase-seq reads were mapped to the human (hg19) genome using bowtie2 [105]. Mitochondrial reads were removed. Then accessible sites were called using MACS2 [106], only peaks with a score higher than 5 (−log10 *p* value) were kept. Then differential analysis between conditions was done using unique reads (filter for MAPQ <10), counted on the union of all peaks of the same experiment.

### RNA-seq analysis

#### Mapping

Reads were mapped to the human (hg19) genome using hisat2 [109] with parameters hisat2 -k 5 –seed 42 -p 7.

#### Summarization

Counts on genes and TEs were generated using featureCounts [110]. To avoid read assignation ambiguity between genes and TEs, a gtf file containing both was provided to featureCounts. For repetitive sequences, an in-house curated version of the Repbase database was used (fragmented EREs belonging to the same subfamily were merged). Only uniquely mapped reads were used for counting on genes and TEs. Finally, features that did not have at least one sample with 20 reads were discarded from the analysis. Only features corresponding to protein-coding genes were kept, except when quantifying SVA-derived transcription for Fig. S1A. Gene expression values pertaining to endoderm differentiation (48 h vs 24 h) [70] were obtained from GEO at accession number GSE213394. Gene expression values pertaining to hPGCLC differentiation with and without SOX15 KO [86] were retrieved using recount3 [111] and gene symbols were converted from genome assemblies hg38 to hg19 using ensembl Biomart [112].

#### Normalization

For input into *craTEs*, raw counts were transformed to transcripts per millions (TPM). A pseudocount equal to the fifth percentile of non-zero counts in the sample was added to each raw count before transformation to TPM and subsequent $\log _2$ transformation. For recount3-retrieved expression values, raw counts were used for filtering and the pre-computed TPM values were used.

### ChIP-seq enrichment at TE integrants

ChIP-seq binding locations from published datasets were extracted from ChIP-Atlas [62], except for the Wang et al. hPGCLCs datasets [86] for which we downloaded .narrowPeak files directly from GEO at accession number GSE143345 and the Luo et al. endodermal differentiation datasets [70] which were processed from fastq files as described above. Enrichment analysis over TE subfamilies was performed with HOMER software v4.10.4 [113], except for the Wang et al. [86] and Luo et al. [70] datasets, for which we used pyTEnrich available at URL https://github.com/alexdray86/pyTEnrich as previously described [114, 115]. To build Fig. 3B, we recovered the three top statistically significant enrichments for each selected pair of TE-TF — excluding WNT3A — highlighted in Fig. 3A. Enrichment values with *p*-val >1e−10 were filtered out. Cell type and germ layer assignments were hand curated by examining the original publications, retrieved from the SRA run numbers. When applicable, we excluded enrichment values derived from perturbation experiments — e.g., knock-down of a particular gene — and kept control samples instead. We excluded an H3K4me1 ChIP-seq sample that was erroneously labeled as a GATA1 ChIP-seq sample in ChIP-Atlas.

### Differential expression analysis-based *cis*-regulatory TE subfamily detection

DE analysis was performed using edgeR [45]. Starting from raw counts restricted to protein-coding genes, we performed library size normalization with the trimmed mean of *M*-values (TMM) normalization method [116]. We assumed that TMM-normalized counts follow a negative binomial distribution and estimated per-gene dispersions using the estimateDisp function from edgeR. We tested for differential expression using Fisher’s exact test as implemented in the function exactTest from edgeR. We either considered DE genes as those with Benjamini-Hochberg adjusted *p* values <0.05 (stringent DE calling), or those with *p* values < 0.05 (lenient DE calling). Next, using the hypergeometric distribution, we computed for each TE subfamily the probability of finding more DE genes within *cis*-regulatory distance of its integrants than what was observed [5, 44]. We performed this last step separately for upregulated and downregulated genes. Finally, we gathered the results obtained for up/downregulated genes into a single table and accounted for multiple testing using the Benjamini-Hochberg procedure [41]. We assessed how *craTEs* compared versus the DE enrichment approach by measuring their respective abilities to recover a ground truth set of *cis*-regulatory TE subfamilies in each of both LTR5-Hs/SVA CRISPRi experiments [33]. For each biological replicate, we defined the ground truth set using two criteria: (1) complementarity with the gRNA used in the corresponding CRISPRi experiment and (2) increased heterochromatin marks and/or decreased chromatin accessibility upon treatment with CRISPRi. The resulting ground truth sets were: [g#1: {LTR5-Hs, SVA-A, SVA-B, SVA-C, SVA-D}]. [g#2: {LTR5-Hs, SVA-A, SVA-B, SVA-C, SVA-D, SVA-E, SVA-F}]. As considering TE subfamilies as “sufficiently” *cis*-regulatory depends upon statistical significance and/or effect size thresholds, we used AUCs to systematically compare *craTEs* with competing approaches in the task of recovering truly *cis*-regulatory TE subfamilies. We used 1-(BH adj. *p*-values) as the probability of being classified as *cis*-regulatory. The AUC takes values between 0.5 and 1 and can be interpreted as the probability of having correctly ordered observations between classes such as to separate observations across both classes perfectly. An advantage of the AUC is that it allows for a detailed study of the relationship between sensitivity and specificity as the threshold for classification varies. Here, a perfect AUC = 1 would be reached in cases where ranking the adj. *p*-values yielded by *craTEs* ranks all TE subfamilies found in the ground truth as those with the most statistically significant changes in *cis*-regulatory activity.

### *Cis*-regulatory activity estimation for TE subfamilies (*craTEs*)

The *craTEs* model, available as an R package at URL https://github.com/pulvercyril/crates, was adapted from the motif activity response analysis (MARA) model of gene regulation [29]. Let *E* be the matrix of gene expression, with *P* protein coding genes as rows, and *S* samples as columns. $E_{ps}$ is the logged TPM expression value for gene *p* in sample *s*. Let *N* be the predictor/feature matrix with *P* protein coding genes as rows and *M* TE subfamilies as columns. $N_{pm}$ is regulatory susceptibility [27] of protein-coding gene *p* to TE subfamily *m*, and in the absence of weighting procedure is computed as the number of times an integrant belonging to TE subfamily *m* is found in the vicinity of *p*. Let *A* be the matrix of *cis*-regulatory TE subfamily activities, with *M* TE subfamilies as rows and *S* samples as columns. $A_{ms}$ is the *cis*-regulatory activity of TE subfamily *M* in sample *S*. $A_{ms}$ can be seen as follows: if a TE integrant from subfamily *m* is inserted in the vicinity of gene *p*, the expression of gene *p* increases by the value $A_{ms}$. Then, the expression $E_{ps}$ of gene *p* in sample *s* is given by:

3 $$\begin{aligned} E_{ps} = c_p + d_s + \sum _{m} N_{pm} A_{ms} + \epsilon \end{aligned}$$

where $c_p$ is a gene-specific constant representing basal transcription and $d_s$ is a sample-specific constant that models sample-specific batch effects such as PCR amplification biases. The model across samples and genes can be written as:

4 $$\begin{aligned} \left[ \begin{array}{ccc} E_{1 s} &{} ... &{} E_{1 S}\\ . &{} . &{} .\\ E_{P s} &{} ... &{} E_{PS}\\ \end{array}\right]&= \left[ \begin{array}{ccc} c_1 &{} ... &{} c_1 \\ . &{} . &{} . \\ c_P &{} . &{} c_P \\ \end{array}\right] + \left[ \begin{array}{ccc} d_1 &{} ... &{} d_S \\ . &{} . &{} . \\ d_1 &{} ... &{} d_S \end{array}\right] \nonumber \\&\quad + \left[ \begin{array}{ccc} N_{1 1} &{} ... &{} N_{1 M} \\ . &{} . &{} .\\ N_{P 1} &{} ... &{} N_{P M} \\ \end{array}\right] \left[ \begin{array}{ccc} A_{1s} &{} ... &{} A_{1S}\\ . &{} . &{} .\\ A_{Ms} &{} ... &{} A_{MS} \end{array}\right] \nonumber \\&\quad + \left[ \begin{array}{ccc} \epsilon &{} ... &{} \epsilon \\ . &{} . &{} .\\ \epsilon &{} ... &{} \epsilon \end{array}\right] \end{aligned}$$

Column-centering *E* sets $d_s$ to zero for each sample. Similarly, row-centering *E* sets $c_p$ to zero for each gene. After row and column centering, the model becomes:

5 $$\begin{aligned} \left[ \begin{array}{ccc} E_{1 s}' &{} ... &{} E_{1 S}'\\ . &{} . &{} .\\ E_{P s}' &{} ... &{} E_{PS}'\\ \end{array}\right] = \left[ \begin{array}{ccc} N_{1 1} &{} ... &{} N_{1 M} \\ . &{} . &{} .\\ N_{P 1} &{} ... &{} N_{P M} \\ \end{array}\right] \left[ \begin{array}{ccc} A_{1s}' &{} ... &{} A_{1S}'\\ . &{} . &{} .\\ A_{Ms}' &{} ... &{} A_{MS}' \end{array}\right] + \left[ \begin{array}{ccc} \epsilon &{} ... &{} \epsilon \\ . &{} . &{} .\\ \epsilon &{} ... &{} \epsilon \end{array}\right] \end{aligned}$$

where $E'_{ps}$ represents the deviation in expression from the average expression for gene *p* across all samples and $A'_{ms}$ the deviation in *cis*-regulatory activity from the average *cis*-regulatory activity for gene *p* across all samples. The model is allowed to have a non-zero intercept, therefore the model we fit is in effect:

6 $$\begin{aligned} \left[ \begin{array}{ccc} E_{1 s}' &{} ... &{} E_{1 S}'\\ . &{} . &{} .\\ E_{P s}' &{} ... &{} E_{PS}'\\ \end{array}\right]&= \left[ \begin{array}{cccc} 1 &{} N_{1 1} &{} ... &{} N_{1 M} \\ . &{} . &{} . &{} .\\ 1 &{} N_{P 1} &{} ... &{} N_{P M} \\ \end{array}\right] \left[ \begin{array}{cccc} A_0s' &{} A_{1s}' &{} ... &{} A_{1S}'\\ . &{} . &{} . &{} .\\ A_0s' &{} A_{Ms}' &{} ... &{} A_{MS}' \end{array}\right] \nonumber \\&\quad + \left[ \begin{array}{ccc} \epsilon &{} ... &{} \epsilon \\ . &{} . &{} .\\ \epsilon &{} ... &{} \epsilon \end{array}\right] \end{aligned}$$

MARA [29] uses using ridge regression and selects the regularization parameter $\lambda$ using 5-fold cross-validation. $\lambda$ controls for overfitting by imposing a so-called “budget” on TE activities. This method addresses the curse of dimensionality (too many predictors with respect to the number of observations) and stability issues arising when there is a high collinearity in the space of predictors. However, the statistical significance of each predictor is more difficult to compute than in the standard linear regression setting. Additionally, in the MARA model, each activity deviates from a mean activity corresponding to a baseline regulatory state which can be hard to describe in biological terms. Instead, we chose to consider samples in pairs. We contrasted samples from condition 2 (e.g., treatment samples) with samples from condition 1 (e.g., control samples). Under the normalized MARA-like model:

7 $$\begin{aligned} E_{ps}' = A_{0s} + \sum _{m} N_{pm} A_{ms}' + \epsilon \end{aligned}$$

We are interested in contrasting two samples labeled sample 1 and sample 2.

8 $$\begin{aligned} E_{p2}' - E_{p1}' = A_{02} + \sum _{m} N_{pm} A_{m2}' + \epsilon _2 - \left( A_{01} + \sum _{m} N_{pm} A_{m1}' + \epsilon _1\right) \end{aligned}$$

Therefore, we obtain:

9 $$\begin{aligned} \Delta E_{p,2-1}' = \Delta A_{0, 2-1} + \sum _{m} N_{pm} \Delta A_{m,2-1}' + \epsilon _{2+1} \end{aligned}$$

We used (Eq. 9) as a model with identically and independently normal-distributed noise to estimate differences in activity between treatment and control samples. We then tested whether each estimated activity was t-distributed around 0. We controlled the false discovery rate using the Benjamini-Hochberg procedure. Paired replicates were treated by concatenating the vectors of differences in expression $\Delta E_{p,2-1}'$ for each pair. The susceptibility matrix *N* was expanded row-wise accordingly.

### Computing the regulatory susceptibilities of each gene to TE subfamilies

The genomic locations of TEs were derived from Repeatmasker RELEASE 20170127, based on the hg19/GRCh37 assembly of the human reference genome. RepeatMasker annotates TEs based on sequence similarity to a consensus sequence which tends to fragment partially degenerated integrants into multiple sequences. To avoid counting fragmented TEs several times, we merged TEs belonging to the same subfamily and the same strand separated by a genomic distance of less than 100 bp. The following steps were applied to each protein-coding gene (derived from ENSEMBL release 93 using Biomart) to designate the set of corresponding putatively *cis*-regulatory TEs. We defined gene promoter regions as clusters of transcription start sites (derived from ENSEMBL release 93 using Biomart) spaced by less than 1 kb and extended by 500 bp at their 5′ and 3′ ends. Next, we defined *cis*-regulatory windows as the union of promoter regions extended by 50 kb at their 5′ and 3′ end. We identified all TEs present within *cis*-regulatory windows. We excluded TEs overlapping promoter regions as well as TEs overlapping exons. Finally, the remaining TEs were summed per subfamily to generate a vector representing the susceptibility of the gene to putatively *cis*-regulatory TEs.

### Building the susceptibility matrix N

The TE susceptibility matrix summarizes the potential regulatory activity of TE subfamilies on protein-coding genes. *N* was built by grouping integrants by subfamilies and summing them for each gene. Therefore, $N_{i, j}$ describes the number of integrants belonging to subfamily *j* in the *cis*-regulatory window of gene *i*.

### Weighting *cis*-regulatory TEs by their distance to gene promoters

To circumvent the need for a hard distance threshold, we weighted the regulatory potential of integrants by the distance separating them from gene promoters. Let *K* be the number of integrants of TE subfamily *m* present on the same chromosome as gene *p*. The regulatory potential of subfamily *m* on gene *p* is weighted by a gaussian kernel: $N_{pm} = \sum _{K} w_{pk}, \; w_{pk} = e^{-\frac{x_{pk}^2}{2L^2}}$ where:

1. $x_{pk}$ is the distance in base pairs between the center coordinate of TE integrant *k* and the center coordinate of the closest promoter of gene *p*
2. *L* is the standard deviation (i.e., bandwidth) of the gaussian kernel, in base pairs

### Filtering *E* and *N*

Each experiment, defined as the set of treatment versus control expression vectors that will eventually form matrix *E*, was subjected to a separate filtering procedure. Genes with raw count values of less than 10 in all samples were removed from *E*. A per-column pseudo-count computed as the fifth percentile of all non-zero values in the column was added to each entry in *E*. *E* was transformed to transcript per millions (TPMs) and then log2-transformed. *E* was column-centered and then row-centered. TE subfamilies with a sum of susceptibility scores $\sum _p N_{pm}$ smaller than 150 were removed from *N*. To avoid confounding bona fide *cis*-regulatory changes with differences in expression directly attributable to experimental perturbations, e.g., KO or overexpression, we filtered out experimentally perturbed genes from *E* when applicable.

### Estimating the optimal TE-promoter regulatory distance

To estimate the optimal distance until which TE subfamilies regulate gene expression in *cis*, we built several weighted susceptibility matrices *N* by varying the values of *L* between $10^3$ and $10^{10}$ base pairs and estimated the mean validation error using a 5-fold cross-validation on the gene space. The optimal value of *L* was chosen as the one that minimized the mean validation error. To ensure that the validation errors were comparable, we kept the sets of TE subfamilies and protein-coding genes fixed across all weighted matrices *N*. To this end, we filtered *E* and *N* according to the unweighted matrix *N* built with 100-kB-wide *cis*-regulatory windows centered on gene promoters, as described above and in Fig. 1. We then filtered each weighted susceptibility matrix *N* according to the rows (protein-coding genes) and columns (TE subfamilies) contained in the unweighted susceptibility matrix *N*.

### Splitting TE subfamilies between functional and non-functional fractions

Let *F* bet the set of genomic ranges considered as functional. Each TE integrant from subfamily *m* overlapping with at least one element in *F* was assigned to the so-called “functional” fraction of subfamily *m*: $m_{functional}$. The matrix $N_{functional}$ was built as described above for *N*, considering $m_{functional}$ as a distinct subfamily. As splitting subfamilies into fractions may yield predictors, i.e., columns of $N_{functional}$, with too few putatively regulated genes to reliably estimate TE subfamily *cis*-regulatory activities, we applied the following procedure:

- TE subfamilies (including their functional fractions) that were excluded by the filtering procedure applied on *N* described above were also excluded from $N_{functional}$.
- If either the functional or the non-functional fraction of a TE subfamily showed $\sum _p N_{pm} < 100$, both fractions were removed and replaced with the corresponding column in *N*, i.e., the vector of regulatory susceptibility scores $N_{pm}$ for the entire subfamily.
- We allowed some user-specified subfamilies to be “protected” from this filtering step. These subfamilies remained split between a functional and a non-functional fraction in $N_{functional}$ irrespective of the sum of their regulatory susceptibility scores.

### Per integrant mappability scores

Coordinates of TE integrants from our curated hg19 TE database were converted to hg38 using the UCSC utilitary tool liftOver [117] and thereafter shifted in the 5’ direction by half of the genomic distance covered by a single read (single end mappability) or between the 5′ end of the forward read and the 5′ end of the reverse read (paired end). Average mappability scores over each integrant were computed using the UCSC utilitary tool bigWigAverageOverBed [118]. Mappability scores for hg38 [119] were queried as .BigWig files from the UCSC website at URL https://genome-euro.ucsc.edu, using the genome browser custom track [120] information at URL https://raw.githubusercontent.com/HanLabUNLV/TEmappability/master/hub.txt. We defined “low mappability,” resp. “high mappability” integrants as those scoring below, resp. above the median mappability in their subfamily.

### Multiple sequence alignment plots

Multiple sequence alignment (MSA) plots were made as previously described [121]. In short: DNA sequences for integrants belonging to the indicated subfamilies were aligned using MAFFT [122] with parameters –reorder -auto, and then merged using the -merge option. Positions in the alignment (columns) with more than 85% gaps were grayed out. ChIP-seq signals are scaled for each integrant (row) to the [0,1] interval before being superimposed on the alignments. Averaged (scaled) ChIP-seq signals across all integrants are plotted on top of the alignments.

### Motif search

FASTA sequences for integrants belonging to the indicated subfamilies were scanned using FIMO [97] with default parameters. We used a zero-order background model computed over all TEs.

### Statistical methods

The statistical significance of TE subfamily activities is evaluated through null hypothesis significance testing via a standard t-test, where the null hypothesis is *H*0:  the value of the associated linear regression coefficient (often referred to as $\beta$) is zero. All *p*-values reported in the manuscript are adjusted for multiple testing using the Benjamini Hochberg procedure, except when specified in the methods or main text. We reject the *H*0 when the adj. *p*-value $\le 0.05$.

### Supplementary information

**Additional file 1:** **Table S1.** Differences in TE subfamily *cis*-regulatory activities estimated by *craTEs* across experiments presented in Fig. 1C.

**Additional file 2:** **Table S2.** Enriched TE subfamilies found in the proximity of differentially expressed (DE) genes for LTR5-Hs/SVA CRISPRi g#1 using lenient DE calling (Fisher’s exact test, p-val [MYLT]0.05). The sign of the log fold change (up/downregulation) is reported in the “direction” column. DE genes were called without adjusting for multiple testing. *p*-values for the enrichment test were computed according to the hypergeometric distribution, and adjusted with the Benjamini-Hochberg procedure.

**Additional file 3:** **Table S3.** Enriched TE subfamilies found in the proximity of differentially expressed (DE) genes for LTR5-Hs/SVA CRISPRi g#1 using stringent DE calling (Fisher’s exact test, BH-adjusted p-val [MYLT]0.05).

**Additional file 4:** **Table S4.** TE subfamilies enriched for increased and/or decreased ATAC-seq peaks for LTR5-Hs/SVA CRISPRi g#1.

**Additional file 5:** **Table S5.** TE subfamilies enriched for increased and/or decreased ATAC-seq peaks for LTR5-Hs/SVA CRISPRi g#2.

**Additional file 6:** **Table S6.** TE subfamilies enriched for increased and/or decreased H3K9me3 ChIP-seq peaks for LTR5-Hs/SVA CRISPRi g#2.

**Additional file 7:** **Table S7.** Estimated *cis*-regulatory activities for the TE subfamilies and transgene overexpression experiments underlying Figs. 3A and S4.

**Additional file 8.** Related to Fig. 3. Contains estimated TE subfamily activities for the RNA-seq dataset of transgene overexpression in hESCs [47] in .csv format, available at URL: https://doi.org/10.5281/zenodo.8116824. Title of data: Transposable element (TE) subfamily *cis*-regulatory activities estimated from 441 transgene overexpression experiments in human embryonic stem cells (hESCs). Description of data: Activities were estimated using *craTEs* with the weighted susceptibility matrix N computed with $L = 2.5e5kB$. Rows are transposable element (TE) subfamilies, columns are as follows: $\cdot$ Estimate: estimated *cis*-regulatory activity, correponds to a linear regression coefficient. $\cdot$ Std. Error: standard error of linear regression coefficient. $\cdot$ *t* value: *t* value corresponding to t-test with H0: Estimate = 0 and HA: Estimate $\ne$ 0. $\cdot$ $Pr(>|t|)$ : probability (*p*-value) of observing a more extreme *t* value. $\cdot$ p_adj: *p*-values adjusted with the Benjamini Hochberg procedure. $\cdot$ TE: TE subfamily. $\cdot$ condition: concatenation of the name of the overexpressed transgene and the timepoint. $\cdot$ transgene: symbol for the overexpressed transgene. $\cdot$ timepoint: time under transgene induction via DOX treatment.

**Additional file 9.** Related to Fig. 3. Contains the full heatmap of TE subfamily *cis*-regulatory activity statistical strengths (-log10 adj. *p*-value) estimated from dox-induced TF overexpression experiments in primed hESCs [47] as described in the legend of Fig. 3A, with row and column dendrograms.

**Additional file 10.** Related to Fig. 4 and Fig. S6. Contains the consensus sequences found under regions with high EOMES or GATA6 ChIP-seq coverage [70] at functional TE subfamilies in differentiating endodermal cells.

**Additional file 11.** Figures S1–S7 [127].

**Additional file 12.** Peer review history.

## Data Availability

*craTEs* [123] is available as an open source R package at URL https://github.com/pulvercyril/crates and is distributed under the MIT License. The repository was archived on ZENODO upon submission at URL https://doi.org/10.5281/zenodo.8407480. The TE annotation database RepeatMasker library RELEASE 20170127 can be found on the RepeatMasker website accessible at URL http://repeatmasker.org/libraries/RepeatMaskerMetaData-20170127.tar.gz. The following RNA-seq datasets: naïve hESCs + CRISPRi against SVA/LTR5-Hs, primed hESCs + GFP or KLF4, naïve hESCs + GFP or ZNF611; ATAC-seq dataset: naïve hESCs + CRISPRi against SVA/LTR5-Hs g#2; ChIP-seq datasets: H3K9me3 in naïve hESCs + CRISPRi against SVA/LTR5-Hs g#2, H3K9me3/H3K27ac in primed hESCs + GFP or KLF4, H3K9me3/H3K27ac in naïve hESCs + GFP or ZNF611 can be found on the Gene Expression Omnibus (GEO) under accession number GSE117395 at URL: https://www.ncbi.nlm.nih.gov/geo/query/acc.cgi?acc=GSE117395 [5, 33]. The RNA-seq datasets of NCCIT + CRISPRa/i against LTR5-Hs, LTR5A and LTR5B can be found on GEO under accession number GSE111337 at URL https://www.ncbi.nlm.nih.gov/geo/query/acc.cgi?acc=GSE111337 [35, 39]. The RNA-seq dataset of K562 + CRISPRi against LTR2B can be found on GEO under accession number GSE136763 at URL https://www.ncbi.nlm.nih.gov/geo/query/acc.cgi?acc=GSE136763 [34, 43]. The RNA-seq dataset of transgene overexpression in hESCs can be found on the DNA Data Bank of Japan (DDBJ) Sequence Read Archive (DRA) under SRA submission number DRA006296 at URL https://ddbj.nig.ac.jp/resource/sra-submission/DRA006296 [47, 50]. The following RNA-seq and ChIP-seq datasets: RNA-seq during hESC-derived endoderm differentiation, ChIP-seq against EOMES, GATA6 and H3K27ac in hESC-derived mesendoderm can be found on GEO under accession number GSE213394 at URL https://www.ncbi.nlm.nih.gov/geo/query/acc.cgi?acc=GSE213394 [70, 71]. The following RNA-seq datasets: RNA-seq during iPSC-derived endoderm differentiation, with/without GATA6 KO or rescue can be found on GEO at accession number GSE156021 at URL https://www.ncbi.nlm.nih.gov/geo/query/acc.cgi?acc=GSE156021 [69, 124]. The following RNA-seq dataset: RNA-seq in GATA2 KO HPCs can be found on GEO at accession number GSE69797 at URL https://www.ncbi.nlm.nih.gov/geo/query/acc.cgi?acc=GSE69797 [72, 125]. The following RNA-seq, CUT &TAG and ATAC-seq datasets: RNA-seq on hESC-derived hPGCLCs, hESC-derived somatic cells, SOX15 KO hPGCLCs, CUT &TAG against SOX15 in hPGCLCs, ATAC-seq in hPGCLCs can be found on GEO at accession number GSE143345 at URL https://www.ncbi.nlm.nih.gov/geo/query/acc.cgi?acc=GSE143345 [86, 126]. The following ChIP-seq and ATAC-seq datasets: ChIP-seq against KLF4 in primed hESCs, ChIP-seq against ZNF611 in naïve hESCs, ATAC-seq in naïve hESCs + CRISPRi against SVA/LTR5-Hs g#1 can be found on the Gene Expression Omnibus (GEO) under accession number GSE208403 at URL https://www.ncbi.nlm.nih.gov/geo/query/acc.cgi? &acc=GSE208403. The regulatory susceptibility matrix N, TEs vs. promoters, 100kB-wide windows can be found on ZENODO at URL https://doi.org/10.5281/zenodo.6707955 The regulatory susceptibility matrix N, TEs vs. promoters, weighted with $L = 2.5e5$kb can be found on ZENODO at URL https://doi.org/10.5281/zenodo.8117257 The regulatory susceptibility matrices N with functional fractions, TEs vs. promoters, weighted with $L = 2.5e5$kb can be found on ZENODO at URL https://doi.org/10.5281/zenodo.8117285 The regulatory susceptibility matrices N, TEs vs. promoters, weighted with *L* in [1*e*3*kB*, 1*e*10*kB*] can be found on ZENODO at URL https://doi.org/10.5281/zenodo.8117286 The code used to process the data and generate the figures can be found at URL https://renkulab.io/gitlab/crates and can be executed directly from the renkulab platform for reproducible data science, or alternatively locally after downloading docker images: $\bullet$ https://renkulab.io/projects/crates/klf4-znf611-sva-crispri $\bullet$ https://renkulab.io/projects/crates/promoter-te-subfamilies-matrix $\bullet$ https://renkulab.io/projects/crates/hescs-activities
