# Supplementary material for: Statistical learning quantifies transposable element-mediated cis-regulation
Source: Genome Biol. 2023 Nov 10;24:258. doi: 10.1186/s13059-023-03085-7 (PMC10637000; doi:10.1186/s13059-023-03085-7)
Supplement: Supplementary file 11 — Additional file 11. Figures S1–S7 [127]. [file 13059_2023_3085_MOESM11_ESM.pdf]

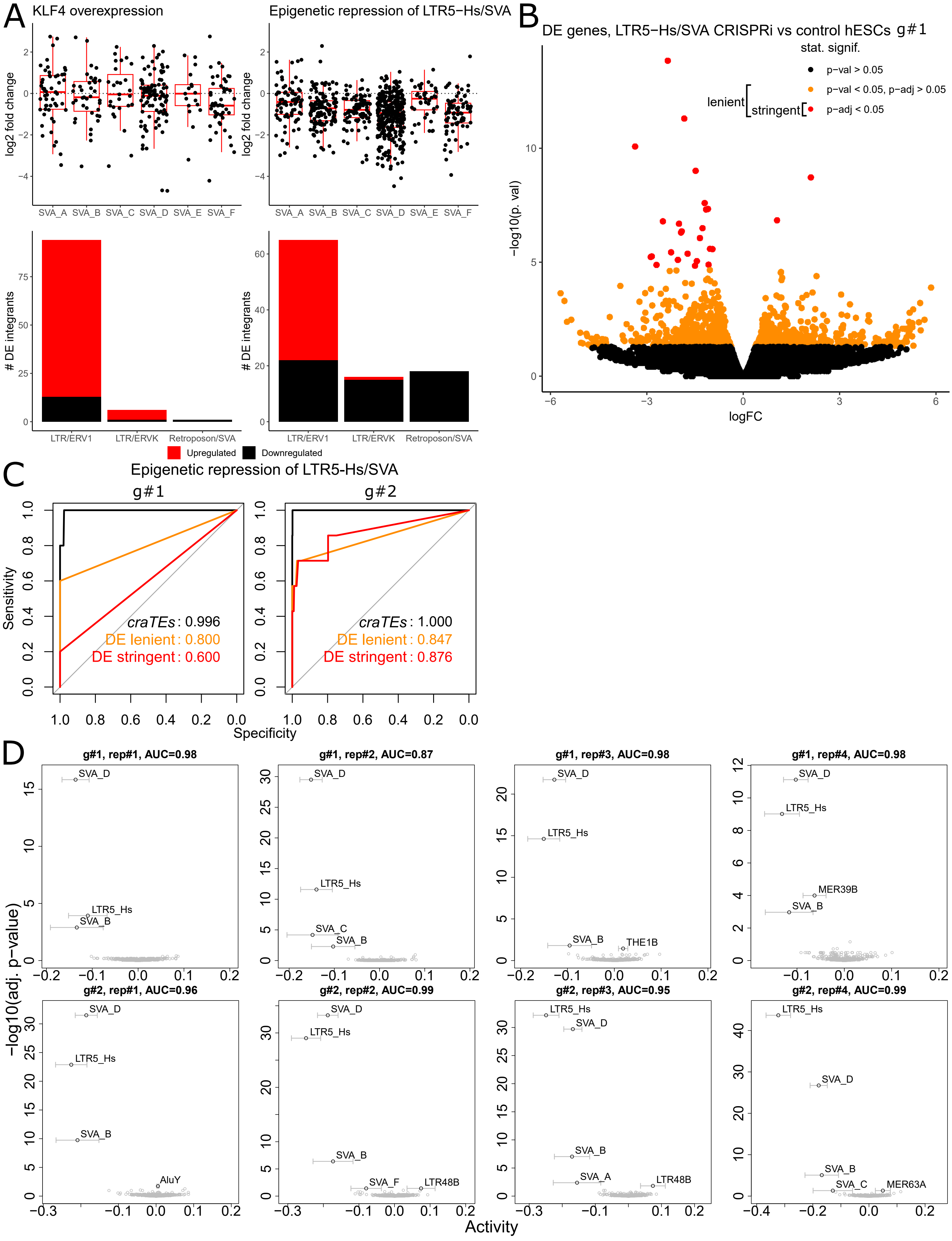
A
KLF4 overexpression
Epigenetic repression of LTR5−Hs/SVA
log2 fold change
SVA_A SVA_B SVA_C SVA_D SVA_E SVA_F
DE integrants
LTR/ERV1 LTR/ERVK Retroposon/SVA
Upregulated Downregulated
B
DE genes, LTR5−Hs/SVA CRISPRi vs control hESCs g#1
stat. signif.
p−val > 0.05
p−val < 0.05, p−adj > 0.05
p−adj < 0.05
lenient
stringent
−log10(p. val)
logFC
C
Epigenetic repression of LTR5-Hs/SVA
g#1
g#2
Sensitivity
Specificity
craTEs : 0.996
DE lenient : 0.800
DE stringent : 0.600
craTEs : 1.000
DE lenient : 0.847
DE stringent : 0.876
D
g#1, rep#1, AUC=0.98
g#1, rep#2, AUC=0.87
g#1, rep#3, AUC=0.98
g#1, rep#4, AUC=0.98
g#2, rep#1, AUC=0.96
g#2, rep#2, AUC=0.99
g#2, rep#3, AUC=0.95
g#2, rep#4, AUC=0.99
SVA_D
LTR5_Hs
SVA_B
SVA_C
THE1B
MER39B
AluY
SVA_F
LTR48B
SVA_A
MER63A
−log10(adj. p−value)
Activity

Figure S1: *craTEs* outperforms enrichment approaches based on differential expression analyses. **A** Top: Log2 fold-change (RNA-seq) for SVA integrants (top) and number of differentially expressed (DE) integrants for ERV1, HERV-K and SVA TE classes (bottom) upon KLF4 overexpression and CRISPRi-mediated repression of LTR5-Hs/SVAs [1]. **B** Volcano plot of DE analysis on protein-coding genes from the CRISPRi-mediated repression of LTR5-Hs/SVA using g#1. "Lenient" DE calling: all genes with unadjusted p-val $<$0.05, Fisher's exact test. "Stringent" DE calling: all genes with Benjamini-Hochberg adjusted [2] p-val $<$0.05. **C** ROC curves and AUCs for the classification of TE subfamilies as *cis*-regulatory vs. not *cis*-regulatory based on statistical significance using either *craTEs* or DE enrichment approaches. Ground truth: TE subfamilies that are (1) targeted by the gRNA and (2) display enrichment for differential ATAC-seq/ChIP-seq signal indicative of heterochromatin gain under CRISPRi-mediated repression of LTR5-Hs/SVAs [1]. **D** Case study for the estimation of the TE subfamily *cis*-regulatory activities in a 1 vs. 1 sample setting ($n = 1$), using each of the paired replicates in the CRISPRi-mediated repression of LTR5-Hs and SVAs in naïve hESCs [1]. AUCs as in **C**.

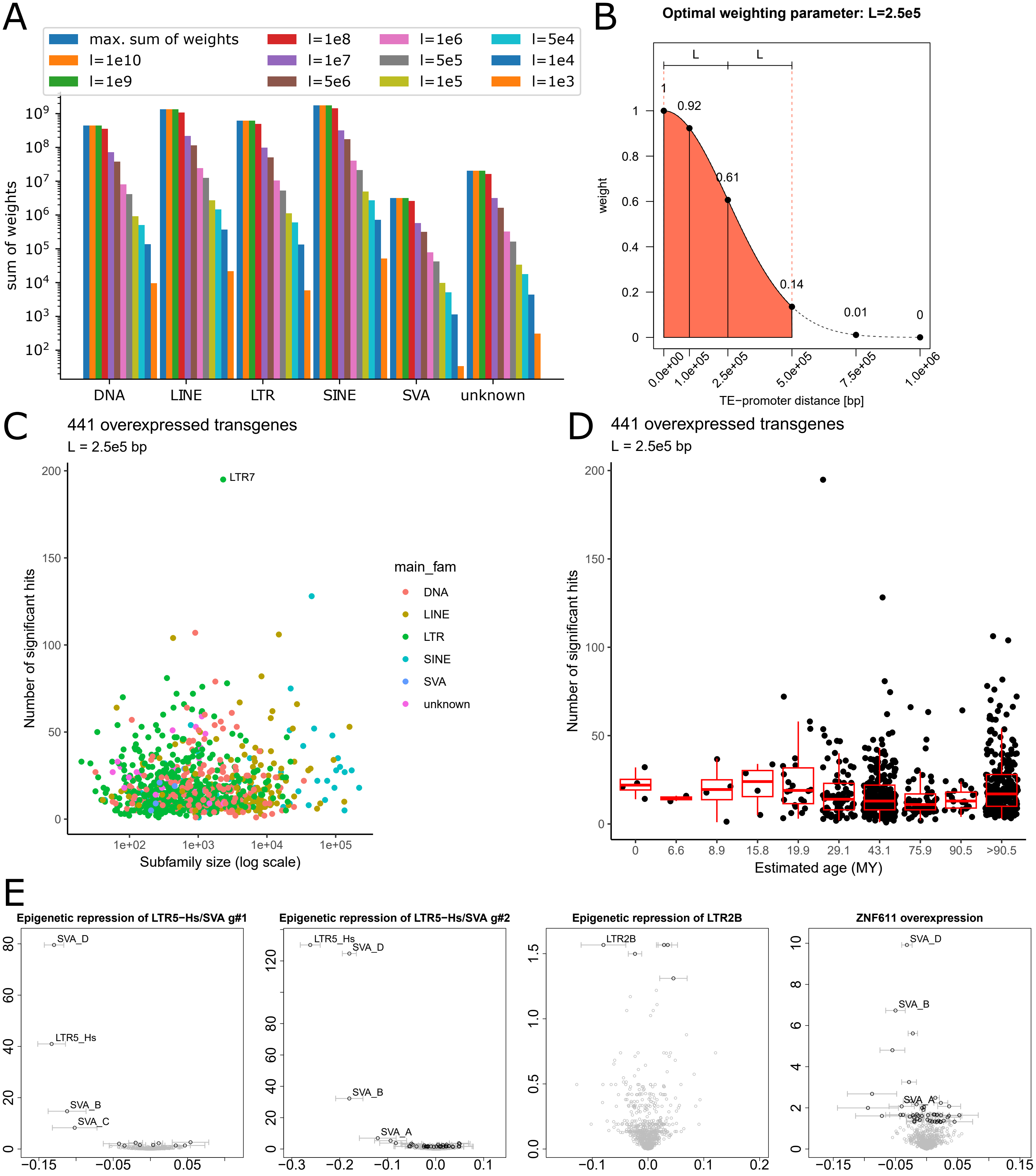
A
max. sum of weights
l=1e10
l=1e9
l=1e8
l=1e7
l=5e6
l=1e6
l=5e5
l=1e5
l=5e4
l=1e4
l=1e3
sum of weights
DNA
LINE
LTR
SINE
SVA
unknown
B
Optimal weighting parameter: L=2.5e5
weight
TE−promoter distance [bp]
C
441 overexpressed transgenes
L = 2.5e5 bp
Number of significant hits
Subfamily size (log scale)
LTR7
main_fam
D
441 overexpressed transgenes
L = 2.5e5 bp
Number of significant hits
Estimated age (MY)
E
Epigenetic repression of LTR5−Hs/SVA g#1
Epigenetic repression of LTR5−Hs/SVA g#2
Epigenetic repression of LTR2B
ZNF611 overexpression
SVA_D
LTR5_Hs
SVA_B
SVA_C
SVA_A
LTR2B

Figure S2: Related to figures 1 and 2. **A** Sum of *cis*-regulatory weights as a function of the gaussian kernel width L across each main TE families. The maximum *cis*-regulatory weight represents the limit case whereby each TE contributes to the regulation of each gene located on the same chromosome. **B** Illustration of the gaussian kernel corresponding to the optimal choice of $L = 250$kb. *cis*-regulatory weights for TE integrants located at selected distances from TSS are shown on the y-axis. The coloured area under the gaussian curve bounded by TE-promoter distances of 0 and $2L$ - i.e. 2 gaussian standard deviations - contains approx. 95% of the total area under the gaussian curve. **C** Number of times TE subfamilies reached statistical significance in the 441 transgene overexpression experiments [3] as a function of subfamily size or **D** evolutionary age. **E** Estimation of the *cis*-regulatory activity of TE subfamilies upon (left to right) CRISPRi-mediated epigenetic repression of LTR5-Hs/SVAs [1], CRISPRi-mediated epigenetic repression of LTR2B [4], overexpression of ZNF611 [1] using the matrix N computed with $L = 250$kb

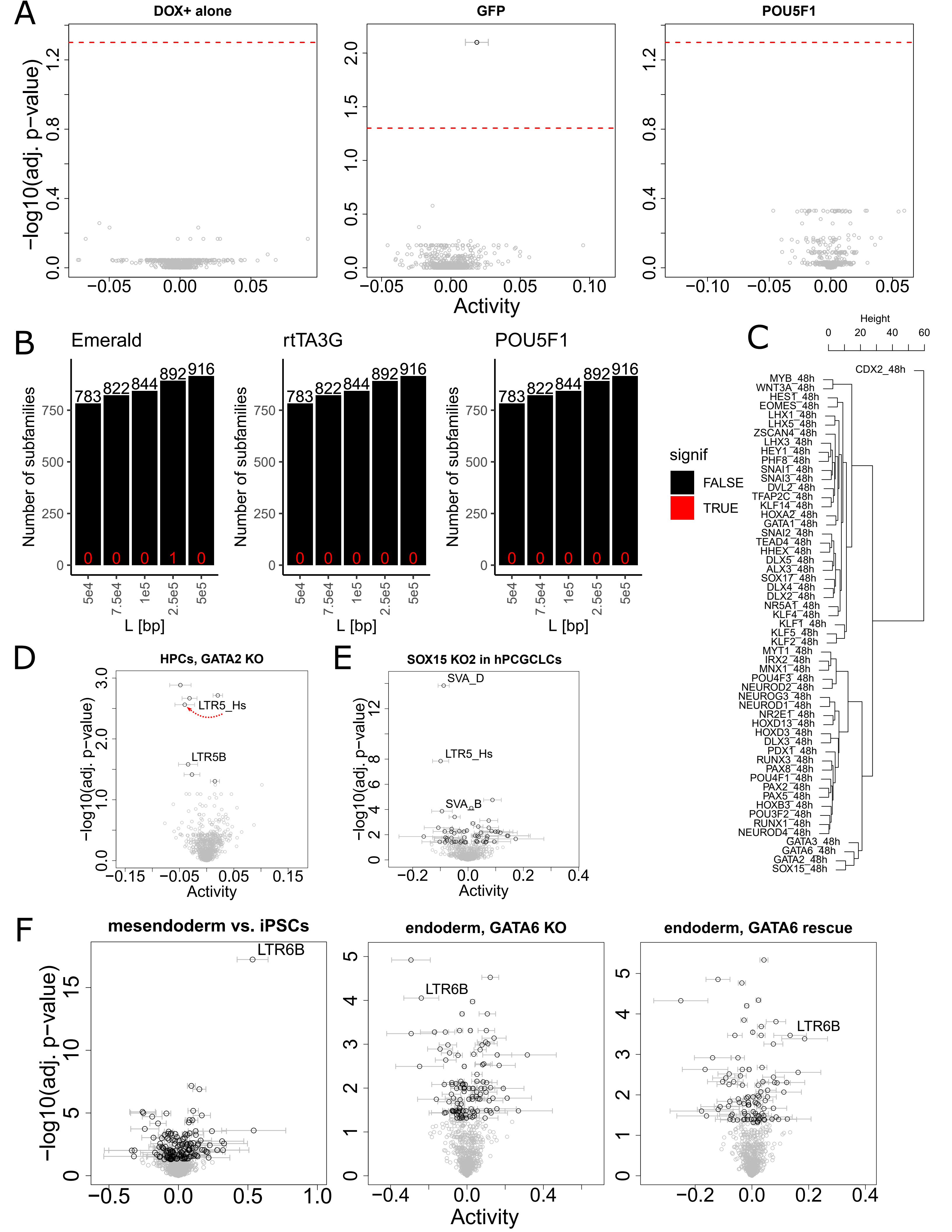

A
DOX+ alone
GFP
POU5F1
−log10(adj. p−value)
Activity
B
Emerald
rtTA3G
POU5F1
Number of subfamilies
783 822 844 892 916
0 0 0 1 0
0 0 0 0 0
0 0 0 0 0
5e4 7.5e4 1e5 2.5e5 5e5
L [bp]
signif
FALSE
TRUE
C
Height
0 20 40 60
CDX2_48h
MYB_48h
WNT3A_48h
HES1_48h
EOMES_48h
LHX1_48h
LHX5_48h
ZSCAN4_48h
LHX3_48h
HEY1_48h
PHF8_48h
SNAI1_48h
SNAI3_48h
DVL2_48h
TFAP2C_48h
KLF14_48h
HOXA2_48h
GATA1_48h
SNAI2_48h
TEAD4_48h
HHEX_48h
DLX5_48h
ALX3_48h
SOX17_48h
DLX4_48h
DLX2_48h
NR5A1_48h
KLF4_48h
KLF1_48h
KLF5_48h
KLF2_48h
MYT1_48h
IRX2_48h
MNX1_48h
POU4F3_48h
NEUROD2_48h
NEUROG3_48h
NEUROD1_48h
NR2E1_48h
HOXD13_48h
HOXD3_48h
DLX3_48h
PDX1_48h
RUNX3_48h
PAX8_48h
POU4F1_48h
PAX2_48h
PAX5_48h
HOXB3_48h
POU3F2_48h
RUNX1_48h
NEUROD4_48h
GATA3_48h
GATA6_48h
GATA2_48h
SOX15_48h
D
HPCs, GATA2 KO
LTR5_Hs
LTR5B
-log10(adj. p−value)
Activity
E
SOX15 KO2 in hPCGCLCs
SVA_D
LTR5_Hs
SVA_B
-log10(adj. p−value)
Activity
F
mesendoderm vs. iPSCs
LTR6B
endoderm, GATA6 KO
LTR6B
endoderm, GATA6 rescue
LTR6B
−log10(adj. p−value)
Activity

Figure S3: Related to figure 3. **A** Estimation of the differences in TE subfamily *cis*-regulatory activity triggered by dox-treatment alone, $n = 1$(left), dox-induced GFP overexpression, $n = 2$ (middle) and dox-induced POU5F1 overexpression, $n = 3$ (right) compared to the corresponding untreated cell lines in the "perturbome" dataset [3]. Dotted red line: statistical significance threshold. **B** Number of statistically significant differences (red), resp. non-significant differences (black) in TE-dependent *cis*-regulatory activities found for dox-induced GFP overexpression (left), dox-treatment alone (center) and dox-induced POU5F1 overexpression (right) found for distance-weighted susceptibility matrices N derived using various bandwidths L. **C** Dendrogram obtained from performing complete linkage hierarchical clustering on Euclidean distances computed from statistical significance on the transgene overexpression experiments and TE subfamilies shown in fig. 3A. **D** Estimated differences in TE-subfamily *cis*-regulatory activities upon GATA2 KO in hematopoietic progenitor cells (HPCs), $n = 2$ [5], **E** DP hPGCLCs vs DN somatic cells at day 6 of differentiation, replicate 2 $n = 2$ [6], **F** iPSC-derived mesendoderm vs. iPSCs, $n = 2$ [7] (left), GATA6 KO in iPSC-derived endoderm, $n = 2$ (center) and GATA6 rescue in GATA6 KO iPSC-derived endoderm, $n = 2$.

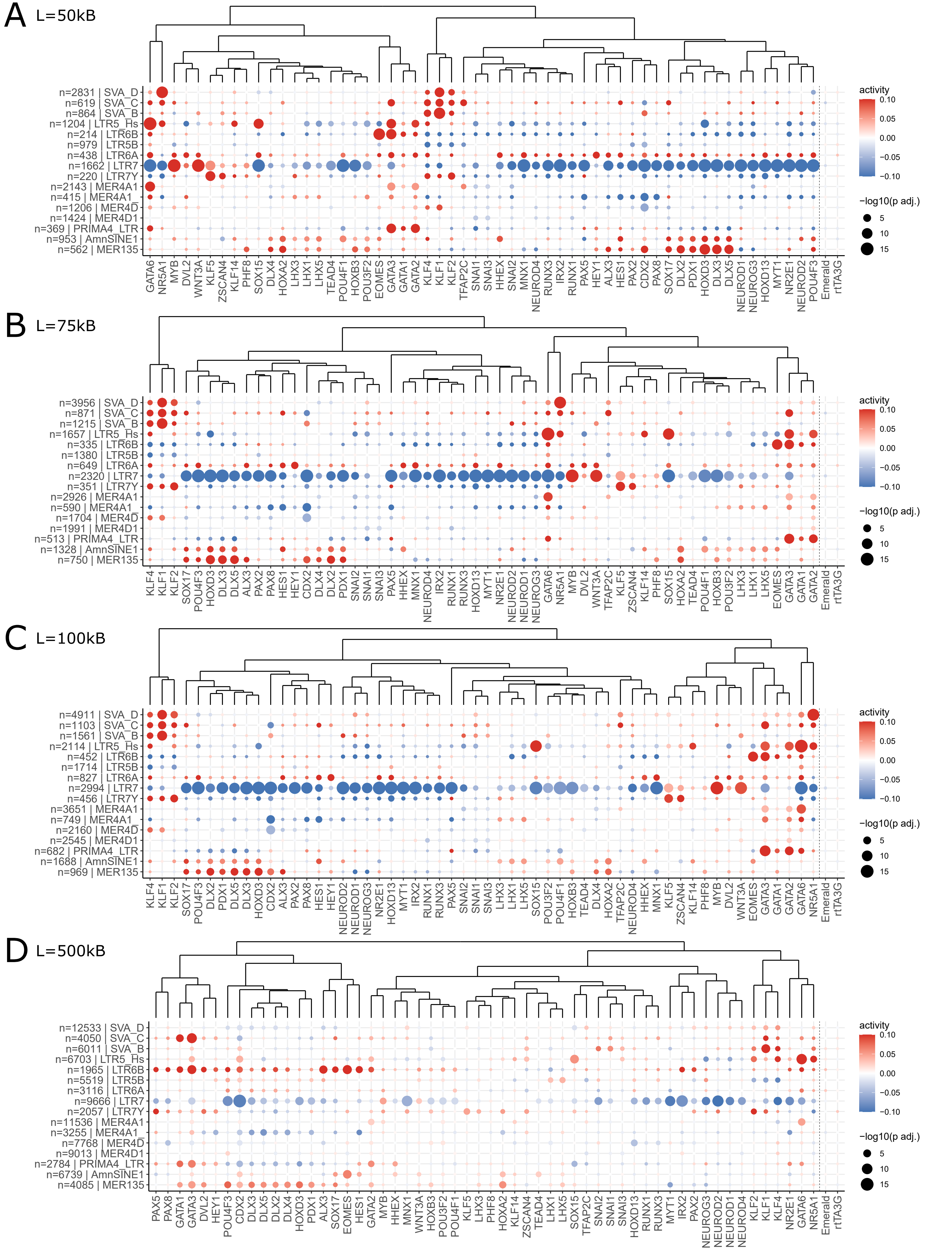
A L=50kB
n=2831 | SVA_D
n=619 | SVA_C
n=864 | SVA_B
n=1204 | LTR5_Hs
n=214 | LTR6B
n=979 | LTR5B
n=438 | LTR6A
n=1662 | LTR7
n=220 | LTR7Y
n=2143 | MER4A1
n=415 | MER4A1_
n=1206 | MER4D
n=1424 | MER4D1
n=369 | PRIMA4_LTR
n=953 | AmnSINE1
n=562 | MER135
GATA6 NR5A1 MYB DVL2 WNT3A KLF5 ZSCAN4 KLF14 PHF8 SOX15 DLX4 HOXA2 LHX3 LHX1 LHX5 TEAD4 POU4F1 HOXB3 POU3F2 EOMES GATA3 GATA1 GATA2 KLF4 KLF1 KLF2 TFAP2C SNAI1 SNAI3 HHEX SNAI2 MNX1 NEUROD4 RUNX3 IRX2 RUNX1 PAX5 HEY1 ALX3 HES1 PAX2 CDX2 PAX8 SOX17 DLX2 PDX1 HOXD3 DLX3 DLX5 NEUROD1 NEUROG3 HOXD13 MYT1 NR2E1 NEUROD2 POU4F3 Emerald rtTA3G
activity
0.10
0.05
0.00
−0.05
−0.10
−log10(p adj.)
5
10
15
B L=75kB
n=3956 | SVA_D
n=871 | SVA_C
n=1215 | SVA_B
n=1657 | LTR5_Hs
n=335 | LTR6B
n=1380 | LTR5B
n=649 | LTR6A
n=2320 | LTR7
n=351 | LTR7Y
n=2926 | MER4A1
n=590 | MER4A1_
n=1704 | MER4D
n=1991 | MER4D1
n=513 | PRIMA4_LTR
n=1328 | AmnSINE1
n=750 | MER135
KLF4 KLF1 KLF2 SOX17 POU4F3 HOXD3 DLX3 DLX5 ALX3 PAX2 PAX8 HES1 HEY1 CDX2 DLX4 DLX2 PDX1 SNAI2 SNAI1 SNAI3 PAX5 HHEX MNX1 NEUROD4 IRX2 RUNX1 RUNX3 HOXD13 MYT1 NR2E1 NEUROD2 NEUROD1 NEUROG3 GATA6 NR5A1 MYB DVL2 WNT3A TFAP2C KLF5 ZSCAN4 KLF14 PHF8 SOX15 HOXA2 TEAD4 POU4F1 HOXB3 POU3F2 LHX3 LHX1 LHX5 EOMES GATA3 GATA1 GATA2 Emerald rtTA3G
activity
0.10
0.05
0.00
−0.05
−0.10
−log10(p adj.)
5
10
15
C L=100kB
n=4911 | SVA_D
n=1103 | SVA_C
n=1561 | SVA_B
n=2114 | LTR5_Hs
n=452 | LTR6B
n=1714 | LTR5B
n=827 | LTR6A
n=2994 | LTR7
n=456 | LTR7Y
n=3651 | MER4A1
n=749 | MER4A1_
n=2160 | MER4D
n=2545 | MER4D1
n=682 | PRIMA4_LTR
n=1688 | AmnSINE1
n=969 | MER135
KLF4 KLF1 KLF2 SOX17 POU4F3 DLX2 PDX1 DLX5 DLX3 HOXD3 CDX2 ALX3 PAX2 PAX8 HES1 HEY1 NEUROD2 NEUROD1 NEUROG3 NR2E1 HOXD13 MYT1 IRX2 RUNX1 RUNX3 PAX5 SNAI2 SNAI1 SNAI3 LHX3 LHX1 LHX5 SOX15 POU3F2 POU4F1 HOXB3 TEAD4 DLX4 HOXA2 TFAP2C NEUROD4 HHEX MNX1 KLF5 ZSCAN4 KLF14 PHF8 MYB DVL2 WNT3A EOMES GATA3 GATA1 GATA2 GATA6 NR5A1 Emerald rtTA3G
activity
0.10
0.05
0.00
−0.05
−0.10
−log10(p adj.)
5
10
15
D L=500kB
n=12533 | SVA_D
n=4050 | SVA_C
n=6011 | SVA_B
n=6703 | LTR5_Hs
n=1965 | LTR6B
n=5519 | LTR5B
n=3116 | LTR6A
n=9666 | LTR7
n=2057 | LTR7Y
n=11536 | MER4A1
n=3255 | MER4A1_
n=7768 | MER4D
n=9013 | MER4D1
n=2784 | PRIMA4_LTR
n=6739 | AmnSINE1
n=4085 | MER135
PAX5 PAX8 GATA1 GATA3 DVL2 HEY1 POU4F1 CDX2 DLX3 DLX5 DLX2 DLX4 HOXD3 PDX1 ALX3 SOX17 EOMES HES1 GATA2 MYB HHEX MNX1 WNT3A HOXB3 POU3F2 POU4F3 KLF5 LHX3 PHF8 HOXA2 KLF14 ZSCAN4 TEAD4 LHX1 LHX5 SOX15 TFAP2C SNAI2 SNAI1 SNAI3 HOXD13 RUNX1 RUNX3 MYT1 IRX2 PAX2 NEUROG3 NEUROD2 NEUROD1 NEUROD4 KLF2 KLF1 KLF4 NR2E1 GATA6 NR5A1 Emerald rtTA3G
activity
0.10
0.05
0.00
−0.05
−0.10
−log10(p adj.)
5
10
15

Figure S4: Estimated differences in TE subfamily *cis*-regulatory activities are robust to variations in L. **A-D** TE-dependent *cis*-regulatory activities, as in fig. 3A, derived from N matrices with different values of L.

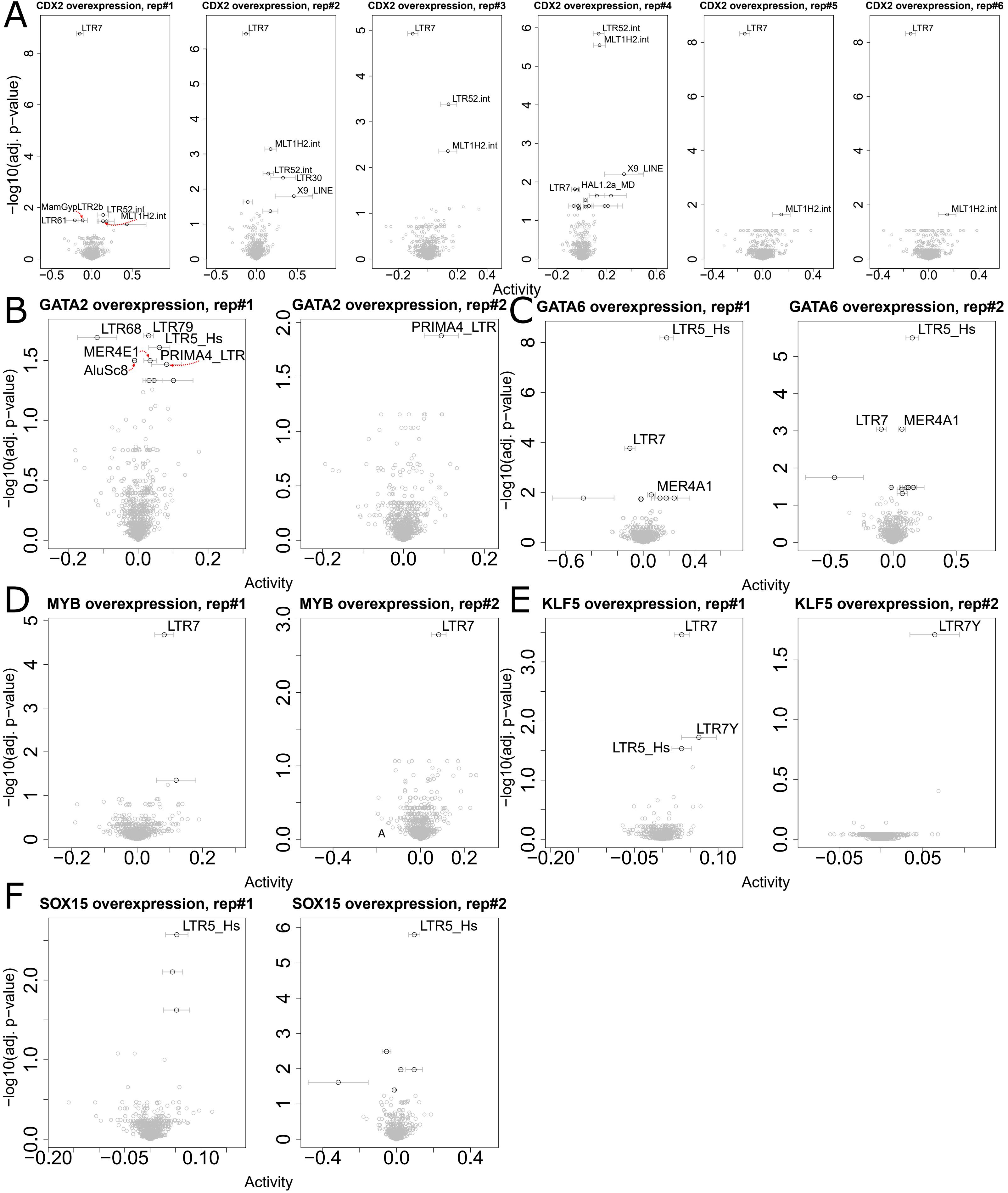
A
CDX2 overexpression, rep#1
CDX2 overexpression, rep#2
CDX2 overexpression, rep#3
CDX2 overexpression, rep#4
CDX2 overexpression, rep#5
CDX2 overexpression, rep#6
LTR7
LTR52.int
MLT1H2.int
LTR30
X9_LINE
MamGypLTR2b
LTR61
HAL1.2a_MD
−log10(adj. p−value)
Activity
B
GATA2 overexpression, rep#1
GATA2 overexpression, rep#2
LTR68
LTR79
LTR5_Hs
MER4E1
PRIMA4_LTR
AluSc8
C
GATA6 overexpression, rep#1
GATA6 overexpression, rep#2
MER4A1
D
MYB overexpression, rep#1
MYB overexpression, rep#2
E
KLF5 overexpression, rep#1
KLF5 overexpression, rep#2
LTR7Y
F
SOX15 overexpression, rep#1
SOX15 overexpression, rep#2

Figure S5: Individual replicates from the hESC "perturbome" dataset show consistent estimated differences in *cis*-regulatory activities. **A** Estimated TE-dependent *cis*-regulatory activities across individual replicates ($n = 1$) of transgene overexpression experiments [3] for CDX2, **B** GATA2, **C** GATA6, **D** MYB, **E** KLF5 and **F** SOX15.

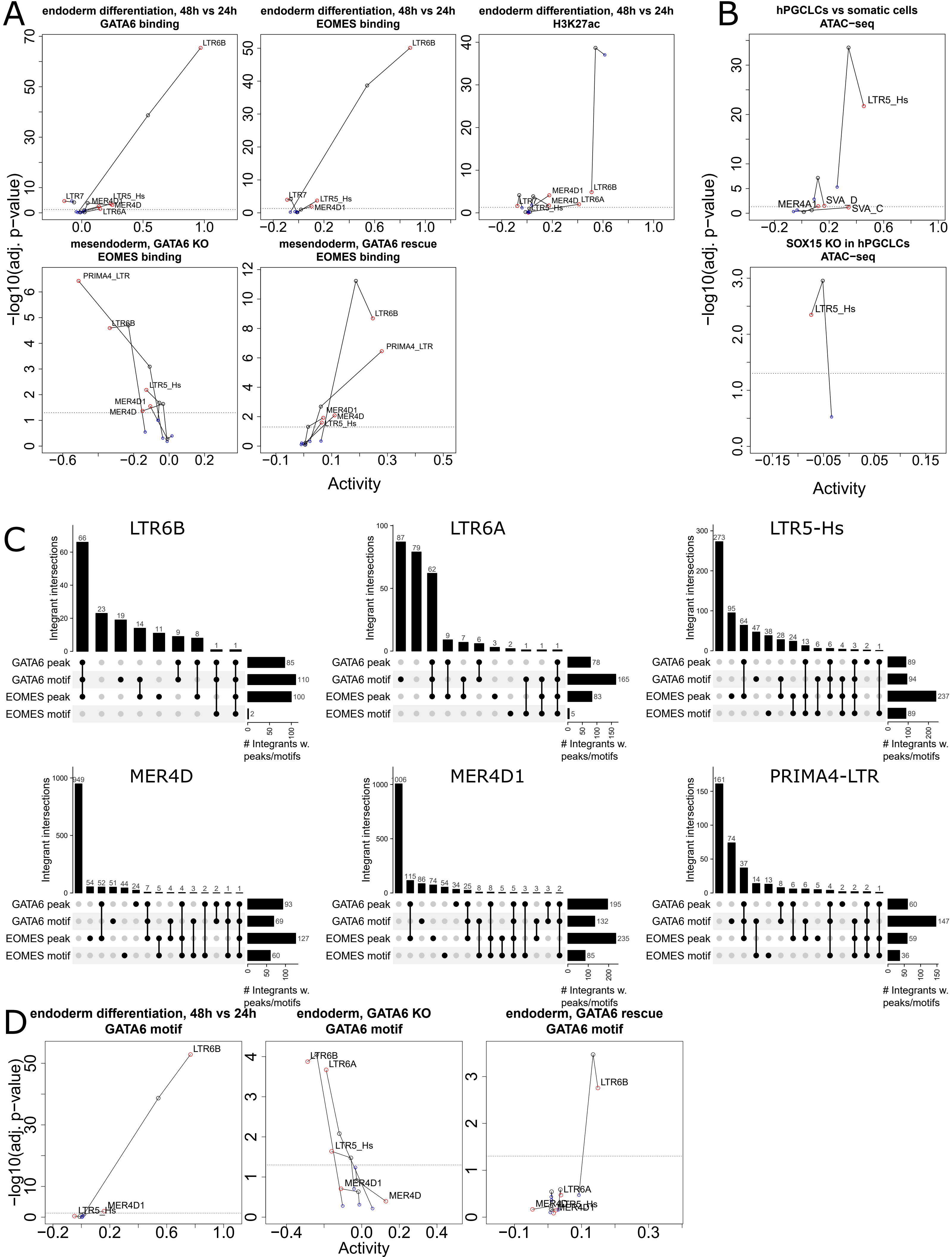
A
endoderm differentiation, 48h vs 24h
GATA6 binding
LTR6B
LTR7
LTR5_Hs
MER4D1
MER4D
LTR6A
endoderm differentiation, 48h vs 24h
EOMES binding
LTR6B
LTR7
LTR5_Hs
MER4D1
endoderm differentiation, 48h vs 24h
H3K27ac
MER4D1
LTR6B
LTR7
MER4D
LTR6A
LTR5_Hs
mesendoderm, GATA6 KO
EOMES binding
PRIMA4_LTR
LTR6B
LTR5_Hs
MER4D1
MER4D
mesendoderm, GATA6 rescue
EOMES binding
LTR6B
PRIMA4_LTR
MER4D1
MER4D
LTR5_Hs
−log10(adj. p−value)
Activity
B
hPGCLCs vs somatic cells
ATAC−seq
LTR5_Hs
MER4A1
SVA_D
SVA_C
SOX15 KO in hPGCLCs
ATAC−seq
LTR5_Hs
−log10(adj. p−value)
Activity
C
LTR6B
LTR6A
LTR5-Hs
MER4D
MER4D1
PRIMA4-LTR
Integrant intersections
GATA6 peak
GATA6 motif
EOMES peak
EOMES motif
Integrants w.
peaks/motifs
D
endoderm differentiation, 48h vs 24h
GATA6 motif
LTR6B
MER4D1
LTR5_Hs
endoderm, GATA6 KO
GATA6 motif
LTR6B
LTR6A
LTR5_Hs
MER4D1
MER4D
endoderm, GATA6 rescue
GATA6 motif
LTR6B
LTR6A
MER4D
LTR5_Hs
MER4D1
−log10(adj. p−value)
Activity

Figure S6: Related to figure 4. **A** Estimated differences in *cis*-regulatory activities for the functional (top left: GATA6-bound, center: top center: EOMES-bound, top right: marked by H3K27ac, bottom: EOMES-bound [8]) vs. non-functional fractions of selected TE subfamilies during hESC-derived endoderm differentiation, 48h vs. 24h, $n = 3$ [8] (top) and GATA6 KO (bottom left) and rescue (bottom center) in iPSC-derived mesendoderm, $n = 2$ [7]. **B** Functional (top: marked by ATAC-seq reads, bottom: SOX15-bound) vs. non-functional estimated differences in TE-dependent *cis*-reguatory activities in DP vs. DN hPGCLCs (top) and in SOX15KO vs. wild-type DP hPGCLCs (bottom) at day 6, $n = 2$ [6]. **C** Subfamily-restricted upset plots [9] showing all intersections between the following sets of integrants: those overlapping GATA6 peaks [8], GATA6 DNA-binding motifs as located by FIMO [10], EOMES peaks and EOMES-DNA binding motifs. **D** Estimated differences in *cis*-regulatory activities for functional (contains a GATA6 DNA-binding motif) vs. non-functional fractions of selected TE subfamilies during endoderm differentiation, $n = 3$ [8] (left), upon GATA6 KO in iPSC-derived endoderm, $n = 2$ [7] (center) and GATA6 rescue in iPSC-derived endoderm, $n = 2$ (right).

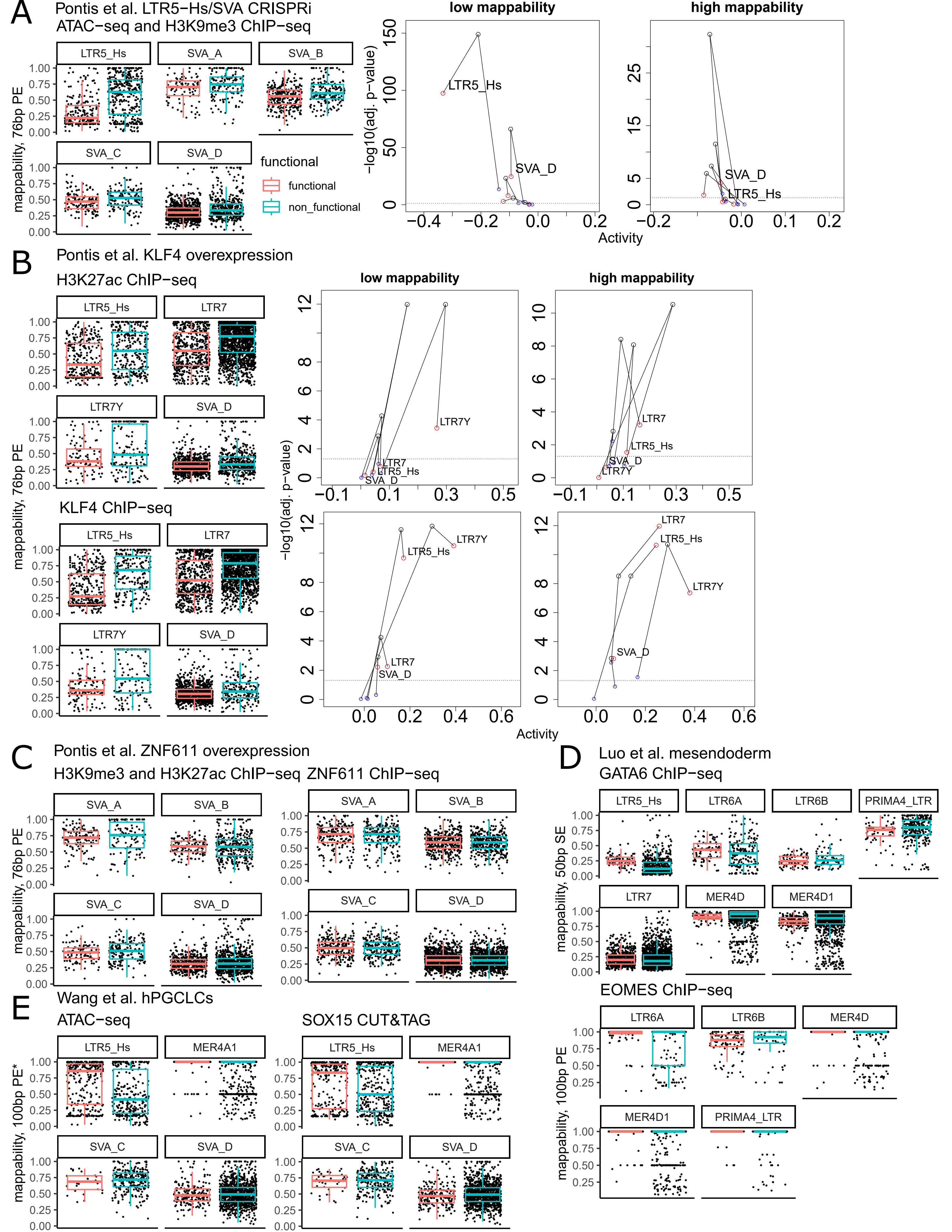
A
Pontis et al. LTR5−Hs/SVA CRISPRi
ATAC−seq and H3K9me3 ChIP−seq
LTR5_Hs
SVA_A
SVA_B
SVA_C
SVA_D
functional
functional
non_functional
mappability, 76bp PE
low mappability
high mappability
−log10(adj. p−value)
Activity
B
Pontis et al. KLF4 overexpression
H3K27ac ChIP−seq
LTR7
LTR7Y
KLF4 ChIP−seq
C
Pontis et al. ZNF611 overexpression
H3K9me3 and H3K27ac ChIP−seq
ZNF611 ChIP−seq
D
Luo et al. mesendoderm
GATA6 ChIP−seq
LTR6A
LTR6B
PRIMA4_LTR
MER4D
MER4D1
mappability, 50bp SE
EOMES ChIP−seq
mappability, 100bp PE
E
Wang et al. hPGCLCs
ATAC−seq
SOX15 CUT&TAG
MER4A1
mappability, 100bp PE*

Figure S7: Epigenomics-informed estimates of TE subfamily *cis*-regulatory activities are robust across mappability levels **A** Left: mappability scores [11] averaged over each functional (overlapping ATAC-seq loss and/or H3K9me3 gain loci, red) and non-functional (complement, blue) integrant from relevant *cis*-regulatory TE subfamilies (fig. 4A) upon CRISPRi-mediated epigenetic repression of LTR5-Hs/SVAs in naïve hESCs, g#2 [1]. Boxplots: lower quartile, median and upper quartile of mappability scores averaged over each integrant. Right: estimated differences in *cis*-regulatory activities for mappability-matched functional (red) vs. non-functional (blue) fractions of relevant TE subfamilies upon CRISPRi-mediated repression of LTR5-Hs/SVAs, g#2, $n = 3$. Prior to the functional/non-functional split, each of the indicated subfamilies was split into a low, resp. high mappability subgroup using a median split over per-integrant mappability scores within the subfamily. **B** Mappability over functional (top left: H3K27ac gain, bottom left: KLF4 ChIP-seq peak) and non-functional integrants, and differences in *cis*-regulatory activities (right) for mappability-matched functional vs. non-functional fractions of relevant TE subfamilies upon KLF4 overexpression in primed hESCs, $n = 4$ [1]. **C** Mappability over functional (left: H3k9me3 gain and/or H3K27ac loss, right: ZNF611 binding) vs. non-functional integrants upon ZNF611 overexpression in naïve hESCs [1]. **D** Mappability over functional (top: GATA6 ChIP-seq peak, bottom: EOMES ChIP-seq peak) vs. non-functional integrants in hESC-derived mesendoderm [8]. **E** Mappability over functional (left: ATAC-seq peak, right: SOX15 CUT&TAG peak) vs. non-functional integrants in hPGCLCs [6]. The asterisk highlights that we used 100bp paired end mappability tracks, as 150bp paired end mappability tracks were not available.

References

[1] Pontis J, Planet E, Offner S, Turelli P, Duc J, Coudray A, et al.. Hominid-specific transposable elements and KRAB-ZFPs facilitate human embryonic genome activation and transcription in naïve hESCs. Gene Expression Omnibus (GEO); 2019. Accession number: GSE117395. Available from: https://www.ncbi.nlm.nih.gov/geo/query/acc.cgi?acc=GSE117395.

[2] Benjamini Y, Hochberg Y. Controlling the False Discovery Rate: A Practical and Powerful Approach to Multiple Testing. Journal of the Royal Statistical Society: Series B (Methodological). 1995 Jan;57(1):289–300. Available from: https://onlinelibrary.wiley.com/doi/10.1111/j.2517-6161.1995.tb02031.x.

[3] Nakatake Y, Ko SBH, Sharov AA, Wakabayashi S, Murakami M, Sakota M, et al.. Generation and Profiling of 2,135 Human ESC Lines for the Systematic Analyses of Cell States Perturbed by Inducing Single Transcription Factors. DNA Data Bank of Japan (DDBJ); 2020. Accession number: DRA006296. Available from: https://ddbj.nig.ac.jp/resource/sra-submission/DRA006296.

[4] Deniz Ahmed M, Todd CD, Dawson MA, Branco MR. Endogenous retroviruses are a source of oncogenic enhancers in acute myeloid leukemia [RNA-Seq]. Gene Expression Omnibus (GEO); 2019. Accession number: GSE136763. Available from: https://www.ncbi.nlm.nih.gov/geo/query/acc.cgi?acc=GSE136763.

[5] Huang K, Du J, Shi X, Chen Q, Pan G. GATA2 knockout study to investigated the role of GATA2 in human hematopoiesis. Gene Expression Omnibus (GEO); 2017. Accession number: GSE69797. Available from: https://www.ncbi.nlm.nih.gov/geo/query/acc.cgi?acc=GSE69797.

[6] Wang X, Veerapandian V, Yang X, Song K, Xu X, Cui M, et al.. The chromatin accessibility landscape reveals distinct transcriptional regulation in the induction of human primordial germ cell-like cells from pluripotent stem cells. Gene Expression Omnibus (GEO); 2021. Accession number: GSE143345. Available from: https://www.ncbi.nlm.nih.gov/geo/query/acc.cgi?acc=GSE143345.

[7] Heslop JA, Pournasr B, Liu JT, Duncan SA. GATA6 defines endoderm fate by controlling chromatin accessibility during differentiation of human induced pluripotent stem cells. Gene Expression Omnibus (GEO); 2021. Accession number: GSE156021. Available from: https://www.ncbi.nlm.nih.gov/geo/query/acc.cgi?acc=GSE156021.

[8] Luo R, Huangfu D, Beer MA. Dynamic network-guided CRISPRi screen reveals CTCF loop constrained enhancer function in cell state transitions. Gene Expression Omnibus (GEO); 2022. Accession number:

GSE213394. Available from: https://www.ncbi.nlm.nih.gov/geo/query/acc.cgi?acc=GSE213394.

[9] Gu Z, Eils R, Schlesner M. Complex heatmaps reveal patterns and correlations in multidimensional genomic data. Bioinformatics. 2016 Sep;32(18):2847–2849. Available from: https://academic.oup.com/bioinformatics/article/32/18/2847/1743594.

[10] Grant CE, Bailey TL, Noble WS. FIMO: scanning for occurrences of a given motif. Bioinformatics. 2011 Apr;27(7):1017–1018. Available from: https://academic.oup.com/bioinformatics/article/27/7/1017/232614.

[11] Sexton CE, Han MV. Paired-end mappability of transposable elements in the human genome. Mobile DNA. 2019 Dec;10(1):29. Available from: https://mobilednajournal.biomedcentral.com/articles/10.1186/s13100-019-0172-5.
