# Supplementary material for: Statistical learning quantifies transposable element-mediated cis-regulation
Source: Genome Biol. 2023 Nov 10;24:258. doi: 10.1186/s13059-023-03085-7 (PMC10637000; doi:10.1186/s13059-023-03085-7)

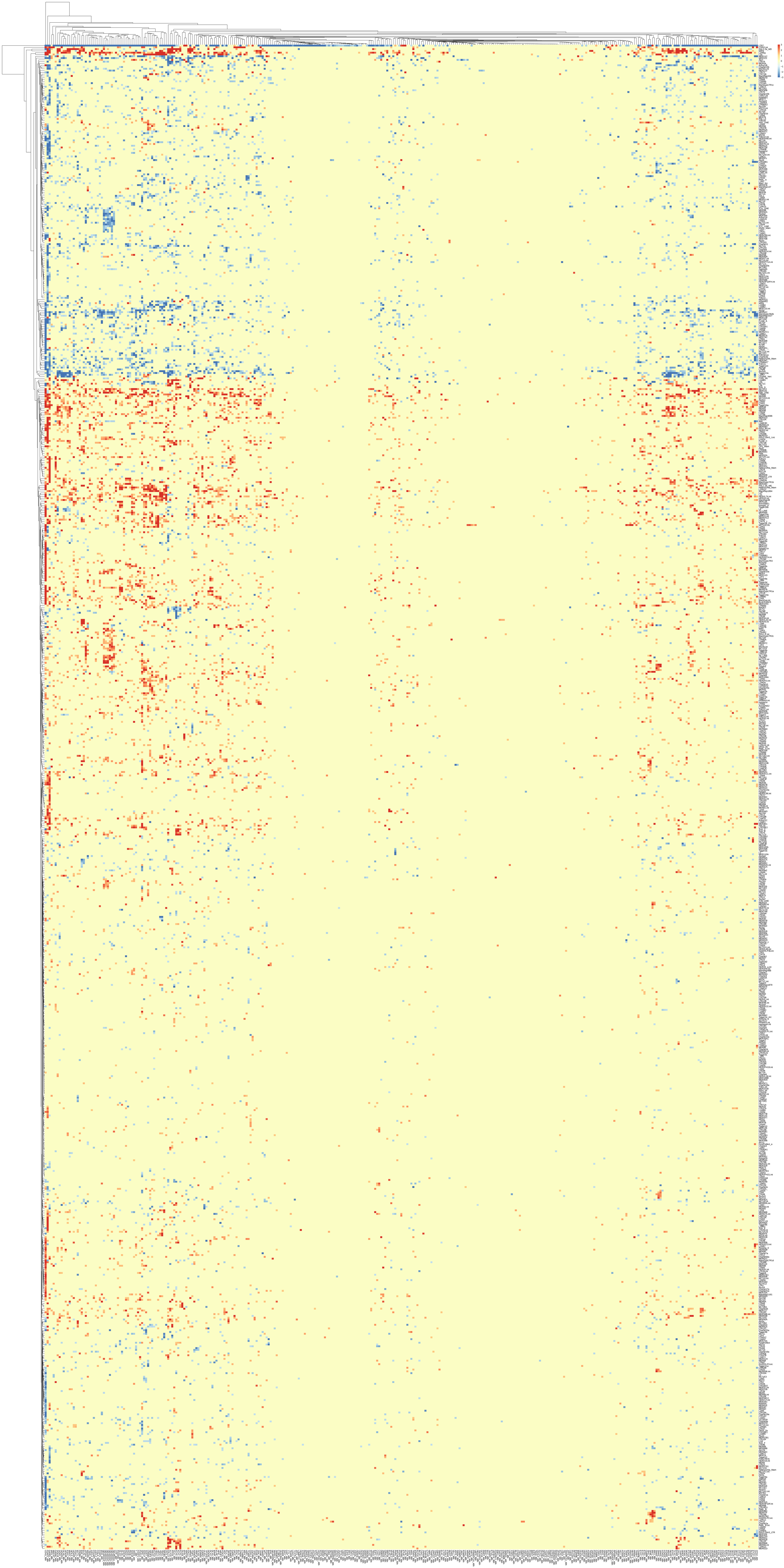

Supplement: Supplementary file 9 — Additional file 9. Related to Fig. 3. Contains the full heatmap of TE subfamily cis-regulatory activity statistical strengths (-log10 adj. p-value) estimated from dox-induced TF overexpression experiments in primed hESCs [47] as described in the legend of Fig. 3A, with row and column dendrograms. [file 13059_2023_3085_MOESM9_ESM.pdf]
