# Supplementary material for: Statistical learning quantifies transposable element-mediated cis-regulation
Source: Genome Biol. 2023 Nov 10;24:258. doi: 10.1186/s13059-023-03085-7 (PMC10637000; doi:10.1186/s13059-023-03085-7)
Supplement: Supplementary file 12 — Additional file 12. Peer review history. [file 13059_2023_3085_MOESM12_ESM.docx]

**Review history**

**First round of review**

**Reviewer 1**

Summary

The degree to which Transposable Element (TE) integrants serve as cis-regulatory elements (CREs) has become increasingly appreciated. This manuscript details a relatively simple linear model (craTEs) that models gene expression changes between two samples (e.g. a control and treatment) - in this case, mostly overexpression of TFs - as a function of the counts of each TE subfamily within cis-regulatory regions across the genome. It estimates a coefficient for each subfamily, which represents the magnitude and direction of its cis-regulatory activity. This coefficient is most easily understood, in my view, as the propensity of TFs to bind to specific classes of TEs, although this simple explanation is not explicitly stated. craTEs therefore relies only on RNA expression data and TE annotations, while previous approaches have required ChIP-seq against epigenetic marks - which are unlikely to be the root cause as they are not sequence-specific - or have used enrichment values for individual TEs among gene sets, which is a considerably less sophisticated, and tests here indicate is less sensitive.

craTEs confirms roles of previously recognized cis-regulatory TE subfamilies in gene regulation, which serves as a validation. By applying it to hundreds of transgene overexpression experiments, craTEs infers the potential involvement of several other specific TE subfamilies in early pluripotency and self-renewal gene regulatory networks. Additionally, the paper examines ChIP-seq data, and finds that TF binding sites detected by ChIP are a superior predictor of a TE integrant's cis-regulatory activity in the model, relative to ChIP-detected histone marks H3K27ac or H3K9me3, for the small number of TFs examined. This finding is of interest, although its significance is not completely clear.

Overall, craTEs is likely to be valuable for generating hypotheses regarding the cis-regulatory effects of a TE subfamily on nearby genes (such as by serving as direct binding sites for TFs), as well as the evolutionary impact of TEs on gene regulatory networks.

I have no major concerns with the manuscript, but there are some limitations that should be stated more overtly, and in a couple of examples, confounding variables should either be acknowledged or explored.

Major concerns

I do not have any major concerns about the manuscript.

General Minor Comments

1. A key limitation of craTEs is that, while it is phylogenetically informed by the fact that subfamily members share similar sequences, it cannot harness sequence similarity across subfamilies. Treating subfamilies as discrete groups may be underpowering the tool, and it may be worth mentioning in the Discussion as a possible extension to the analyses presented in the manuscript.

2. I have a few small concerns regarding the 1kb TE fragment-merging heuristic that was used to prevent counting the same TE integrant multiple times:
a. This heuristic does not account for which section of the TE each fragment contains. Therefore, it is conceivable that separate but nearby integration events are being merged.
b. Similarly, it is possible that the 1 kb distance is insufficient to unite all fragments of the same integrant. For example, a random glance at a locus in hg19 (chr1:607433-620203, specifically) reveals a single L1MA7 integrant derived from fragments that are >1kb apart. There are certainly many other integrants like this. Fragmentation is also worse for older subfamilies, and so a hard 1kb limit is likely to artificially inflate their counts and impact activity estimates. It is possible that statistical noise could be reduced with a more rigorous merging scheme, such as by using the unique ID RepeatMasker assigns to fragments likely derived from the same integration (based on strand and which parts of the repeat the fragments map to).
c. In the case of L1s, it is unclear how consensus sequences that represent segments of several subfamilies (e.g. a single 5' UTR model for related L1M subfamilies) are handled under the merging scheme, or if they are simply treated as their own subfamilies. This may artificially reduce the integrant counts of a given L1 subfamily within a gene's cis-regulatory region, and impact the power of the presented analyses.

3. In the comparison of the weighted predictor matrix to the hard-thresholded matrix, two components of the model are changed: the size of the cis-regulatory region window, and the introduction of distance-weighting across this window. It would be valuable to also compare to a hard-thresholded matrix produced using a 200 kb cis-regulatory region, so as to differentiate how much the reduction in RMSE is attributable to the use of a cis-regulatory region that includes a greater number of integrants, vs. introducing distance weighting with a guassian kernel.

4. For the analysis of "functional" vs "non-functional" TE integrants, it would seem like relatively low-hanging fruit to also differentiate the two sets based on the presence of TF binding motifs. This could supplement ChIP-seq binding analysis that was already performed for KLF4 and ZNF611, and might also hint at whether cis-regulatory TE subfamilies mediate their effects via direct binding (as mentioned in the Discussion). High-quality DNA-binding motifs for many of the TFs discussed (e.g. TFAP2C, NR5A1, KLF4, and ZNF611) already exist. Furthermore, one assumption of craTEs - that individual copies of a TE subfamily have an equivalent capacity to activate gene expression as a function of promoter-distance - is undermined by the many cases wherein the active ancestor of a TE subfamily did not contain active TF binding sites, but instead sequences that are primed to become motifs upon mutation (Fueyeo et al. 2022, Nat Rev Mol Cell Biol). It would therefore seem important for the biological interpretability of craTEs-based analyses that subfamily sequence features (i.e. binding motifs) be incorporated.

5. While RNA-seq and TE annotations alone provide sufficient statistical power to detect subfamily-specific associations with gene expression changes, the hypothesis generating value of these alone may be somewhat overstated, as integrating other forms of data (e.g. ATAC-seq, TF ChIP-seq) clearly results in a greater amount of expression variation explained by a smaller number of a subfamily's integrants. This could be a small caveat added to the Discussion, as no limitations are directly mentioned in the manuscript.

Specific Minor Comments

1. p. 11 l. 39-50: possible rendering errors or typos, where instead of 'p-val < 0.05' it says 'p-val ¡ 0.05'. This displays when the PDF is viewed with Adobe Reader or MacOS Preview.
2. p. 23 l. 41: possible typo at "resulting speciation"; perhaps this is meant to say "resulting specialization"?

**Reviewer 2**

The problem of trying to identify the cis-regulatory impact of TE families is very interesting and the approach, relying solely on RNA-seq data, is clever and promising. Overall, the authors present some interesting evidence for their model, but I have several major concerns.

1. It's unclear how their model will behave for different repeat families. In particular, does the repeat family size affect your ability to detect significant changes? Would you be able to detect whether an Alu family impact changes of expression or would the high number of instances be a problem? It's quite important to characterize what you could or couldn't detect but this is currently not described in the paper.
2. Moreover, I though the first few sections describing the result of the analysis of the CRISPRi experiments were well described and showed some clear results. However, I was less impressed by the critical analyses that led to Fig 3B and represent the main results of the paper. How significant are the effects observed? I was not able to understand the heatmap scale showed in that figure and wasn't sure if we were basically observed some marginally significant effects. For that section, it would have been useful to show the actual numbers corresponding to some of these effects. For example, if you take MER135 that you cite at the end of this section, what increase/decrease in expression was observed around how many genes? This applies to the other novel associates that you claim are important. I couldn't figure out whether these were strong or marginal effects. Additionally, showing how all these estimates were stable with changes to the parameters of your model (50Kb, 100Kb, etc.) would also have been helpful.
3. In the end, I was left with the fear that the model had been overly optimized to the training datasets used in Fig 1. Demonstrating that some of the new observations were also significant and could be validated through an orthogonal approach would have strengthen my confidence that the new predictions were real. Even the final validation section (Fig 4C), was still re-using the same TE families (LTR7, SVA, etc.), for which I'm concerned that the model may have been optimized.
4. The final claim that "TF binding may better predict TE cis-regulatory activity than differences in histone marks" is only based on two datapoints (KLF4 and ZNF611) and experiments involving the overexpression of the same factors. I worry that some of this does not really represent natural cis-regulatory activity.

Overall, I felt some of the claims were a bit overstated. See below for examples and additional comments.


P7L21. Fig 1A. I'm not sure I understand everything on this figure. What are the grey boxes? Transcripts?

P7L21. Why did you assume TEs within cis regulatory distance contributed to a significant fraction of the variation in gene expression? It is not clear what "significant" here means and within/relative to what larger context? (TF, epigenome, chromatin etc)

P8L34. I found it hard to read the equation without knowing what some of the terms where. Why not define some of the terms (e.g. Delta A_0,2-1, Delta A'_m,2-1) and then show the equation? Was unclear to me why there was a ' . Also, why is epsilon_2+1 ? Overall I found the notation to be a bit confusing.

P8L60. What model is used to test against the null hypothesis. Is that model robust to the total number of elements in the subfamily? Are you potentially only able to detect effects associated with smaller subfamilies like LTRs?

Another question is whether the age of the subfamilies is a factor. Older families might have further diverged. Would you be able to detect signal associated with that or are you restricted to younger families like SVAs?

If I understand correctly, you mainly analyzed the TE families with N>=150 and all genes >=10 reads for the model. Is it make sense to further filter out TEs with a short length? I am also wondering how many TE subfamilies were kept in the analysis. Is it also can be applied to subfamilies with a significant number of copies, like Alu subfamilies?

P9L212. The sentence of "As TEs of the same subfamily share" was repeated multiple times in the manuscript.

P9L225. You mention correctly inferring the loss of enhancer effect, but what about the value and magnitude of these losses? Are they accurate and/or interpretable at all? Numbers, if at all interpretable, would make things more convincing.

P11L31. Looks like the character < is not displaying correctly.

P11L42. I believe you refer to Fig Sup 1B here, a bit confusing. There's also no reference to Fig Sup 1A. Some comment below about Fig 1C which I think is incorrect. Even the figure caption was a bit unclear so I'm not sure I fully understand what was shown here. Perhaps you have more sensitivity, but it also looks like you have a higher rate of false positives. Perhaps your p-values are inflated?

P12L14. "are not surprisingly dropping" please rephrase

P12L37. "superior statistical power" what do you mean by superior here? Higher sensitivity?

P12L39. "reinforces the notion that TEs act as cis-regulatory fine tuners" I think this is a bit of a stretch for a result section since you didn't show anything like that.

P13L20. I think it makes sense to only look at genes on the same chromosome but if you do that, why go to 10Gb, instead of 1Gb, or even 100Mb? Seems like an overkill. I would be much more interesting to see a finer mesh of what is happening between 10Kb and 10Mb so that we could see the stability and where the actual minimum is. You point out yourself that this metric is unstable >100Mb, so why include it?

P13L35. "As 95% of the area under a gaussian curve is contained within two standard deviations from its mean, this suggests that TEs encode significant cis-regulatory information up to distances of approximately 200kb to 1 Million bases (Mb) from gene promoters" I'm not sure I fully understand this claim. At this point, it also only applies to the few families that were tested. How significant is this contribution would also be important to quantify. Also, shouldn't this extension of the model but tested on the 4 datasets from Fig 1 first before moving on to a new dataset?

P14L11. "the optimal bandwidth L took values between 50kb and 500kb", again I'm not sure this is so interesting since it's by design. A finer resolution of the optimal L within that range would be more interesting instead of discretizing in bins (50Kb, 100Kb, 500Kb, etc).

P14L58. "Both models thus use the exact same number of predictors, i.e cover the same TE subfamilies" ok but in the distance model you have extra parameters. I'm not sure the gain you make is as impressive as you claim.

P15L6. "As the number of predictors in N remained unchanged" text would need some work, there's a lot of repetition in this section.

P15L18. Fig 2E. Why only this one instead of showing all examples from Fig 1C and a more direct side by side comparison of all the families that were observed to be significant using the previous model and the ones that are now significant here. Can you explain the patters that are associated with the families that gain significance? Do they tend to have higher L values?

P15L39. "Having validated the ability of craTEs to reveal bona fide TE-dependent cis-regulatory activities" seems like a strong statement. Wouldn't be more "potentially regulatory active TEs"? It feels like the cis-regulatory activities themselves are unclear or black-boxes and more implied by the downstream result (activity shift).

P16L25. Fig 3B. I was not able to understand the color scale of this heatmap. How do these values compare to the ones you observed in the 4 experiments you used to validate the method. Are these as significant or marginal enrichment? I was also not able to find the underlying data in the supplements. Just a more comprehensive pdf.

P16L25. Fig 3B. Related to the same figure, how much do the pattern change if you change the cutoff? Is it stable even if you 50Kb? Or 200Kb? You should show that most of the associations remain even if you change the cutoff slightly.

P16L25. Fig 3B. Related to this figure again, it would be good to confirm whether replicate showed similar results. Would also be good to show more systematically that genes from the same pathways show more overlap in terms of effect versus unrelated genes.

P16L34. "suggesting that commonalities in gene expression were partially mirrored by similar cis-regulatory TE activity patterns" here it feels like there are potential confounding explanations. Could it not be a distance thing? Are the genes from same family not often closer to each other? What about it being the result of TF cascade or interactions? Perhaps it is not due to the cis-regulatory TE activity patterns but rather affecting one element in turn affects the whole family.
a. I'd test whether these TF interact together
b. The distances between these elements
c. Whether they share binding site

P17L35. "a MYB-LTR7 axis in the maintenance of self-renewal and stemness in adult cells and tissues" is this direct or indirect? Unclear to me whether most of what is observed is indirect.

P18L505. I could not see the increased AmnSINE1 activity for RUNX1 in fig.3B.

P18L483-488. A surprising contrast is mentioned but the implications are not clear. Is this a novel result? Does it change our current understanding of GATA6 expression? (discussion speculates a bit, but here its not clear what it entails.)

P19L522-524 you formed a functional group that seems to include repressive regions. Why not also the opposite, more active regions?

P20L556. "TF bindings may be better suited as a marker of cis-regulatory TE activity than changes in histone marks…". I am wonder is it specific to a certain strong TF? Or because this is based on an over-expression experiments? Have the authors test more TFs, like weak TFs or co-factors?

P21L594-596 "TE-dependent cis-regulation accounts for a fraction of the variation in gene expression comparable to that inferred from models of gene regulation based on the TFBS repertoire of core promoters." Where was that mentioned or shown?

P30L835. A typo in the sentence of "To ensure that that…"

P33L908. I am wondering is it make sense to generate the corresponding R script to perform such analysis? It would be much more valuable if the authors could convert them to easy-to-use R/python scripts.

**Reviewer 3**

Pulver et al. introduce a new method, called craTE, to infer the TE subfamilies that are controlling gene regulation. The method takes advantage of the multiple copies of TE in the genome to provide a good statistical power ensuring the analysis can be run with even a single replicate. The analysis is able to reproduce some already known results. The comparison with studies that integrated transcription of TEs shows that craTE has a higher sensitivity and is able to detect previously missed regulatory TEs. The authors test several distances and find that the TEs impact gene regulation on distances between 100-500Kb. Also, they find that TF binding at TE is more important than histone modifications. Overall, I find this paper interesting, and would benefit the field. Nevertheless, there are some outstanding points that the authors will need to address.
1. The introduction is too long. Maybe it could be shortened to focus on the main important aspects.
2. There is no implementation of the method shared. The authors should include a github/package link for the method that could be used by the community.
3. The benchmarking with other approaches is not systematic. It is not clear what metrics have been used to compare with other methods. What is a true positive, false positive, false negative and true negative?
4. One of their main finding is that TEs need to be within 500Kb to have an impact on gene activity. This is very interesting, since that would be the size of most TADs. TADs have been shown to have a regulatory role (https://genome.cshlp.org/content/32/4/682.short; https://www.biorxiv.org/content/10.1101/2022.04.29.488986v2.abstract). The authors should test whether including TAD data will improve their predictions. Instead of using a fix size, could using the specific TADs improve the results.
5. The authors found that specific histone modifications do not perform as well as TF ChIP data. This is not surprising since we know that most regulatory regions are characterized by combinations of histone modifications (https://genomebiology.biomedcentral.com/articles/10.1186/s13059-021-02532-7). The authors could add an explanation about this. Generating multiple ChIP-seq histone modifications would be beyond the scope of this manuscript, but authors could try to use chromatin state data to see if that improves predictions.

**Authors’ response to reviewers**

Reviewer reports:

Reviewer #1: Summary

The degree to which Transposable Element (TE) integrants serve as cis-regulatory elements (CREs) has become increasingly appreciated. This manuscript details a relatively simple linear model (craTEs) that models gene expression changes between two samples (e.g. a control and treatment) - in this case, mostly overexpression of TFs - as a function of the counts of each TE subfamily within cis-regulatory regions across the genome. It estimates a coefficient for each subfamily, which represents the magnitude and direction of its cis-regulatory activity. This coefficient is most easily understood, in my view, as the propensity of TFs to bind to specific classes of TEs, although this simple explanation is not explicitly stated. craTEs therefore relies only on RNA expression data and TE annotations, while previous approaches have required ChIP-seq against epigenetic marks - which are unlikely to be the root cause as they are not sequence-specific - or have used enrichment values
for individual TEs among gene sets, which is a considerably less sophisticated, and tests here indicate is less sensitive.

craTEs confirms roles of previously recognized cis-regulatory TE subfamilies in gene regulation, which serves as a validation. By applying it to hundreds of transgene overexpression experiments, craTEs infers the potential involvement of several other specific TE subfamilies in early pluripotency and self-renewal gene regulatory networks. Additionally, the paper examines ChIP-seq data, and finds that TF binding sites detected by ChIP are a superior predictor of a TE integrant's cis-regulatory activity in the model, relative to ChIP-detected histone marks H3K27ac or H3K9me3, for the small number of TFs examined. This finding is of interest, although its significance is not completely clear.

Overall, craTEs is likely to be valuable for generating hypotheses regarding the cis-regulatory effects of a TE subfamily on nearby genes (such as by serving as direct binding sites for TFs), as well as the evolutionary impact of TEs on gene regulatory networks.

I have no major concerns with the manuscript, but there are some limitations that should be stated more overtly, and in a couple of examples, confounding variables should either be acknowledged or explored.

Major concerns

I do not have any major concerns about the manuscript.

General Minor Comments

1. A key limitation of craTEs is that, while it is phylogenetically informed by the fact that subfamily members share similar sequences, it cannot harness sequence similarity across subfamilies. Treating subfamilies as discrete groups may be underpowering the tool, and it may be worth mentioning in the Discussion as a possible extension to the analyses presented in the manuscript.

We thank reviewer 1 for pointing out this limitation. One possible way to exploit across-subfamily sequence similarities would be to encourage smoothness in terms of cis-regulatory activity estimation based on the structure of the phylogenetic ancestry of TEs. Closely-related TE subfamilies would be tightly connected on graph derived from the aforementioned tree, and structural information may be exploited by penalizing large differences in activity estimation for neighboring subfamilies as is done in the fused lasso.

We have amended the discussion to emphasize this shortcoming of *craTEs*, and propose avenues to ameliorate the tool: **“*Whereas craTEs relies on sequence similarity as encoded in the TE models used by Repeatmasker, relatedness across subfamilies is currently not modeled: each subfamily is considered as phylogenetically equidistant to all others which may hamper sensitivity. One possible extension to craTEs entails encoding sequence similarity across subfamilies as a nearest-neighbour graph to constrain closely related TE subfamilies to receive similar cis-regulatory activities via penalized regression, e.g. the fused lasso.”***

2. I have a few small concerns regarding the 1kb TE fragment-merging heuristic that was used to prevent counting the same TE integrant multiple times:
a. This heuristic does not account for which section of the TE each fragment contains. Therefore, it is conceivable that separate but nearby integration events are being merged.
b. Similarly, it is possible that the 1 kb distance is insufficient to unite all fragments of the same integrant. For example, a random glance at a locus in hg19 (chr1:607433-620203, specifically) reveals a single L1MA7 integrant derived from fragments that are >1kb apart. There are certainly many other integrants like this. Fragmentation is also worse for older subfamilies, and so a hard 1kb limit is likely to artificially inflate their counts and impact activity estimates. It is possible that statistical noise could be reduced with a more rigorous merging scheme, such as by using the unique ID RepeatMasker assigns to fragments likely derived from the same integration (based on strand and which parts of the repeat the fragments map to).
c. In the case of L1s, it is unclear how consensus sequences that represent segments of several subfamilies (e.g. a single 5' UTR model for related L1M subfamilies) are handled under the merging scheme, or if they are simply treated as their own subfamilies. This may artificially reduce the integrant counts of a given L1 subfamily within a gene's cis-regulatory region, and impact the power of the presented analyses.

There was a mistake in the text and we warmly thank reviewer 1 for bringing it to our attention. Indeed, the distance used for TE merging was actually 100bp and not 1kb. We amended the Methods section accordingly and now make that explicit in the Results section. We chose this short window as previously used in Turelli et al. [https://doi.org/10.1126/sciadv.aba3200] for which we developed a closely related variation of the method. The short and therefore conservative distance of 100bp was chosen to minimize the risk of merging closely located but *bona fide* distinct integrants of the same subfamily. Whereas it is true that this merging scheme does not check the final structure of the merged elements against a reference, we verified that abnormally long integrants were not generated and manually checked that merged integrants adhered to orthodox structures by alignment to their cognate consensus.

Regarding the locus chr1:607433-620203 (https://genome-euro.ucsc.edu/cgi-bin/hgTracks?db=hg19&lastVirtModeType=default&lastVirtModeExtraState=&virtModeType=default&virtMode=0&nonVirtPosition=&position=chr1%3A607433%2D620203&hgsid=297302015_mN42JSYStaJRgnMJqz2c3fCc00MD), we are not sure we understood reviewer 1’s point. The two L1**M**A7 fragments are separated by around 8kb but there is a 6kB long L1**P**A7 in between. Taking the reference TE annotation at face value, this L1PA7 may have integrated in the L1MA7. Merging the L1MA7 would here wrongly annotate the L1PA7 as a fraction of an L1MA7. This illustrates why we chose a conservative threshold of 100bp for merging. Finally, the database used for the annotation is the one used by the program RepeatMasker and unlike models stored on DFAM, it does not consider the 5’ or 3’ ends of L1 as distinct subfamilies (http://www.repeatmasker.org/species/hg.html).

3. In the comparison of the weighted predictor matrix to the hard-thresholded matrix, two components of the model are changed: the size of the cis-regulatory region window, and the introduction of distance-weighting across this window. It would be valuable to also compare to a hard-thresholded matrix produced using a 200 kb cis-regulatory region, so as to differentiate how much the reduction in RMSE is attributable to the use of a cis-regulatory region that includes a greater number of integrants, vs. introducing distance weighting with a guassian kernel.

We thank reviewer 1 for suggesting this improvement. The optimal cis-regulatory bandwidth having increased from 100kB to 250kB due to the finer definition of the distance variation evaluation in fig. 2, the corresponding cis-regulatory window width using hard-thresholding now amounts to 500kB. Estimating TE subfamily activities according to the latter indeed explained 5.2% of the variation in gene expression, vs 4.5% and 5.4% for 100kB-wide hard thresholded windows and L=250kB gaussian-weighted cis-regulatory windows-

We amended the Results section accordingly: *“****Notably, as 500kB-wide hard-thresholded cis-regulatory windows explained 5.2% of the variance in gene expression, most of the increase in explained variance observed under the weighted (L=250kB) versus unweighted model used in fig. 1 likely stems from considering more distant TEs as putatively cis-regulatory****.”*

4. For the analysis of "functional" vs "non-functional" TE integrants, it would seem like relatively low-hanging fruit to also differentiate the two sets based on the presence of TF binding motifs. This could supplement ChIP-seq binding analysis that was already performed for KLF4 and ZNF611, and might also hint at whether cis-regulatory TE subfamilies mediate their effects via direct binding (as mentioned in the Discussion). High-quality DNA-binding motifs for many of the TFs discussed (e.g. TFAP2C, NR5A1, KLF4, and ZNF611) already exist.

We thank reviewer 1 for making this suggestion. We now include fig. S6D which shows that the presence of GATA6 motifs as assessed by FIMO aptly differentiates between functional and non-functional LTR6B integrants during endoderm differentiation as well as upon GATA6 KO and rescue. We amended the results section accordingly.

Furthermore, one assumption of craTEs - that individual copies of a TE subfamily have an equivalent capacity to activate gene expression as a function of promoter-distance - is undermined by the many cases wherein the active ancestor of a TE subfamily did not contain active TF binding sites, but instead sequences that are primed to become motifs upon mutation (Fueyeo et al. 2022, Nat Rev Mol Cell Biol).

We thank reviewer 1 for pointing this out, which likely proves most relevant for older TE subfamilies (e.g AmnSINE1, MER121, MER135) which are old enough for TF binding site maturation to occur. We amended the discussion accordingly. “**Note that craTEs cannot discriminate between waves of transposition whereby TE subfamilies spread sequences poised to acquire TFBS by gradual mutations from those whereby extant TFBS are constituents of newly transposed integrants. The former may be most relevant for older TE subfamilies e.g. AmnSINE1, MER121 and MER135 elements.”**

It would therefore seem important
for the biological interpretability of craTEs-based analyses that subfamily sequence features (i.e. binding motifs) be incorporated.

5. While RNA-seq and TE annotations alone provide sufficient statistical power to detect subfamily-specific associations with gene expression changes, the hypothesis generating value of these alone may be somewhat overstated, as integrating other forms of data (e.g. ATAC-seq, TF ChIP-seq) clearly results in a greater amount of expression variation explained by a smaller number of a subfamily's integrants. This could be a small caveat added to the Discussion, as no limitations are directly mentioned in the manuscript.

We thank reviewer 1 for highlighting that taking epigenomics data into consideration improves upon the detection of cis-regulatory activities, as made evident by Figure 4. We amended the discussion accordingly: *Using RNA-seq data derived from thoroughly characterized cases of TE-dependent cis-regulation, we showed that correctly identifies differentially active cis-regulatory TE subfamilies.* ***Moreover, we could refine activity estimations by incorporating context-matched epigenomic data – e.g TF binding or chromatin marks - into craTEs, highlighting that protein-coding gene expression and chromatin status at TEs complement each other productively for uncovering TE-dependent cis-regulation.***
Specific Minor Comments

1. p. 11 l. 39-50: possible rendering errors or typos, where instead of 'p-val < 0.05' it says 'p-val ¡ 0.05'. This displays when the PDF is viewed with Adobe Reader or MacOS Preview.

We thank reviewer 1 for noticing this formatting issue, which we now corrected.

2. p. 23 l. 41: possible typo at "resulting speciation"; perhaps this is meant to say "resulting specialization"?

We intended to convey the idea that the TE-dependent turnover of regulatory elements creates species-specific collections of CREs which – due to selective constraints – will tend to favor equivalent phenotypic outcomes, i.e. converge on phenotypes that benefit from high fitness to the environment. We amended the conclusion accordingly: *“For such vital and highly conserved events, the resulting speciation is only mechanistic owing to selective pressures.* ***In those cases, the TE-dependent and species-specific CRE turnover is likely to result in equivalent phenotypic adaptations across species, as reproductive/survival stakes leave little room for organismal novelty.****”*


Reviewer #2: The problem of trying to identify the cis-regulatory impact of TE families is very interesting and the approach, relying solely on RNA-seq data, is clever and promising. Overall, the authors present some interesting evidence for their model, but I have several major concerns.

1. It's unclear how their model will behave for different repeat families. In particular, does the repeat family size affect your ability to detect significant changes? Would you be able to detect whether an Alu family impact changes of expression or would the high number of instances be a problem? It's quite important to characterize what you could or couldn't detect but this is currently not described in the paper.

We thank reviewer 2 for requesting this important information. We now included fig. S2C and D, which show that neither subfamily size nor age appear to explain the propensity of craTEs to assign a statistically significant changes in cis-regulatory activity across 441 transgene overexpression experiments in hESCs. We amended the Results section accordingly: **“*Stratifying subfamilies according to size and evolutionary age did not reveal any discernible bias regarding the distribution of statistically significant cis-regulatory activities (fig. S2C-D).”***

2. Moreover, I though the first few sections describing the result of the analysis of the CRISPRi experiments were well described and showed some clear results. However, I was less impressed by the critical analyses that led to Fig 3B and represent the main results of the paper. How significant are the effects observed? I was not able to understand the heatmap scale showed in that figure and wasn't sure if we were basically observed some marginally significant effects. For that section, it would have been useful to show the actual numbers corresponding to some of these effects. For example, if you take MER135 that you cite at the end of this section, what increase/decrease in expression was observed around how many genes? This applies to the other novel associates that you claim are important. I couldn't figure out whether these were strong or marginal effects.

We seem to have generated a confusion by using the term “significant” in the statistical sense, i.e. to mean “statistically significant”. Importantly, statistical significance does not necessarily imply biological or functional significance. We amended the manuscript by replacing the ambiguous occurrences of “significant” with “statistically significant”.

Furthermore, we agree with reviewer 2 that in the first submission of our manuscript, Fig. 3B (now moved to fig. 3A) did not explicitly display measures of statistical significance. Instead, we had set the estimates of non-significant cis-regulatory activities (BH-adjusted p-values < 0.5) to zero. Now, we simultaneously plot statistical significance (-log10(p-adj)) and effect sizes (activity coefficients) for all subfamilies highlighted in fig. 3A. We also added the estimated number of genes participating to the estimation of the cis-regulatory TE activities on the labels of the y-axis. Finally, to facilitate interpretation of the statistical significance values and effect sizes, we included the cis-regulatory activities estimated in control samples, namely GFP overexpression and DOX-treatment alone.

Additionally, showing how all these estimates were stable with changes to the parameters of your model (50Kb,
100Kb, etc.) would also have been helpful.

We thank reviewer 2 for making this suggestion and now include a new supplementary figure S4 where the cis-regulatory activities for all TE subfamilies and transgene overexpression experiments presented in fig. 3A are plotted after running craTEs on cis-regulatory matrices N defined according to a range of L spanning a whole order of magnitude, i.e 50kB to 500kB. We amended the Results section accordingly: “***Additionally, the directionality and statistical significance of TE-dependent cis-regulatory activities were robust to varying the cis-regulatory bandwidth L (fig. S4)”***

3. In the end, I was left with the fear that the model had been overly optimized to the training datasets used in Fig 1. Demonstrating that some of the new observations were also significant and could be validated through an orthogonal approach would have strengthen my confidence that the new predictions were real. Even the final validation section (Fig 4C), was still re-using the same TE families (LTR7, SVA, etc.), for which I'm concerned that the model may have been optimized.

It is true that while we developed craTEs aiming for impartiality with respect to TE subfamilies and the scarce RNA-seq datasets that may serve as proper validations, namely subfamily-wide CRISPRa/i, we cannot definitively exclude that some level of unconscious over-optimization took place while implementing craTEs. Still, we now extensively show that the highly cis-regulatory findings of the model are robust to substantial changes in cis-regulatory distances (see our reply to the previous comment).

Nonetheless, we supplemented the manuscript, and notably fig. 4, with an in-depth analysis of the interplay of GATA6 and EOMES at LTR6B, a subfamily that does not appear in the “proof of concept” analyses displayed in fig. 1. In short, we now show that in addition to activating LTR6B as well as other primate-specific TE subfamilies (SVAs, LTR5-Hs, but also LTR6A, LTR5B, MER4A1, MER4D, MER4D1 and PRIMA4-LTR, which did not appear among the top significant subfamilies in the original version of fig. 1 when overexpressed in hESCs (fig. 3A), GATA6 and EOMES (2) display enriched binding at these subfamilies across models of post-gastrulation embryogenesis (fig. 3B) (3) segregate highly versus negligibly cis-regulatory integrants according to their binding profiles (figs 4 and S6) and (4) reveal canonical GATA binding sites from multiple sequence alignments on subfamily-restricted ChIP-seq peaks (figs 4 and S6), (5) in agreement with results from motif search

Of note, the GATA6 and EOMES-bound fraction of LTR6B integrants reached levels of effect sizes (activity = 1, i.e each gene close to an LTR6B integrant in the definitive endoderm is expressed with a fold change of 2^1 = 2 with respect to the mesendoderm) that we only witnessed for the LTR5 CRISPRa data (Fuentes dataset) reanalyzed in fig. 1, suggesting that subfamilies other than those exemplified in fig. 1 may reach important levels of effect sizes upon analysis with craTEs.

Moreover, we reanalyzed the RNA-seq data derived from LTR5-Hs/LTR5 CRISPRa/i, where we correctly recovered LTR5A and LTR5B subfamilies as highly cis-regulatory (fig. 1), while these were not relevant to the examples shown in fig. 1 in the original submission of the manuscript.

Finally, we included since the original submission the analysis of a dataset derived from the CRISPRi-mediated repression of LTR2B, which craTEs recovers as the most statistically significant, and the subfamily with the largest effect size (i.e activity coefficient).

We believe that these examples support the notion that craTEs performs equally well for subfamilies other than LTR5-Hs/SVA/LTR7, which were used to evaluate craTEs owing to their in-depth characterization as cis-regulatory elements, including CRISPRa/i experiments backed up with matched epigenomic assays without which evaluating craTEs would have proven difficult

4. The final claim that "TF binding may better predict TE cis-regulatory activity than differences in histone marks" is only based on two datapoints (KLF4 and ZNF611) and experiments involving the overexpression of the same factors. I worry that some of this does not really represent natural cis-regulatory activity.

We thank reviewer 2 for stating this concern regarding fig. 4 of the originally submitted manuscript. Using ChIP-seq data spanning three additional TFs (SOX15, GATA6 and EOMES) in experiments relying on endogenous TF expression levels, we show that TF binding appears better at separating functionally cis-regulatory vs inactive integrants than ChIP-seq readouts of chromatin status, i.e. ATAC-seq and H3K27ac (fig. 4 and S4).

Overall, I felt some of the claims were a bit overstated. See below for examples and additional comments.


P7L21. Fig 1A. I'm not sure I understand everything on this figure. What are the grey boxes? Transcripts?
We thank reviewer 1 for bringing this to our attention. We directly modified fig. 1A to better explain the schematics, and amended the legend accordingly.

P7L21. Why did you assume TEs within cis regulatory distance contributed to a significant fraction of the variation in gene expression? It is not clear what "significant" here means and within/relative to what larger context? (TF, epigenome, chromatin etc)

We thank reviewer 2 for requesting this important clarification. We added a precision: “*Thus, they are predicted to exert a similar cis-regulatory influence on protein-coding genes located in their vicinity,* ***for example through the simultaneous recruitment of a specific set of transcriptional regulators at multiple genomic loci*** *“*

We have confusingly used significant to mean “quantifiable” or “detectable”, whereas it is used in many other places in the manuscript as a shortened version of “statistically significant”. We have amended the manuscript accordingly: “*We thus assumed that the subfamily composition of TE integrants located within cis-regulatory distance of protein-coding genes contributes to a* ***discernable*** *fraction of the variation in gene expression.”* We systematically checked for and disambiguated potentially confusing uses of the term “significant” throughout the manuscript.

The statistical significance of the association can be measured by computing the overall F-test for significance (see for example Chapter 3 in *Introduction to Statistical Learning with Applications in R*, 2^nd^ Edition, ISBN-10 1071614177). We now report the overall F-test for significance for all experiments presented in fig. 1, and directly allude refer to it in the Results section: ***“The association between the differences in gene expression ∆ECRISPRi−control and the cis-regulatory susceptibilities of promoters to TE subfamilies N was statistically significant (g#1: p-val=5.47 · 10−146, F-test, g#2: p-val = 0, F-test), strongly suggesting an interrelation between changes in expression observed at protein-coding genes and the genomic distribution of integrants for at least a subset of all TE subfamilies.”***

P8L34. I found it hard to read the equation without knowing what some of the terms where. Why not define some of the terms (e.g. Delta A_0,2-1, Delta A'_m,2-1) and then show the equation? Was unclear to me why there was a ' . Also, why is epsilon_2+1 ? Overall I found the notation to be a bit confusing.

We thank reviewer 2 for highlighting what may be a confusing notational convention. We extensively simplified the notation throughout the presentation of *craTEs* in the Results section. We now present the equation after having properly defined all of its terms in the main text. The **‘** indicated the row and column-centered expression values, a detail now restricted to the Methods section. Regarding the subscript following the noise term epsilon, we amended the main text with an explanation: *“An independently and identically distributed Gaussian noise term centered around zero ε2+1 captures the variation in gene expression that is not accounted for by the linear model,* ***and represents the sum of the noise terms corresponding to gene expression in each condition.****”*

P8L60. What model is used to test against the null hypothesis. Is that model robust to the total number of elements in the subfamily?

As now explicitly stated in the Statistical methods paragraph of the Methods section, the standard t-test for regression coefficients (see chapter 3 in ISLR) is used to assess whether each TE subfamily significantly contributes to the variation in gene expression: **“***the statistical significance of TE subfamily activities is evaluated through null-hypothesis significance testing* ***via a t-test****, where the null hypothesis is H0 : the value of the associated linear regression coefficient (commonly referred to as β) is zero***.”**

This test only yields significant results given a sufficiently high signal to noise ratio. However, to ensure stringency, we ensured that at least 150 protein-coding genes participated to the estimation of each cis-regulatory activity coefficient, see: “*TE subfamilies with a sum of susceptibility scores smaller than 150 were removed from N*“ in the methods section.

Are you potentially only able to detect effects associated with smaller subfamilies like LTRs?

See our reply to reviewer 2’s first major comment.

Another question is whether the age of the subfamilies is a factor. Older families might have further diverged. Would you be able to detect signal associated with that or are you restricted to younger families like SVAs?

See our reply to reviewer 2’s first major comment.

If I understand correctly, you mainly analyzed the TE families with N>=150 and all genes >=10 reads for the model. Is it make sense to further filter out TEs with a short length? I am also wondering how many TE subfamilies were kept in the analysis. Is it also can be applied to subfamilies with a significant number of copies, like Alu subfamilies?

Since the best characterized mechanism accounting for the cis-regulatory activity of TEs is TF binding, and since TE integrants are larger than the few base pairs covered by the shortest TF binding motifs, we do not believe that filtering integrants by genomic length will improve the model. craTEs can be applied to subfamilies with large number of copies, such as Alus and MIRs and statistical significance may be reached (as now shown in fig. S2C), but the estimated effect size will decrease with the subfamily size, owing to the definition of the cis-regulatory activity estimates.

A researcher interested in studying a group of Alu sequences may attempt to separate them from the rest of their subfamily by directly modifying the susceptibility matrix N, following an approach similar to our “functional” TE annotation scheme.

P9L212. The sentence of "As TEs of the same subfamily share" was repeated multiple times in the manuscript.

We thank the reviewer for pointing out these redundancies. We modified the manuscript accordingly, replacing “share” by “**exhibit**” and “**phylogenetically related**”

P9L225. You mention correctly inferring the loss of enhancer effect, but what about the value and magnitude of these losses? Are they accurate and/or interpretable at all? Numbers, if at all interpretable, would make things more convincing.

We thank reviewer 2 for asking clarifications regarding to what we regard as an attractive feature of *craTEs,* namely that it provides interpretable estimates for the TE subfamily cis-regulatory activities. The interpretation of the activity coefficient is alluded to at several places in the manuscript, e.g: “*The negative activity values reflect the abrogation of the enhancer effect exerted by LTR5-Hs and SVAs in naïve hESCs by the CRISPRi system, and are best interpreted as the log2 fold-change in protein-coding gene expression attributable to the presence of a single integrant from the corresponding subfamily near the promoter of the given gene.”*

We added an explicit example regarding the first LTR5-Hs/SVA CRISPRi experiment discussed in the Results section: “*The negative activity values reflect the abrogation of the enhancer effect exerted by LTR5-Hs and SVAs in naïve hESCs by the CRISPRi system,* ***and are best interpreted as the log2 fold-change in protein-coding gene expression attributable to the presence of a single integrant from the corresponding subfamily near the promoter of the given gene. Specifically, the expression of any given gene bearing an LTR5-Hs integrant in its cis-regulatory window decreases according to an average estimated fold-change contained within the 95% confidence interval 2^-(1.96*0.0095) = [0.90;0.92] upon CRISPRi***.”

The accuracy of cis-regulatory TE activity can be evaluated thanks to the 95% confidence interval: the widest the confidence interval, the least accurate the estimated cis-regulatory activity.

We added all estimated cis-regulatory activities, together with their statistical significances, in Table S1.

P11L31. Looks like the character < is not displaying correctly.

We thank reviewer 2 for pointing this out. See our response to reviewer 1’s minor comment.

P11L42. I believe you refer to Fig Sup 1B here, a bit confusing. There's also no reference to Fig Sup 1A. Some comment below about Fig 1C which I think is incorrect. Even the figure caption was a bit unclear so I'm not sure I fully understand what was shown here. Perhaps you have more sensitivity, but it also looks like you have a higher rate of false positives. Perhaps your p-values are inflated?

We warmly thank reviewer 2 for flagging erroneous figure references throughout this section of the manuscript, which we have now corrected. In addition, we extensively reworked fig. S1, now systematically comparing the ability of craTEs and differential expression-based enrichment approaches to uncover a set of ground truth TE subfamilies defined according to complementarity to the CRISPRi gRNA and enrichment for gains in heterochromatin upon CRISPRi using AUC values. Our analyses indicate that craTEs is both more sensitive and more specific than DE enrichment based approaches. Furthermore, we now added a labeled volcano plot for differential expression under LTR5-Hs/SVA CRISPRi to aid in understanding the difference between what we call “stringent” and “lenient” DE gene calls.

P12L14. "are not surprisingly dropping" please rephrase

We thank reviewer 2 for requesting this clarification, and we modified the Results section accordingly: *“****Consistent with our findings while accounting for replicates (fig. 1C), LTR5-Hs and SVA subfamilies collectively exhibited a statistically significant decrease in cis-regulatory activity under CRISPRi across replicates, though not all of them passed the significance threshold.****”*

P12L37. "superior statistical power" what do you mean by superior here? Higher sensitivity?

We use the statistical definition of “statistical power”, which refers to the inverse of the probability of calling false negatives and is related to specificity. See our response to reviewer 3’s third major comment below.

P12L39. "reinforces the notion that TEs act as cis-regulatory fine tuners" I think this is a bit of a stretch for a result section since you didn't show anything like that.

The magnitude with which the null hypothesis {H0: all activity coefficients are zero} is rejected by the F-test of overall significance even when single replicates are considered (fig. S1A) strongly suggests that TE integrants act as cis-regulatory fine tuners that induce modest yet detectable changes in gene expression at scale. Otherwise, the linear model based on TE integrants would not be able to account for a statistically significant fraction of the variance in gene expression. Still, we amended the text to refine and tone down our claim: *“Overall, this case study suggests that craTEs is more powerful and more informative than DE-based enrichment approaches to discover cis-regulatory TE subfamilies from RNA-seq data,* ***supporting*** *the notion that TEs act as cis-regulatory fine-tuners, the dynamics of which may be overlooked when restricting the analysis to DE genes only.”*

P13L20. I think it makes sense to only look at genes on the same chromosome but if you do that, why go to 10Gb, instead of 1Gb, or even 100Mb? Seems like an overkill. I would be much more interesting to see a finer mesh of what is happening between 10Kb and 10Mb so that we could see the stability and where the actual minimum is. You point out yourself that this metric is unstable >100Mb, so why include it?

We thank reviewer 2 for suggesting we use a higher definition regarding the grid of cis-regulatory bandwidths considered when assessing the distance until which TEs measurably regulate protein-coding genes. We repeated the analysis presented in fig. 2D, this time using four values of L per order of magnitude (e.g L = 1e04, 2.5e04, 5e04, 7.5e04, 1e05 etc). Of note, the best cis-regulatory bandwidth reported in the resubmitted manuscript (L = 250kB) differs from that reported in the original submission (L = 100kB), simply as it was not present in the latter. Importantly, we propagated this change to all subsequent analyses in the paper (other panels of fig.2, as well as figs. 3 and 4), though this did not markedly change the estimated cis-regulatory activity values (fig. S4).

P13L35. "As 95% of the area under a gaussian curve is contained within two standard deviations from its mean, this suggests that TEs encode significant cis-regulatory information up to distances of approximately 200kb to 1 Million bases (Mb) from gene promoters" I'm not sure I fully understand this claim.

We thank reviewer 2 for asking for clarification on this important point. We simply pointed out to the well-known fact that most of the area contained under a Gaussian curve can be found in a window centered on 0, and extended by 2L on each side (https://en.wikipedia.org/wiki/68%E2%80%9395%E2%80%9399.7_rule). We modified fig. S2B to better explain how L relates to distance-weighting, specifically for the case when L = 250kB.

At this point, it also only applies to the few families that were tested.

We fully agree with reviewer 2’s comment, but would like to point out the optimal distance computation was also performed on an array of 441 transgene overexpression experiments (fig. 2D).

How significant is this contribution would also be important to quantify.

We did so by comparing the r-squared values for N matrices built using hard thresholded cis-regulatory windows of 100kB, 500kB and the weighted matrix N with L = 250kB which we found to be the most accurate on a distinct set of 441 transgene overexpression experiments (fig. 2D). Related to this, see our reply to reviewer 1’s third minor comment.

When the number of predictors does not change across models, i.e when the different N matrices contain the number of columns to tentatively model the same set of observations, the R squared value, i.e the proportion of variance explained, can be directly compared.

Also, shouldn't this extension of the model but tested on the 4 datasets from Fig 1 first before moving on to a new dataset?

Reviewer 2 may have overlooked the data presented in fig. 2B: the distance threshold approach was tested on four of the datasets presented in fig. 1.

P14L11. "the optimal bandwidth L took values between 50kb and 500kb", again I'm not sure this is so interesting since it's by design. A finer resolution of the optimal L within that range would be more interesting instead of discretizing in bins (50Kb, 100Kb, 500Kb, etc).

See our response to P13L20.

P14L58. "Both models thus use the exact same number of predictors, i.e cover the same TE subfamilies" ok but in the distance model you have extra parameters. I'm not sure the gain you make is as impressive as you claim.

The number of parameters in the model with fixed cis-regulatory windows and weighted-cis regulatory windows is the same, i.e the number of columns in N, which mirrors the number of cis-regulatory subfamilies considered. Distance was not optimized as part of the model fitting, as it was optimized on a separate (and much larger) dataset encompassing 441 transgene overexpression experiments. We did not overplay the gain in proportion of variance explained, simply noting that the “*distance weighting approach* ***better approximates*** *the cis-regulatory potential of TE subfamilies than the hard distance threshold approach.”* See above our reply to reviewer’s 2 comment: “How significant is this contribution would also be important to quantify*.”*

P15L6. "As the number of predictors in N remained unchanged" text would need some work, there's a lot of repetition in this section.

We thank reviewer 2 for suggesting a modification in the manuscript, however we feel like the unchanging number of predictors, ie columns of N, is paramount to our claim that the “*distance weighting approach better approximates the cis-regulatory potential of TE subfamilies than the hard distance threshold approach.”*. We would therefore favor risking being redundant rather than failing to make our point.

P15L18. Fig 2E. Why only this one instead of showing all examples from Fig 1C and a more direct side by side comparison of all the families that were observed to be significant using the previous model and the ones that are now significant here. Can you explain the patters that are associated with the families that gain significance? Do they tend to have higher L values?

We thank reviewer 2 for requesting this clarification. We showed the example pertaining to KLF4 overexpression as we previously showed that LTR7Y were directly bound by KLF4 in naïve hESCs or primed hESCs overexpressing KLF4 (Pontis et al., Cell Stem Cell 2019). However, LTR7Y were not included in the initial hard-thresholded version of the N matrix used throughout fig. 1, due to their relative paucity compared with (e.g LTR7 and LTR5-Hs) and the stringent filtering criteria we apply while building the matrix N that reviewer 2 has rightly mentioned in a previous comment (“If I understand correctly, you mainly analyzed the TE families with N>=150 and all genes >=10 reads for the model.”). To the best of our knowledge, the other experiments presented in fig. 1 did not involve subfamilies that remained absent in N constructed according to a hard threshold, as evidenced by the fact that the top statistically significant subfamilies remained so when using N built according to L = 250kB instead. We now explicitly show this in a new panel E in supp fig. 2 and amended the results section: “*Regarding the LTR5-Hs/SVA CRISPRi, ZNF611 overexpression and LTR2B CRISPRi experiments, using the distance-weighted matrix N still uncovered LTR5-Hs/SVAs, LTR2B, resp. SVAs as differentially cis-regulatory (fig. S2E)*”

Since TE subfamily recovery depends on which thresholds one applies statistical significance and/or effect size (thereby justifying the use of AUC values in the reworked version of fig. S1), changes in cis-regulatory family estimation really only matter near the top of the statistical significance scores, i.e for the smallest adjusted p-values. We observed such change for LTR7Y in the case of KLF4 overexpression, but not for the other experiments presented in fig. 1.

P15L39. "Having validated the ability of craTEs to reveal bona fide TE-dependent cis-regulatory activities" seems like a strong statement. Wouldn't be more "potentially regulatory active TEs"? It feels like the cis-regulatory activities themselves are unclear or black-boxes and more implied by the downstream result (activity shift).

We thank reviewer 2 for providing us with an opportunity to clarify this point. Indeed, we did not mean to claim that craTEs always discovers true cis-regulatory activities, but rather that previously characterized examples of TE-dependent cis-regulatory activity were successfully recovered by craTEs. We modified the text accordingly: “*Having validated the ability of craTEs to agnostically recover* ***well-established*** *cases of TE-dependent cis-regulatory activities.”*

P16L25. Fig 3B. I was not able to understand the color scale of this heatmap. How do these values compare to the ones you observed in the 4 experiments you used to validate the method. Are these as significant or marginal enrichment? I was also not able to find the underlying data in the supplements. Just a more comprehensive pdf.

We thank reviewer 2 for pointing out the lackluster presentation of fig. 3B in the originally submitted manuscript. We extensively reworked fig. 3B (now fig. 3A) to display both statistical significance and effect size of the estimated activities. The estimated activities for all TE subfamilies and all transgene overexpression experiments were readily available as Additional File 1, available from Zenodo as described in the original manuscript at lines 1139-1145, pp. 37-38. We nonetheless added a table (Table S7) restricted to the data underlying Fig. 3A and S4.

P16L25. Fig 3B. Related to the same figure, how much do the pattern change if you change the cutoff? Is it stable even if you 50Kb? Or 200Kb? You should show that most of the associations remain even if you change the cutoff slightly.

See our response to reviewer 2’s second major comment.

P16L25. Fig 3B. Related to this figure again, it would be good to confirm whether replicate showed similar results.

We now add fig. S5 which demonstrates consistency among replicates and refer to it in the text: “*Additionally, the directionality and statistical significance of TE-dependent cis-regulatory activities were robust to varying the cis-regulatory bandwidth L (fig. S3) and* ***consistent across replicates when analyzed individually (fig. S5).****”*

Would also be good to show more systematically that genes from the same pathways show more overlap in terms of effect versus unrelated genes.

We agree with reviewer 2 that under its previous form, fig. 3B did not permit a transparent evaluation of the similarities in cis-regulatory activity estimation across experiments. To palliate to this problem, we re-clustered cis-regulatory activities in fig. 3A and fig. S3C, which now better shows that TFs from the same subfamily tend to produce similar effects in terms of TE-dependent cis-regulation (see our reply to the next comment).

P16L34. "suggesting that commonalities in gene expression were partially mirrored by similar cis-regulatory TE activity patterns" here it feels like there are potential confounding explanations. Could it not be a distance thing? Are the genes from same family not often closer to each other? What about it being the result of TF cascade or interactions? Perhaps it is not due to the cis-regulatory TE activity patterns but rather affecting one element in turn affects the whole family.
a. I'd test whether these TF interact together
b. The distances between these elements
c. Whether they share binding site

We thank reviewer 2 for requesting clarifications on this part of the Results section.

a. Assessing whether TFs from the same subfamily “interact together” is out of the scope of this study and would be more aptly evaluated using a method capable of interrogating TF-TF interactions at scale, e.g mass spectrometry or yeast-two-hybrid assays. However, as shown in Fig. 5 of the original publication accompanying the dataset (Nakatake et al., Cell Reports 2020), TFs from the same subfamilies tend to produce resembling changes in gene expression, as explicitly illustrated for GATAs, DLXs, FOSs, KLFs and NEUROD/Gs. Since activities are estimated directly from the gene expression data, it follows that structurally related TFs that either tend to bind to similar motifs (see our reply to c below) or partake to the same complexes will produce resembling activity patterns.
b. We are unsure whether reviewer 2 here refers to related TFs or related TE integrants. In any case, what craTEs estimates is the genome-wide TE-dependent cis-regulatory response to transcriptional perturbations, in this case TF overexpression experiments. The most likely mechanism at play here is that *trans*-acting TFs bind to TE-derived regulatory sequences that exert their effect on gene expression in *cis*. The locations at which TFs bind TEs is independent from the locus at which their own gene is encoded. Lastly, we made sure to exclude artificially perturbed transgenes (e.g KLF4 in the KLF4 overexpression experiment) from the set of genes from which TE regulatory activities are estimated.
c. Investigating whether the 441 transgenes considered here share binding motifs and if so with members of the same subfamily is out of the scope of this study. We can however point to this excellent study by Ambrosini et al. showing that TFs from the same subfamily often share DNA binding motifs: <https://genomebiology.biomedcentral.com/articles/10.1186/s13059-020-01996-3>.

P17L35. "a MYB-LTR7 axis in the maintenance of self-renewal and stemness in adult cells and tissues" is this direct or indirect?

We have now included MYB ChIP-seq data, which displays enrichment at LTR7 in blood-derived cells.

Unclear to me whether most of what is observed is indirect.

We thank reviewer 2 for highlighting this weakness in the initial version of the manuscript. We now distilled a comprehensive reanalysis of the ChIP-seq data available from ChIP-atlas in the new fig. 3B to show that many of the TFs displaying increases in cis-regulatory activities in fig. 3A (previously, fig. 3B) display enriched binding at the elements they activate. Furthermore, we found published studies involving TF KO experiments for GATA2, GATA6 and SOX15, and show that these revert the cis-regulatory effects observed with endogenous TF levels (fig. 3C-D, S3D-F). Lastly, we document in improved versions of fig. 4 and the novel figure fig. S6 that direct evidence for TF binding via ChIP-seq singles out the most cis-regulatory fraction of the relevant TE subfamilies. Together, this data suggests that many of the examples highlighted throughout fig. 3, S3, 4 and S6 as well as in the text result from direct binding at the TEs.

P18L505. I could not see the increased AmnSINE1 activity for RUNX1 in fig.3B.

We warmly thank reviewer 2 for pointing out this mistake, which we duly corrected.

P18L483-488. A surprising contrast is mentioned but the implications are not clear. Is this a novel result?

We previously formulated the hypothesis that TEs exerting cis-regulatory effects in early embryogenesis may remain accessible and thus become co-opted at later stages of embryogenesis (see Pontis et al., Cell Stem Cell 2019, Pontis et al., Nature Communications 2022). Here, we expand upon this paradigm, notably by studying the effect of GATA6 KO on LTR6B-dependent cis-regulation and by highlighting a possible cooperation with EOMES for regulating these elements. In addition, we present both ChIP-seq and RNA-seq analyses supporting the notion that GATA6 binds to yet other primate-specific TEs subfamilies (MER4As, MER4Ds, PRIMA4-LTRs) with measurable effects on gene expression.

Does it change our current understanding of GATA6 expression? (discussion speculates a bit, but here its not clear what it entails.)

What we focus in our study are not GATA6 expression levels, which we do not directly study, but rather the TEs bound by GATA6 in the stages of embryonic development where it is expressed.

P19L522-524 you formed a functional group that seems to include repressive regions. Why not also the opposite, more active regions?

At this stage, combining epigenomics and expression data in *craTEs* is intended for maximally exploiting what may be considered as “given” information. As CRISPRi ultimately elicits chromatin compaction at bound loci, we believe that finding cis-regulatory changes in TE activity is more likely at sites where heterochromatin increases.

P20L556. "TF bindings may be better suited as a marker of cis-regulatory TE activity than changes in histone marks…". I am wonder is it specific to a certain strong TF? Or because this is based on an over-expression experiments? Have the authors test more TFs, like weak TFs or co-factors?

We thank reviewer 2 for requesting additional work regarding this claim. While the KLF4 and ZNF611 data initially presented in fig. 4 does rely on TF overexpression, we supplemented fig. 4 and added the new fig. S6 with a detailed analysis of GATA6, EOMES and SOX15 binding at TEs in contexts where these TFs are not overexpressed, which recapitulated our observations that TF binding better singles out cis-regulatory TEs than chromatin marks and/or accessibility. We are unsure to grasp what reviewer 2 refers to as “weak TFs”, but regarding the matter of cofactors, can point reviewer 2 to the fact that we found canonical GATA motifs at both EOMES and GATA binding sites in cis-regulatory TEs, but conversely could not find any EOMES motifs. This suggests that GATA6 may cause EOMES relocation at these elements. Still, EOMES binding appeared quite apt at marking cis-regulatory TE subfamilies, sometimes even performing better than GATA6 binding.

P21L594-596 "TE-dependent cis-regulation accounts for a fraction of the variation in gene expression comparable to that inferred from models of gene regulation based on the TFBS repertoire of core promoters." Where was that mentioned or shown?

We thank the reviewer for justly pointing out that this was only mentioned in the Discussion, slightly ahead of P21L594-596: “*We noticed that* craTEs *explains a fraction of the variation in gene expression ranging from approx. 2% to 12% in the experiments presented in fig. 1C. This is comparable to the proportion of variance in gene expression explained by previously published linear models of gene regulation based on putative TF binding sites at core promoters*”. We added fig. 1D, which depicts the obtained adj. R^2 for all experiments analyzed in fig. 1C, and explicitly refer to it in the Results section: “*Of note, the proportion of the variance in gene expression explained by the distribution of TEs across cis-regulatory windows [2% - 12%, adj. R2] (fig. 1D) overlapped with the typical fraction of variance reported upon modeling gene expression as a function the distribution of TF binding motifs at gene promoters.*”

P30L835. A typo in the sentence of "To ensure that that…"

We thank reviewer 2 for pointing this out, and duly corrected the manuscript.

P33L908. I am wondering is it make sense to generate the corresponding R script to perform such analysis? It would be much more valuable if the authors could convert them to easy-to-use R/python scripts.

We thank reviewer 2 for what is an excellent suggestion. We have now published *craTEs* as an R package, available from github at: <https://github.com/bopekno/craTEs>

Of note, all of the code and data required to reproduce our analyses are available on the RENKU platform for reproducible data science funded by the Swiss Institutes of Technology under the form of code and containerized environments, as already indicated in the first version of our manuscript: *“The code used to process the data and generate the figures can be found and executed directly from the renkulab platform for reproducible data science at* [*https://renkulab.io/gitlab/crates*](https://renkulab.io/gitlab/crates)*.”*

Reviewer #3:

Pulver et al. introduce a new method, called craTE, to infer the TE subfamilies that are controlling gene regulation. The method takes advantage of the multiple copies of TE in the genome to provide a good statistical power ensuring the analysis can be run with even a single replicate. The analysis is able to reproduce some already known results. The comparison with studies that integrated transcription of TEs shows that craTE has a higher sensitivity and is able to detect previously missed regulatory TEs. The authors test several distances and find that the TEs impact gene regulation on distances between 100-500Kb. Also, they find that TF binding at TE is more important than histone modifications. Overall, I find this paper interesting, and would benefit the field. Nevertheless, there are some outstanding points that the authors will need to address.
1. The introduction is too long. Maybe it could be shortened to focus on the main important aspects.

We thank reviewer 3 for this suggestion and have shortened the introduction.

2. There is no implementation of the method shared. The authors should include a github/package link for the method that could be used by the community.

See our response to P33L908 by reviewer 2.

3. The benchmarking with other approaches is not systematic. It is not clear what metrics have been used to compare with other methods. What is a true positive, false positive, false negative and true negative?

We thank reviewer 3 for raising this fair point. We have now extensively reworked the analysis presented in fig. S1, where we decided to define a set of ground truth-cis-regulatory TE subfamilies using two criteria: (1) complementarity with the gRNA used in the corresponding CRISPRi experiment and (2) increased heterochromatin marks and/or decreased chromatin accessibility upon treatment with CRISPRi. Of note, epigenomic data was only available for one of the two gRNAs, we thus profiled chromatin accessibility in g#1-mediated LTR5-Hs/SVA CRISPRi of naïve hESCs and now include that data in the GEO entry accompanying this manuscript. This yielded a set of negatively cis-regulatory TE subfamilies for each anti- LTR5-HS/SVA gRNA.

Here are the sets of true positives and true negatives for both gRNA experiments:

- g#1: TP: {LTR5-Hs, SVA-A, SVA-B, SVA-C, SVA-D}. TN: all other TE subfamilies
- g#2: TP: {LTR5-Hs, SVA-A, SVA-B, SVA-C, SVA-D, SVA-E, SVA-F}. TN: all other TE subfamilies

Specifically, we consider a false positive as a subfamily that wrongly gets assigned to a differential cis-regulatory activity, a false negative as a subfamily that wrongly does not reach statistical significance, despite being cis-regulatory in real life, a true positive as a TE subfamilies that indeed exerts cis-regulatory effects on neighboring genes and true negatives as TE subfamilies that do not display any context-dependent change in cis-regulatory activity.

As considering TE subfamilies as “sufficiently importantly” cis-regulatory depends on thresholding on statistical significance and/or effect size, we used AUC to systematically compare craTEs with competing approaches in the task of recovering truly cis-regulatory TE subfamilies and used 1-(BH adj. p-values) as the probability of being classified as cis-regulatory.The AUC takes values between 0.5 and 1 and can be interpreted as the probability of having correctly ordered observations such as to separate classes perfectly. Here, a perfect AUC of 1 would be reached in cases where ranking the adj. p-values given by craTEs ranks all TE subfamilies found in the ground truth among the subfamilies with the most statistically significant changes in activity. An advantage of the AUC is that it allows for a detailed study of the relationship between sensitivity and specificity as the threshold for classification varies.

We amended the methods section with these explanations and now discuss these new results in the main text.

4. One of their main finding is that TEs need to be within 500Kb to have an impact on gene activity. This is very interesting, since that would be the size of most TADs. TADs have been shown to have a regulatory role (https://genome.cshlp.org/content/32/4/682.short; https://www.biorxiv.org/content/10.1101/2022.04.29.488986v2.abstract). The authors should test whether including TAD data will improve their predictions. Instead of using a fix size, could using the specific TADs improve the results.

We thank reviewer 3 for making this suggestion. We now analyze restricting TE-protein coding gene regulatory interactions as intra-TAD. However, restricting cis-regulatory assignment to hESC-specific TADs did not improve the proportion of variance in gene expression explained by craTEs for the majority of the overexpressed transgenes (fig. 2D), though it should be noted that the error obtained under TAD-informed cis-regulatory weighting was often very close to that obtained under TAD-agnostic cis-regulatory weighting. We amended the Results section as well as the Discussion to directly address those points.

5. The authors found that specific histone modifications do not perform as well as TF ChIP data. This is not surprising since we know that most regulatory regions are characterized by combinations of histone modifications (https://genomebiology.biomedcentral.com/articles/10.1186/s13059-021-02532-7). The authors could add an explanation about this.

We thank reviewer 3 for this pertinent suggestion. We amended the discussion accordingly. **“*Surprisingly, we found that across various experimental systems entailing overexpression, KO and endogenous TF expression, TF binding was better able to enrich for cis-regulatory integrants than changes in histone marks and/or chromatin accessibility, though how TF binding compares with context-matched chromatin states as defined from combinations of histone marks remains to be seen.*”**

Generating multiple ChIP-seq histone modifications would be beyond the scope of this manuscript, but authors could try to use chromatin state data to see if that improves predictions.

We thank reviewer 3 for this suggestion. While this may be an interesting avenue for segregating functional vs non-functional fraction of TE subfamilies, we would like to insist here on the sensitivity of this procedure to the choice of RNA-seq vs ChIP-seq data. Indeed, we observed that the splitting of TE subfamilies into functional vs non-functional integrants was most useful when RNA-seq and CUT&TAG/ChIP-seq data were derived from the exact same experiments. We thus fear that using chromatin states generated from distinct datasets may not be a fair comparison to the matched TF binding data.

Reviewer #4: Remarks to reviewers and editors:
Pulver et al. demonstrate the cis-regulatory potential of transposable elements (TEs) families from RNA-seq datasets by analyzing the expression of protein-coding genes in this manuscript. This is essential because cataloguing the cis-regulatory TE families from RNA-seq data alone was not thoroughly investigated before. Thus, this line of advancement has enormous potential to outreach plenty of genome biology researchers and other audiences. The authors also interpret a fascinating insight where even the expression from the TE sequence fails to provide a more accurate cis-regulatory potential of the given TE families compared with the algorithm presented here. Authors formulated a mathematical model of gene regulation assuming that a bunch of cis-regulatory TE loci from subfamilies regulate the neighbour gene expression. Their algorithm did not employ complex deep learning algorithms but a straightforward arithmetic formula to decipher the cis-regulatory association of TE families with their neighbour genes by curating the genome annotation information and coupling it with the expression values of protein-coding genes. They call these families' craTEs'. This work indeed represents a significant advance over previously published studies.
The first section of the manuscript describes a computational analysis of craTEs using multiple published datasets where functional genomics experiments were available. Using the gene expression profiles from these datasets, they correctly infer the loss of enhancer effect of target TE families in CRISPRi/overexpression of KRAB-ZNF experiments and gain of their enhancer potential when they analyze KLF4 transcription factor (TF) over-expression experiments. Following these leads, authors also calculate the distance from which TE subfamilies are likely to act as cis-regulatory elements. They found that these TEs can have cis-regulatory potential even when situated ~200 kb away from the promoter of their target genes.
The second section of the manuscript describes the computational analysis of human gastrulation/organogenesis from in vitro differentiation models of human embryonic stem cells (hESCs). To pinpoint the TFs driving these patterns, the authors analyze a published dataset of thousands of hESC lines overexpressing one of 441 TFs. Based on these data, the authors use their in-house algorithm to establish a correlation between the cis-regulatory potential of specific TE subfamilies targeted by subsets of TFs. With this, the authors attempt to assess the cis-regulatory potential of TEs in response to TFs targeting them in preimplantation embryonic cells that further extends to and beyond gastrulation stages. Based on these data, the authors propose an attractive model where sequence-specific TFs are expressed in hESCs and activate the cis-regulatory potential of cell type-specific subsets of TE families, activating their adjacent genes. These TFs contribute to modulating TE-derived enhancers in subsequent developmental lineages, thereby shaping the transcriptional networks of fetal development. Lastly, they overlay their analysis with ATAC-seq and ChIP-seq datasets to add another layer of observations where the TE subfamilies can further be divided into active and inactive sets of cis-regulatory loci.

A few major concerns make me less optimistic about this work getting published in its current form.

Major Concerns:

While this is a fascinating mathematical model which seems to work just fine, the study generally falls short due to the inaccessibility of code or a computational tool to attest to the transparency and reproducibility of their analysis. The algorithms developed here are rich, exciting and extremely valuable because this method requires fewer data to find the regulatory potential of TEs; once it is out in public, the readers might want to use it to make the best use of their transcriptomic data. However, this algorithm is used only in early embryonic and cancer cells. Unfortunately, it is impossible to assess their analysis's validity because they do not provide access to their scripts. Specific codes used for their analysis should be made available on Github for example.

See our reply to P33L908.

It should be available as a package or code to test on other data sets that are not naïve or cancer cells, which might be recommendable.   

Furthermore, we expanded the scope of analyzed RNA-seq datasets beyond cancer and naïve hESCs (see the novel versions of figs. 3, S3, 4 and S6), by focusing on specific examples of post-gastrulation embryogenesis, e.g human primordial germ cell-like cells (hPGCLCs), mesendodermal and endodermal cells, as well as hematopoietic progenitor cells (HPCs).

The cis-regulatory potential is attributed to the locus depending on the chromatin environment around it. The authors indeed performed a comparative analysis of their algorithm with the ones published from Bourque lab where they had analyzed a set of RNA-seq data. From this analysis, the authors show convincingly that their algorithm predicts the more reliable strength of cis-regulatory potential by a greater margin. At the family level, there are published materials from Didier Trono, Cedric Feschotte and a few more labs demonstrating the significance of enrichment of TE families in cis-regulatory repertoire of analyzed datasets. However, it is unclear whether their algorithm outperforms the existing methods for predicting the cis-regulatory potential of a given TE family. Despite, they are entirely different methods and require a distinct set of raw data, the level of statistical significance must be compared to illustrate the pros and cons of their algorithm.

We warmly thank reviewer 4 for suggesting to measure the performance of craTEs against methods based on enrichment of chromatin marks at TEs. We implemented this in the analysis presented in fig. S1, where we leveraged differences in ATAC-seq and H3K9me3 signal to label a ground truth of differentially cis-regulatory TE subfamilies upon CRISPRi. craTEs displayed a generally high agreement (AUC between 0.88 and 1), even when monoplicates were analyzed individually.

Of note, we would caution against considering gRNA-defined DNA sequence complementarity as a ground truth against which craTEs and epigenomic enrichment approaches may be evaluated. Indeed, we believe that similar to other experimental perturbations relying on DNA sequence complementarity (e.g RNAi), CRISPRi data requires a degree of validation, typically using epigenomic enrichment approaches, to be taken at face value. Thus, epigenomics enrichment data are instrumental in defining the ground truth set of subfamilies against which craTEs and differential expression analysis based methods are evaluated. Comparing the output of craTEs and epigenomics data against a ground truth defined in such a way would prove circular.

In addition, we have amended the Discussion with a comment regarding the tradeoffs regarding the use of craTEs vs enrichment based approaches: “***One putative limitation of craTEs compared with enrichment approaches stems from the fact that it estimates exactly one activity coefficient per TE subfamily. Consequently, craTEs may fail to recover cis-regulatory TE subfamilies encompassing integrants exerting antagonistic cis-regulatory effects. In that case, enrichment approaches may benefit from treating increased and decreased expression and/or epigenomic signal separately, given an adequate sample size.”***

When authors apprehend the catalogue of TEs to investigate craTEs, they merge the neighbour locus from the same family. This method is also not very transparent to me. What do they do about those loci where an LTR sequence from another family is nested within the two TEs of the same family?

See our reply to comment # 2 by reviewer 1.

The chosen datasets and the pieces of literature cited here to support their claims seem selective. For instance, why was Fuentes et al. (PMID: 30070637) not selected for validating their algorithm? This study demonstrated both CRISPRi and CRISPRa experiments serving as forward and reverse experiments to determine the cis-regulatory potential of LTR5_HS. Moreover, this study has a plenty of RNA-seq data publicly available for the analysis.

We thank reviewer 4 for suggesting the analysis of this CRISPRa/i dataset. The corresponding results are now presented in fig. 1, and suggest that craTEs aptly recovers cis-regulatory TE subfamilies (LTR5-Hs, LTR5A and LTR5B) in that context.

The analysis of the TF overexpression data is purely correlative, and as such, it fails to establish a direct functional link between particular TFs and TEs. Toward this goal, this analysis could be complemented by analyzing ChIP-seq data for TF binding to the TEs as well as enrichment analyses for corresponding TF binding motifs within the sequences of the TEs.

We agree with reviewer 4 that though useful for hypothesis generation, the data presented in fig. 3A (previously fig. 3B) on its own does not suffice to conclude at the involvement of post-gastrulation-specific TFs in the regulation of gene expression via primate-specific TEs. We thus added fig. 3B, where we documented enriched TF binding at the TE subfamilies displaying increased cis-regulatory activity upon overexpression of said TF in cellular models of gastrulation and differentiation into germ layer. We also uncovered several canonical GATA motifs at bound regions of LTR6B and other primate-specific TEs. Finally, we found publicly available RNA-seq datasets where the corresponding TFs (GATA3, GATA6, SOX15 ) were knocked out instead of overexpressed, which inverted the estimated cis-regulatory activities uncovered upon overexpression.

Regarding a discussion of the correlative nature of the evidence presented, we point reviewer 3 to our reply to comment “P17L35” by reviewer 2, particularly the part “Unclear to me whether most of what is observed is indirect.”

There are certain instances from the analysis of the TF overexpression data in figure 4B and additional file 2 (heatmaps) which does not make sense. For example, KLF4 seems not to enrich over LTR7 (not the same as what was shown in Ohnuki et al., (PNAS) and Carter et al., (eLife)).

We thank reviewer 4 for pointing out that in our reanalysis of the Nakatake dataset, overexpression of KLF1, KLF2 and KLF4, though associated with an increased cis-regulatory activity (importantly, fig. 3A does not display enriched binding) for LTR7Y LTR5-Hs and SVAs and thereby matching the results from our thorough characterization of KLF4 expression in hESCs (Pontis et al., Cell Stem Cell 2019), associated with a decrease in LTR7 cis-regulatory activity. This indeed comes as a peculiar observation, against the fact that we (Pontis et al., Cell Stem Cell 2019, this manuscript, fig. 1, fig. 3B) among others (Ohnuki et al., PNAS, Carter et al. Elife 2022) have reported at several occasions that KLF4 binds to and transcriptionally activates LTR7 in human embryonic and pluripotent stem cells.

However, KLF4 has also been characterized as a master regulator of endoderm differentiation (e.g terminal differentiation of goblet cells Katz et al., Development 2002). It is thus possible that Natakake et al’s hESC cell line (SEES3, not the canonical H1 cell line analyzed in Carter et al., 2022) reacts to rapid and elevated overexpression of KLF1, KLF2 and KLF4 by triggering some differentiation-specific mechanisms, thus polarizing them away from the ESC state. Supporting that observation is the fact that unlike induction of KLF5, which leads to an “ESC” visual phenotype after 3 days of induction, induction of KLF1, KLF2 and KLF4 respectively lead to “spiky”/spiky/“floating, proliferation or died” phenotypes (Nakatake et al., Cell Reports 2020, Table S1). Accordingly, craTEs uncovers a KLF5-dependent *increase* in LTR7 activity, and KLF1, KLF2 and KLF4-dependent *decreases* in LTR7 activity. Note that the data presented in Fig 3 A was taken 48h post induction, i.e 24h prior to phenotyping by visual inspection. We modified the manuscript accordingly: “*Overexpressing KLFs collectively increased the cis-regulatory activity* ***of LTR7Y*** *elements in agreement with previous studies characterizing KLFs as inducers of* ***LTR7Y*** *enhancer activity in naïve hESCs.* ***Interestingly, induction of KLF5 - but not of KLF1, KLF2 and KLF4 - increased the cis-regulatory activity of LTR7, matching visual inspections which revealed that among these, only KLF5-overexpressing cells retained an ESC-like phenotype 72 hours post-induction****”*

The argument of POU5F1 over-expression not associating with any families cis-regulatory potental also applies for the KLF4, WNT3 etc., that are already expressed in hESCs.

In the original manuscript, we insisted that a peculiarity of POU5F1 in hESCs is that it is already “highly expressed”, not merely expressed. Here, we would like to point out that KLF4 levels are actually much more elevated in naïve versus primed hESCs (e.g. see Fig 2e in Warrier et al., Nature Communications 2017). Still, we toned down our claim: “*Interestingly, overexpression of the core pluripotency TF POU5F1 (also known as OCT4) was not associated with differential TE cis-regulatory, suggesting that overexpressing an already highly expressed gene, namely POU5F1, in a cellular context that largely relies on it, i.e. primed hESCs,* ***may not necessarily alter TE-dependent cis-regulation.***”

I am not aware that WNT3a binds on DNA anyway.

This caveat was implied in the original manuscript: “*We therefore applied craTEs to the ”perturbome” dataset, where* ***441 transgenes, most of them TFs****, were individually overexpressed in primed hESCs for 48 hours through a dox-inducible system*” We thank reviewer 4 for pointing out that we at some occasions mistakenly referred to the set of overexpressed transgenes in in fig. 3A as “transcription factors”. We amended the text accordingly, e.g “*To reveal how TE-dependent cis-regulation relies on TF****/transgene*** *overexpression in hESCs*”. We also corrected the legend for figure 3.

Also, GATA3 is placenta/trophectoderm-specific TF in this developmental window. So, why the SVA and LTR5_Hs would enrich significantly in GATA3 OE cells, Do these elements have GAATA binding sites?

We now report in fig. 3B that GATA family members collectively exhibit enriched binding at LTR5-Hs and SVAs in hESC-derived primitive streak cells as well as endoderm and PGC lineages cells. Furthermore, we document the presence of multiple canonical GATA binding motifs at GATA6-bound LTR5-Hs in the mesendoderm in fig. 4F.

A recent study (Branco and Ming lab) has analyzed the cis-regulatory TE families from GATA3 OE cells, I am not sure if they also found LTR5_HS, SVA.

Indeed, Du et al. Genome Research 2023 report enriched GATA3 binding at LTR5-Hs. SVAs are not reported in their study, though it is not clear whether that is due to low/no enrichment of repeat filtering.

My intention is not to convey an impression that analysis is wrong, but if authors are showing these data then it would be friendly to readers if they discuss these disparities.

Minor Concerns:

When authors split the families into functional and non-functional, they specifically demonstrate one of the youngest TE families. The non-functional ones did not show the ChIP-seq or ATAC-seq marks or vice-versa. Here, the authors did not show if these groups were equally mappable. The authors provide no details of quality control measures taken for analyzing these TE from sequencing data. Youngest families peak calling from ChIP-seq and ATAC-seq data is challenging and requires robust control of batch effects, mapping inaccuracies, normalization, and more.

We thank reviewer 4 for pointing out the issue of mappability at evolutionarily young integrants. To assess which data may be affected by a mappability bias, we leveraged genome-wide pre-computed mappability tracks for 50bp single end, 76bp paired end and 100bp paired end reads (Sexton and Han, Mobile DNA 2019), matched them to the read lengths of the NGS data we re/analyzed and computed the average mappability at each integrant belonging to subfamilies we previously found to be cis-regulatorily relevant. We did observe a bias towards lower mappability at “functional” LTR5-Hs, LTR7 and SVAs upon CRISPRi against LTR5-Hs/SVAs and KLF4 overexpression (fig. S7). However, the more pressing issue is whether this bias explains the previously estimated differences in cis-regulatory activities observed at functional vs. non-functional integrants (e.g fig. 4). We thus re-estimated functional vs. non-functional cis-regulatory activities, having beforehand split the relevant subfamilies according to average mappability. This allowed us to directly compare the estimated cis-regulatory activities for functional vs. non-functional integrants displaying equivalent levels of mappability. The sharp divide between functional (generally higher effect size in absolute terms, generally higher statistical significance) vs. non-functional integrants remained at low mappability (fig. S7), suggesting that mappability does not confound functional integrant calling.

Again, it should be provided with scripts for accessibility, transparency and reproducibility purposes.

See our reply to comment P33L908 by reviewer 2.

The rationale for the given algorithm in this paper is that TEs from the same family exhibit similar sequences and thus might exert a similar cis-regulatory potential. The notion of sequence-similarity is evoked throughout the paper. Still, it is not shown whether the functional and non-functional TE loci within the same family exhibit some sequence dissimilarities. The mechanism of these subgroups exerting a distinct regulatory potential remains a matter of speculation until a sequence similarity approach is undertaken.
We thank reviewer 4 for raising concerns regarding mappability at young integrants. Motif search, such as performed using FIMO, is not affected by mappability issues. Were mappability to be an issue, one would expect to find many integrants carrying a motif for the TF but no ChIP-seq peak due to mappability issues.

From the numbers presented in fig. S6 C, regarding GATA6 peaks and GATA6 motifs at LTR6B. There are 152 LTR6B integrants considered by craTEs. (66+14+9+1+1)/152 = 91/152 carry a GATA6 motif. (66 + 9 + 8 + 1 + 1)/152 = 85/152 carry a GATA6 peak. Overlap between the two: (66 + 9 + 1 + 1) / 152 = 77/152. Mappability issues may arise at integrants carrying a GATA6 motif but no GATA6 peak which happens at 19+14 = 33 / 152 integrants.

Authors predict the enhancer scores using a correlative model from TF over-expression data; however, existing ChIP-STARR-seq data is available. Wouldn't it add another reliable layer to their analysis since it is a more direct measurement of the enhancer potential of the TEs?

We thank reviewer 4 for suggesting what would be an interesting source of data which may complement *craTEs* for defining functional vs. non-functional integrants within subfamilies, much like ChIP-seq/CUT&TAG/ATAC-seq data. An advantage of STARR-ChIP-seq over standard ChIP-seq, is that it assesses gene transcription at a target – though exogenous –promoter located downstream of the tested genomic fragment. However, fragment length (typically between 200-500bp) may not be sufficient to exhaustively capture the enhancer potential of TEs, whether them LTRs (~600bp in length) or longer (e.g. SVAs, up to 3-4kb). Assessing the extant to which existing STARR-ChIP-seq data may prove useful in the context of validating enhancer effects at the level of entire TE subfamilies would entail additional work, including getting a sense of how well STARR-ChIP-seq data covers the different subfamilies. In addition, while STARR-ChIP-seq excels at characterizing enhancers within cell states, it it not intrinsically well equipped at characterizing changes in enhancer states across cell states, although this has been done in the past (Barakat et al., Cell Stem Cell). We have amended the Discussion: “*Alternatively, craTEs may benefit from the incorporation of STARR-ChIP-seq data, though whether fragment length and genome coverage prove appropriate for studying TE-mediated cis-regulation should be assessed*.”

Critical Appreciation:

The authors performed many clever analyses and processed impressive data, but the patterns drawn from these analyses remain correlative. The biological relevance of the inferences drawn here fails to provide robust and direct evidence supporting the significant claims, and thus, the manuscript feels diffuse and exploratory rather than conclusive. Following these concerns, I should not recommend this manuscript for publication in Genome Biology.

We thank reviewer 4 for providing us with constructive feedback. We have now included reanalyses of ChIP-seq data that suggest direct and context-specific TF binding at cis-regulatory TE subfamilies throughout gastrulation and beyond. Moreover, we found that knocking out several of these TFs across different lineages of the differentiating embryo (GATA2 in HPCs, GATA6 in meso/endoderm, SOX15 in PGCs) reverted the cis-regulatory effect that we had attributed to them using craTEs and epigenomic data. Importantly, refining craTEs using the matched TF binding data accurately segregated functional from non-functional TE at these subfamilies and markedly increased the strength of the signal captured by craTEs. Lastly, we documented the presence of several GATA motifs underneath GATA6 ChIP-seq peaks at functional LTR6B, LTR5-Hs and other cis-regulatory primate-specific TE subfamilies. In our view, this data strongly suggests that core TFs controlling gastrulation and germ-layer formation bind primate-restricted TEs - some of which notably serve as enhancers in early embryogenesis (LTR7, SVAs, LTR5-Hs, see Pontis et al., Cell Stem Cell 2019) – with the ensuing consequence of affecting the expression of protein-coding genes in cis.

Also: see our reply to comment P17L35 by reviewer 2.

**Second round of review**

**Reviewer 1**

-

**Reviewer 2**

I've read with interest the detailed point-by-point response. Overall, I feel the authors have thoroughly addressed all my concerns, and similarly addressed other reviewers' concerns. I was quite happy to see the new Fig 3, one of the main results of the paper, which I found to be much improved. Relative to one of my first major points about their ability to detect effects in families of different sizes and ages, I found the new Fig S2 C-D to be reassuring, although I would have also liked a more detailed characterization of your power to detect signal based on those parameters. Maybe this could have been done via simulations. Anyways, it's not critical at this point given everything that's already included in the manuscript.

**Reviewer 3**

The authors have addressed my comments.

There only thing they need to clarify is that in the revised version to my comment 3, they report an AUC of 1.0. This is likely from the small number of true positives and, while there is little they can do here, they should clarify it in the text.
